# The Interactions between Ionic Liquids and Lithium Polysulfides in Lithium–Sulfur Batteries: A Systematic Density Functional Theory Study

**DOI:** 10.3390/ma17112689

**Published:** 2024-06-02

**Authors:** Chengren Li, Nan Zhou, Rongde Sun, Jiaxin Tang, Jianglu Liu, Jianhua He, Changjun Peng, Honglai Liu, Shaoze Zhang

**Affiliations:** 1 National Engineering Research Center of Vacuum Metallurgy, Kunming University of Science and Technology, Kunming 650093, China; 2 Engineering Laboratory for Advanced Battery and Materials of Yunnan Province, Kunming University of Science and Technology, Kunming 650093, China; 3 Key Laboratory for Advanced Materials, School of Chemistry & Molecular Engineering, East China University of Science and Technology, Shanghai 200237, China

**Keywords:** ionic liquids, lithium–sulfur batteries, lithium polysulfides, interactions

## Abstract

Ionic liquids (ILs) based on hybrid anions have recently garnered attention as beguiling alternative electrolytes for energy storage devices. This attention stems from the potential of these asymmetric anions to reduce the melting point of ILs and impede the crystallization of ILs. Furthermore, they uphold the advantages associated with their more conventional symmetric counterparts. In this study, we employed dispersion-corrected density functional theory (DFT-D) calculations to scrutinize the interplay between two hybrid anions found in ionic liquids [FTFSA]^−^ and [MCTFSA]^−^ and the [C_4_mpyr]^+^ cation, as well as in lithium polysulfides in lithium–sulfur batteries. For comparison, we also examined the corresponding ILs containing symmetric anions, [TFSA]^−^ and [FSA]^−^. We found that the hybrid anion [MCTFSA]^−^ and its ionic liquid exhibited exceptional stability and interaction strength. Additionally, our investigation unveiled a remarkably consistent interaction between ionic liquids (ILs) and anions with lithium polysulfides (and S_8_) during the transition from octathiocane (S_8_) to the liquid long-chain Li_2_S_n_ (4 ≤ n ≤ 8). This contrasts with the gradual alignment observed between cations and lithium polysulfides during the intermediate state from Li_2_S_4_ to the solid short-chain Li_2_S_2_ and Li_2_S_1_. We thoroughly analyzed the interaction mechanism of ionic liquids composed of different symmetry anions and their interactions with lithium polysulfides.

## 1. Introduction

Ionic liquids (ILs) have been established as a reliable alternative to traditional organic electrolytes owing to their exceptional properties, including robust thermal stability, an expansive electrochemical range, low volatility, and non-flammability [1,2]. Bis(trifluoromethyllsulfonyl)imide [TFSA]^−^, characterized by two symmetrical sulfonylimide anions, is widely acknowledged as the predominant anion in ionic liquid electrolytes [3,4,5,6]. However, these ILs present notable drawbacks when compared to standard organic carbonate-based electrolyte solvents, such as heightened viscosity and a restricted liquid temperature range [7]. Consequently, the challenge of reducing the viscosity and melting point of ILs poses a significant hurdle for their practical application in next-generation batteries and capacitors [8].

To overcome the limitations associated with symmetric anions in ionic liquid electrolytes, the addition of asymmetric or compound anions has been proposed [9,10]. These novel anions retain the advantageous properties of their symmetric counterparts while demonstrating potential in inhibiting crystallization and reducing the melting point [11]. Notably, the hybridization of bis(fluorosulfonyl)imide [FSA]^−^ and [TFSA]^−^ has led to the formation of fluorosulfonyl(trifluoromethanesulfonyl)imide [FTFSA]^−^, effectively preventing the crystallization of ILs at ambient temperatures and expanding their liquid temperature range [12,13,14]. Palumbo et al. synthesized ionic liquid (IL) electrolytes, including the asymmetric [FTFSA]^−^ anion, in combination with the N-trimethyl-N-butyl-ammonium [N1114]^+^ and N,N-diethyl-N-methyl-N(2-methoxyethyl)-ammonium [N122(2O1)]^+^ cations, and observed remarkable electrochemical stability in the resulting [FTFSA]^−^ electrolytes [15]. Subsequent investigations into the electrochemical and physicochemical properties of these electrolytes in lithium-ion batteries, supercapacitors, and other electrochemical capacitors have yielded favorable outcomes [12,13,16].

Furthermore, the recent synthesis of a new asymmetric sulfonylimide anion, namely, methylcarbonate(trifluoromethylsulfonyl) imide [MCTFSA]^−^, representing a synthesis of triflamide and carbonate, has produced promising results. The physicochemical properties of its sodium and N-butyl-N-methyl pyrrolidinium salts have been explored, accompanied by structural insights obtained through X-ray diffraction studies of the sodium salt. These investigations have been discussed in terms of charge delocalization, coordination chemistry, and electrochemical behavior, revealing encouraging findings [11,17].

The sluggish transformation kinetics of lithium polysulfides (LiPSs) and the adverse shuttle effect resulting from the accumulation of highly soluble LiPSs are widely recognized as the primary impediments to the practical implementation of lithium–sulfur batteries [18,19,20,21]. The generation of soluble long-chain LiPSs during battery operation leads to immediate corrosion at the lithium anodes, causes rapid capacity decay, and undermines cycle performance. Additionally, the poor conductivity of the active S_8_ clusters and the final discharge products of Li_2_S_2_/Li_2_S limits the utilization of sulfur species and diminishes the discharge capacity. Furthermore, the sluggish redox kinetics during the charging and discharging processes, attributed to high potential barriers in the rate-limiting steps (Li_2_S_2_ → Li_2_S), constrains the high-rate charging and discharging capabilities of Li–S batteries [21]. The sluggish sulfur reduction reaction (SRR) kinetics worsens the polysulfide shuttle, leading to inadequate sulfur utilization [22,23,24].Previous investigations have demonstrated that incorporating LiNO_3_ into the electrolyte of lithium–sulfur batteries simplifies the reduction of LiNO_3_ to LiNO_x_ on lithium electrodes during discharge. This process yields LiNO_x_ species capable of further oxidizing lithium polysulfides within the lithium surface layer to form LiSO_y_, thereby effectively passivating the lithium surface [25,26,27,28]. This passivated surface effectively mitigates the shuttle reaction of polysulfide ions on the lithium electrode, leading to a substantial enhancement in the electrochemical efficiency and utilization of lithium–sulfur batteries [27]. However, as the nitrate additive is gradually depleted, the shuttle effect reappears [29]. Compared with other cathode materials, the S in lithium–sulfur batteries is abundant and inexpensive, and because lithium–sulfur batteries have a high energy density, we must not give up on the exploration of lithium–sulfur batteries [30,31,32,33].

The inherent stability of ionic liquids (ILs) means that they are not depleted during the charge/discharge process. In addition, the IL-based electrolytes possess the capability to diminish the solubility of lithium polysulfides (LiPSs), in contrast to conventional organic electrolytes [34,35,36,37,38,39]. Ionic liquid lithium–sulfur batteries may face higher costs in production, mainly because of the higher price of ionic liquids and the complexity of the production process. However, with technological advances and the expansion of the production scale, these costs may gradually decrease [35,40,41,42]. In addition, ionic liquid lithium–sulfur batteries may offer better economics in the long run, given their longer cycle life and higher energy density [43,44,45]. Lithium-ion liquid lithium–sulfur batteries have better environmental performance compared with traditional lithium-ion batteries, mainly in the renewability and recyclability of materials, which can also further control costs [46,47]. Furthermore, the solubility of lithium polysulfides (LiPSs) in ionic liquid (IL)-based electrolytes demonstrates a significant dependence on the anionic structure [48]. Asymmetric anions are distinguished by a stable polyfluorinated structure at one terminal and robust solvation with high ionic conductivity at the other terminal. This design preserves the characteristics of symmetric anions while surpassing their limitations, thereby enhancing the performance of lithium–sulfur (Li–S) batteries [49]. Therefore, the adoption of the asymmetric ionic liquids as electrolytes presents a promising strategy for the practical and stable application of lithium–sulfur batteries [35,41,50]. Thus, it is crucial to investigate the diverse binding modes of ionic liquids and their interactions and reaction processes with LiPSs [18,35]. However, experimental illumination of the reaction pathway and structural details is often challenging. In contrast, computational modeling methods offer the potential to clarify the reaction mechanisms and pathways occurring at the electrolyte–LiPSs interface [11]. For example, Hu et al. performed classical molecular dynamics (CMD) simulations to investigate the microscopic mechanisms and transport behaviors of typical Li_2_S_8_ species in ionic liquids and ionic liquid-based electrolyte systems. The dynamic characteristics revealed that the presence of anion [TFSA]^−^ in IL electrolytes promotes faster Li^+^ exchange rates and facilitates the dissociation of Li^+^ solvation structures [51]. Liu et al. employed density functional theory (DFT) calculations to demonstrate that the stable molecular configurations of short-chain lithium polysulfides (Li_2_S_x_, where 1 ≤ x ≤ 3) exhibit a linearly serrated structure. Conversely, the stable molecular structure of long-chain lithium polysulfides (Li_2_S_x_, where 4 ≤ x ≤ 8) tends to be cyclic when dissolved in both (1,3-dioxolane) DOL and (1,2-dimethoxyethane) DME solvents. These findings have significant implications for the anchoring of lithium polysulfides at the negative electrode [52]. Nevertheless, the intricate architectures, binding patterns, and the nature interactions between ionic liquids and lithium polysulfide in ionic liquid-based lithium–sulfur batteries demand urgent exploration.

Recognizing the significance of diverse anion-based ionic liquids in lithium–sulfur batteries, meticulous scrutiny was devoted to four distinct ionic liquids comprising various symmetric anions, namely *cis/trans*-[TFSA]^−^, *cis/trans*-[FSA]^−^, *cis/trans*-[FTFSA]^−^, and *cis/trans*-[MCTFSA]^−^, coupled with the cation 1-butyl-1-methylpyrrolidinium [C_4_mpyr]^+^. Moreover, composite models were constructed to unravel the interactive mechanisms of ionic liquids in lithium–sulfur batteries, individually examining the cation, anion, and the ion-pair interacting with S_8_ and Li_2_S_n_ (where n = 1, 2, 4, 6, 8). Given the propensity of S_8_ to readily react with Li^+^ and form lithium polysulfide intermediates, it is imperative to consider the analysis of S_8_ in Li–S batteries [53,54,55]. To streamline discussions, we shall henceforth refer to both S_8_ and Li_2_S_n_ (where n = 1, 2, 4, 6, 8) collectively as LiPSs. In addition to the energy and structure calculations, an array of analytical techniques, including atoms in molecules (AIM) [56], an independent gradient model based on Hirshfeld partition (IGMH) [57], electron density difference (EDD), charge decomposition analysis (CDA) [58] and symmetry-adapted perturbation theory (SAPT) [59] energy decomposition analysis were employed to further illustrate the nature of the interactions and the electronic properties of these complexes in order to investigate the electron transfer process and its performance of the new ionic liquid lithium–sulfur battery electrolyte, so as to better combine the theory with practice.

## 2. Computational Methods

### 2.1. Theoretical Methods

The structural optimization of the species in this investigation was achieved by employing the M06-2X [60] functional, a hybrid density functional developed by Truhla’s group. The theoretical methodology has been well-established for its reliable description of various types of weak interactions [61,62,63] and widely adopted for examining the theoretical interactions between ion pairs in ionic liquids [64,65,66]. Regarding the selection of the basis set, we employed the aug-cc-pVDZ developed by Dunning [67], known for its precision and applicability in accurately describing molecular properties. The computational systems were all investigated using zero-damping dispersion-corrected density functional theory [DFT-D3(zero)] [68]. The corresponding structure was analyzed at the same theoretical level to ensure that all the structures were at the lowest point of the local potential energy surface. The calculations were carried out with the Gaussian 09 suite of programs [69]. Analysis was undertaken with the Multiwfn program [70] and visualized using the VMD package [71].

### 2.2. Study of the Dimeric Interactions

**Figure 1:**
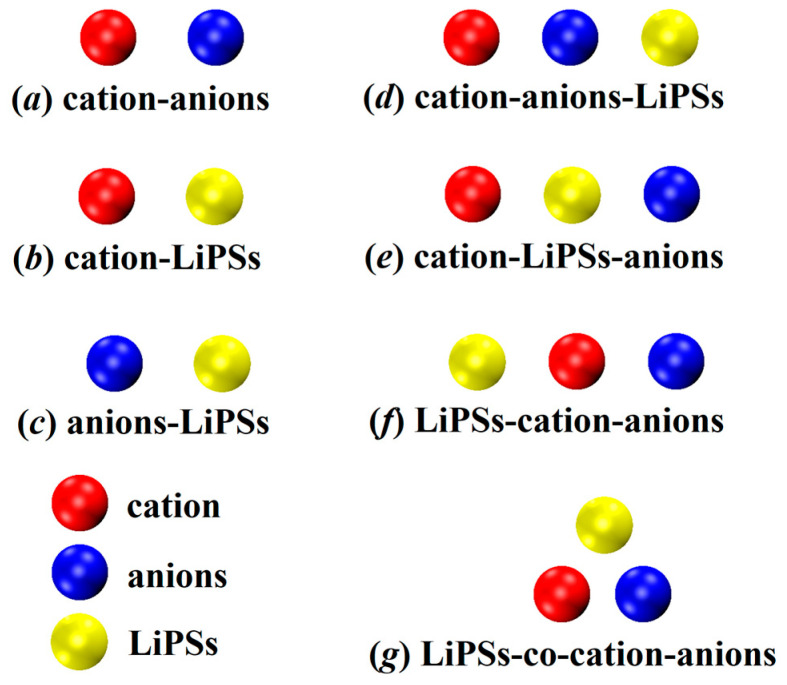
Schematic representation of the complex binding mode of the dimer (**a**–**c**); and trimer (**d**–**g**) involved in this study.

To explore the individual interactions among different components in the lithium–sulfur battery system, we initially created and optimized three pairs of models for a total of 62 configurations. As depicted in Figure 1a–c, (a) 8 models represent the combination of anions and cations within the ionic liquid; (b) 6 models consist of cations and lithium polysulfides in a complex arrangement; and (c) 48 models involve anions and lithium polysulfides.

### 2.3. Study of the Trimeric Interactions

To delve deeper into the intricacies of these more realistic systems, we studied the synergistic mechanism between ionic liquids and lithium polysulfides. Based on the distinctive orientations of their combinations, we devised a trimeric model comprising 192 configurations categorized into four distinct groups. The schematic diagram depicting these categories is illustrated in Figure 1d–g. In configuration (d), the cation and anion form an ionic liquid, with lithium polysulfides intimately bound to the anion. The anion serves as the pivotal element, securely anchoring the three constituents together. Moving on to configuration (e), the cation and lithium polysulfides are not in direct contact, while the polysulfide acts as the direct adhesive substance between the three entities, intimately binding the polysulfide to the anode ion. Configuration (f) mirrors the arrangement observed in (d), wherein the cation and anion first combine to form an ionic liquid. However, in this case, lithium polysulfides are firmly attached to the cation, with the cations serving as the adhesive material for the trimeric assembly. Lastly, in configuration (g), the cation and anion initially form an ionic liquid, while lithium polysulfides establish contact with both the cations and anions. This configuration showcases a synergistic effect among the three constituents.

## 3. Results and Discussion

### 3.1. Structures and Energetics of the Complexes Containing Different Interactions

**Figure 2:**
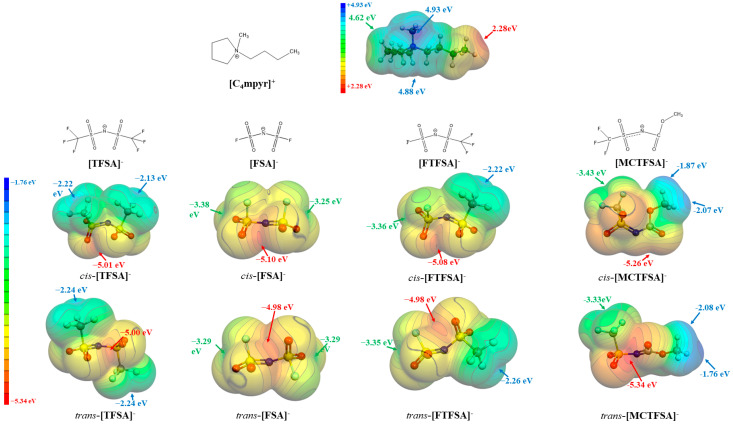
The ESP surfaces (isosurface values at 0.001 a.u.) of isolated cation and anions, together with V_s,max_ and V_s,min_.

Driven by the recognition that electrostatic forces predominantly govern the interactions between cations and anions in ionic liquids (ILs), we conducted an analysis of the molecular electrostatic potential (ESP) [72]. This analysis aimed to determine the energetically stable configurations of ionic pairs, with a specific focus on the isolated forms of the cation and anions. Figure 2 presents the molecular electrostatic potential (ESP) surfaces of the [C_4_mpyr]^+^ cation and four anions (FTFSA^−^, MCTFSA^−^, TFSA^−^, and FSA^−^), highlighting the locations of local maxima and minima, denoted as V_s,max_ and V_s,min_, respectively.

It is evident that the [C_4_mpyr]^+^ cation exhibits a pronounced positive electrostatic potential, reaching a maximum value of +4.93 eV in close proximity to the methyl group’s terminus. In the case of symmetric anions, the electrostatic potential minimum is distributed at both ends of the -CF_3_ or -S-F groups. Notably, the latter displays a more negative electrostatic potential (−2.13 eV as compared to −3.38 eV), owing to the heightened electron-withdrawing capability of the sulfur atoms in contrast to the carbon atoms. Asymmetric anions, meanwhile, exhibit an uneven distribution of electrostatic potential. Specifically, the -S-F end of [FTFSA]^−^ demonstrates a more negative electrostatic potential than its -CF_3_ end, while the -CF_3_ end of [MCTFSA]^−^ manifests a more negative electrostatic potential than its -CH_3_ end. These observations suggest that the hydrogen bonds formed by H may possess a weaker bond strength compared to the hydrogen bonds established by F. Among the four anions, the simultaneous presence of *cis/trans*-[MCTFSA]^−^ demonstrates the most pronounced negative electrostatic potential, with values of −5.26 eV and −5.34 eV, respectively. 

**Figure 3:**
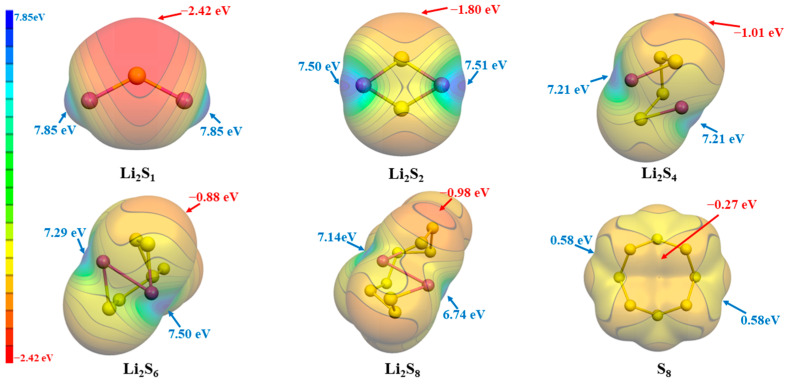
The ESP surfaces (isosurface values at 0.001 a.u.) of Li_2_S_n_ (n = 1, 2, 4, 6, 8) and S_8_, together with V_s,max_ and V_s,min_.

Simultaneously, in order to further investigate the synergy between ILs and LiPSs, we also performed an ESP analysis on the LiPSs, as depicted in Figure 3. In the case of lithium polysulfides (LiPSs), the analysis of electrostatic potential indicates that the positive electrostatic potential consistently resides in close proximity to the Li atoms. Conversely, the negative electrostatic potential near the S atoms intensifies as the number of S atoms increases, attributed to electron dispersion. Conversely, the electrostatic potential in the proximity of the lithium atoms exhibits a gradual reduction as the quantity of sulfur atoms grows, with the exception of a momentary escalation observed in Li_2_S_6_.

**Figure 4:**
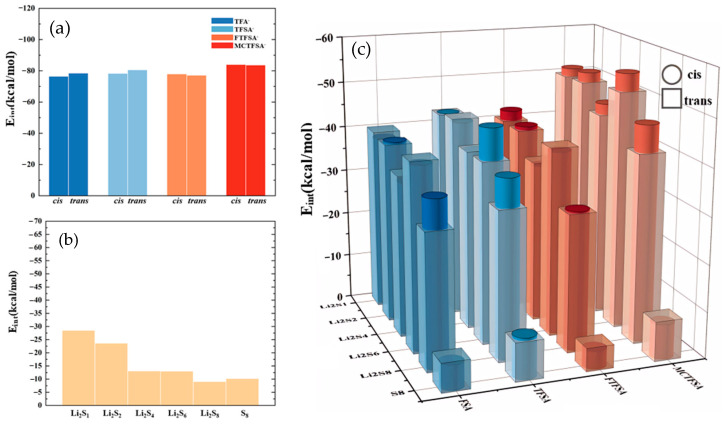
(**a**) The interaction energies between cation and anion within ionic liquids; (**b**) the interaction energies between cation and lithium polysulfide; and (**c**) the interaction energies between anions and lithium polysulfides.

To elucidate the structural characteristics of different complexes, we constructed and optimized various models using the ESP results as a foundation. The interaction energies of the optimized dimeric structures are presented in Figure 4, providing the energetic landscapes of these complexes. The interaction energy (*E*_int_) is defined as:

(1) $$E_{\mathrm{int}}=E_{\mathrm{system}}-\sum_{i}E_{i}+E_{\mathrm{BSSE}}$$

The total energy of system, denoted as *E*_system_, and the optimized energy of the isolated monomer (whether cationic, anionic, or lithium polysulfides), referred to as *E*_i_, along with the energy of basis set superposition error (*E*_BSSE_) [73,74,75,76,77]. Notably, in examining the ionic liquid interaction energy in Figure 4a, it becomes evident that the most pronounced interaction energy was observed between [MCTFSA]^−^ and the cation [C_4_mpyr]^+^, with values of −83.94 kcal/mol for the *cis*-configuration and −83.67 kcal/mol for the *trans*-configuration. Conversely, the other three anions exhibited comparatively fewer disparities, hovering around −80 kcal/mol. This disparity signifies the preferential and more stable binding of the [MCTFSA]^−^ anion to the cation. Attributed to the ESP analysis, [MCTFSA]^−^ has a lower electrostatic potential, forcing it to bind preferentially to cations [65,66]. Furthermore, the symmetric anions [FSA]^−^ and [TFSA]^−^ exhibited higher interaction energies in the *trans*-structural configuration when interacting with the cations, registering at −78.46 kcal/mol and −80.54 kcal/mol, respectively. In contrast, these values decreased in the cis configuration, measuring at −76.47 kcal/mol and −78.26 kcal/mol, respectively. On the other hand, the asymmetric anions [FTFSA]^−^ and [MCTFSA]^−^ exhibited a contrasting trend. In the trans configuration, their interaction energies were −77.87 kcal/mol and −83.94 kcal/mol, respectively, whereas in the cis configuration, they measured −77.11 kcal/mol and −83.67 kcal/mol, respectively. This can be ascribed to the heightened negativity of the electrostatic potential observed in the electrostatic potential analysis of the [MCTFSA]^−^ anion. This attribute facilitates a greater comprehension of the augmented interaction energy upon its association with the cation. This is attributed to the fact that the relatively iso-side structure of the asymmetric anion promotes strong binding to the cation on one side, whereas the symmetric anion is trans-exposed to a greater number of binding sites. Simultaneously, the non-fluorinated methyl -CH_3_ terminus, in conjunction with the fluorinated -CF_3_ terminus, positioned on the same side within the *cis*-[MCTFSA]^−^ configuration, unveils a greater number of oxygen binding sites. Consequently, these structural attributes culminate in elevated interaction energies in comparison to the *trans*-structural conformation. Notably, this analysis underscores the greater strength of the trans structure in symmetric anions and the prominence of the cis configuration in asymmetric anions. Consequently, it is imperative to consider both isomers in all the calculations presented in this study, despite previous computational works focusing solely on the *trans*- or *cis*-isomer [78,79,80,81].

As depicted in Figure 4b, it is evident that the cation exhibited the highest interaction energy with Li_2_S_1_ (−28.49 kcal/mol), with the interaction energy gradually diminishing as the number of sulfur atoms increased. However, there was a subsequent increase in interaction energy during the interaction with S_8_. This observation indicates that the interaction between the cation and lithium polysulfides weakens as the number of S elements increases, yet the distinctive ring structure of S_8_ enhances its interaction with the cation [82,83,84].

In Figure 4c, it can be observed that, among the various anions interacting with lithium polysulfides, the interaction energy with S_8_ is the lowest in this system. The electrostatic potentials of S_8_ exhibit a near-neutral state, with both positive and negative charges converging towards zero [85]. This balance, along with the unstable nature of its binding mode [53], contributes to a diminished interaction energy. Furthermore, only the cis structure of the [TFSA]^−^ anion exhibits a higher interaction energy compared to the trans structure and lithium polysulfides. Conversely, the other three anions exhibit stronger interaction energies than the cis structure. However, similar to Figure 4a, the strongest interaction energy between the [MCTFSA]^−^ anion and lithium polysulfides is observed. With the exception of the interaction with S_8_, the interaction energy with other lithium polysulfides is higher in the cis configuration. In a comprehensive analysis, the interaction energy between the anion and lithium polysulfides manifests a discernible sequence of descending, ascending, and descending trends from Li_2_S_1_ to Li_2_S_4_, Li_2_S_4_ to Li_2_S_6_, and Li_2_S_6_ to Li_2_S_8_, respectively. Notably, S_8_ demonstrates the least energetically favorable interaction. This observation aligns with the trend of positive electrostatic potential variations surrounding the lithium atom, as evidenced by the analysis of ESP for the LiPSs.

**Figure 5:**
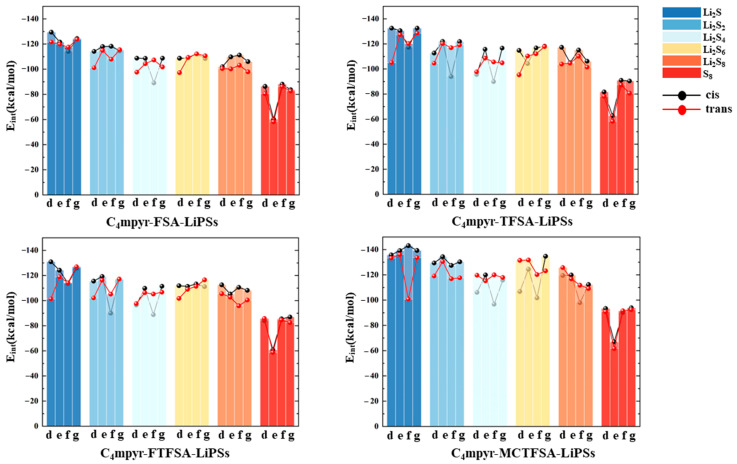
The interaction energies between ionic liquids and lithium polysulfides.

The interaction energies of the trimeric complex are quantified in Figure 5. Examining the distinct lithium polysulfides, a similar interaction pattern to anions and lithium polysulfides is observed. Across the four systems, there’s a progressive decline from Li_2_S_1_ to Li_2_S_2_ to Li_2_S_4_, followed by a slight increase from Li_2_S_4_ to Li_2_S_6_. Subsequently, there is a further decrease from Li_2_S_6_ to Li_2_S_8_, with S_8_ demonstrating the lowest structural interaction energy. It can be expected that the anion may assume a more dominant influence in the interactions. Shifting focus to the various anions, in consonance with the findings depicted in Figure 4, the [MCTFSA]^−^ anion emerges as the most energetically favorable among the four anionic systems. Considering different configurations, the *g* structure (c.f. Figure 1g) manifests the highest level of interaction energy among the four configurations, accounting for one third (16 out of 48 binding modes) of the total negative, cation, and lithium polysulfide interactions. This finding suggests that synergistic configurations involving all three components typically confer heightened stability. This may be attributed to the greater number of binding sites exposed in the *g* structure. In this configuration, the negative electrostatic potential in the anion can bind to both the positive electrostatic potential region of the cation and the positive electrostatic potential region of the lithium polysulfides (LiPSs). Additionally, the neutral LiPSs also offer a negative electrostatic potential region to bind to the cation, thereby forming a more stable “triangular” configuration.

### 3.2. Interaction Analysis

#### 3.2.1. Atoms in Molecules (AIM) Analyses

The quantum theory of atoms in molecules (AIM) provides a quantitative framework for investigating the strength and nature of interactions, utilizing the electron density *ρ*(r) at critical points (CPs) [56]. These CPs are characterized by a gradient of the electron density, ∇*ρ*(r), and are further distinguished by the three eigenvalues of the Hessian matrix. Notably, a bond CP (BCP) frequently emerges between adjacent nuclei, serving as a clear indication of the presence of a chemical bond or noncovalent interaction between them. Building upon the aforementioned findings, a representative selection of systems was analyzed, specifically targeting the identification of BCPs between atoms through AIM analysis. In the molecular image, the interactions between two atoms are labelled with red dots and they are connected with red bond paths. In addition, the bond energy (*E*_B_) calculated from the potential energy density (*V*) at the BCP can be used to describe the strength of each interaction [86] as follows:

(2) $$E_{B}=\frac{1}{2}V$$

**Figure 6:**
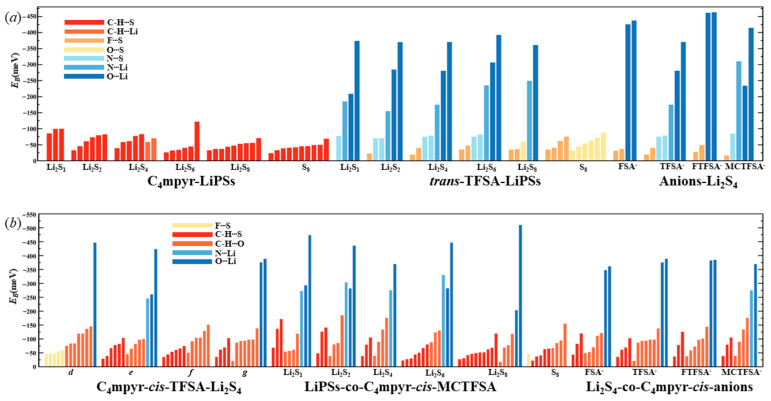
Bond energies for representative geometries: (**a**) dimer and (**b**) trimer.

In order to facilitate the subsequent analyses, representative systems were selected; and the bond energies are plotted as Figure 6, while all the specific structural and topological parameters are detailed in Table S1.

**Figure 7:**
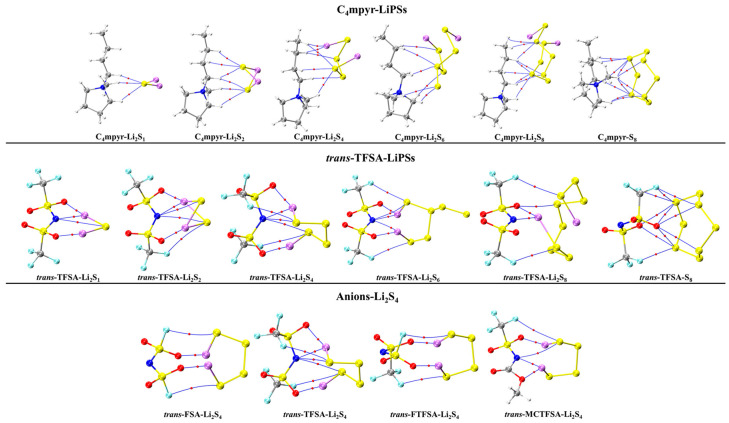
AIM diagrams for representative dimer systems.

The optimized structures of the dimers were analyzed, as depicted in Figure 7. These encompass the interplay between the moderately interacting *trans*-[TFSA]^−^ anion and diverse LiPSs. Furthermore, they encompass the binding interaction between the [C_4_mpyr]^+^ cation and an array of LiPSs, as well as the binding interaction between each of the four distinct anion types and Li_2_S_4_ in the transitional state. In this manner, an exploration of the binding patterns among anions, cations, and lithium polysulfides was undertaken. The analysis of the optimized structures of both the cation and lithium polysulfides revealed a prevalent occurrence of hydrogen bonds, underscoring the affinity of the hydrogen atom in the cation for such interactions with the LiPSs. 

Notably, the combination with S_8_ exhibited the highest number of hydrogen bonds; however, as the number of S atoms increased, the average energy associated with these hydrogen bonds gradually diminished. This observation suggests that the introduction of S elements facilitates the formation of hydrogen bonds while simultaneously dispersing the energy associated with these interactions.

Turning our attention to the binding of *trans*-[TFSA]^−^ and lithium polysulfides, the remarkable strength of the O∙∙∙Li bond energy (−209.4 meV to −374.5 meV) can be attributed to two factors. First, the robust oxidizing properties of the oxygen (O) atoms contribute significantly. Second, the strong negative electrostatic potential near the O atom, as revealed in the electrostatic potential (ESP) analysis, further enhances the bonding strength. In contrast, S_8_ still manifests the highest number of bonds, yet the bond energy is comparatively lower due to the absence of Li. This is in line with the established fact that the binding energy to S_8_ consistently exhibits the lowest values in analyses of interaction energy. Considering the diverse anions in Li_2_S_4_ structures, it is noteworthy that, while symmetrical anions exhibit a greater number of bonds, the average bond energy falls short of that observed in the case of the asymmetric anion. The diminished negative electrostatic potential associated with the symmetrical anion imposes limitations on its capacity to form stronger bonds, thereby elucidating its inability to sustain elevated levels of interaction energies. Remarkably, this asymmetric anion engendered stronger O∙∙∙Li bonds with Li atoms in lithium polysulfides, thereby intensifying the overall interaction between them and bolstering the solubility of lithium polysulfide [87].

**Figure 8:**
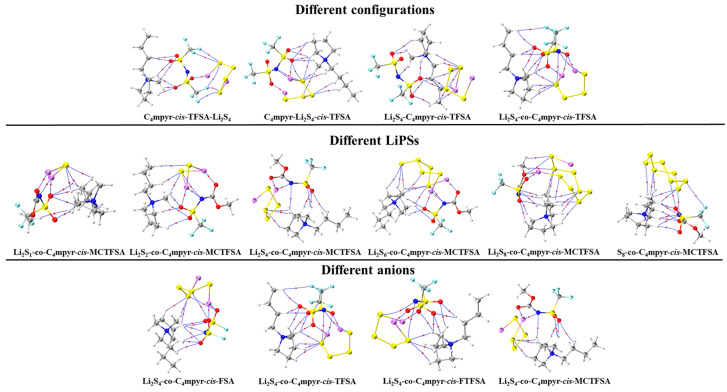
AIM diagrams for representative trimer systems.

Subsequently, a subset of representative trimer structures was carefully chosen and is presented in Figure 8. In order to investigate the impact of various binding modes on the specific structural analysis, four distinct binding modes of C_4_mpyr-*cis*-TFSA-Li_2_S_4_ were initially chosen, encompassing the moderately interacting *cis*-[TFSA]^−^ anion and Li_2_S_4_ in the transition state. Furthermore, the binding of ionic liquids to a range of lithium polysulfides was subsequently evaluated, wherein the *cis*-[MCTFSA]^−^ anion in the stable g configuration exhibited the highest interaction energy.

Similarly, the influence of different anions in the binding process was also examined, focusing on the transition state Li_2_S_4_ and the g configuration. Similar to the dimers, the trimers also exhibited a prevalence of hydrogen bonds, albeit with a noteworthy emphasis on the substantial O∙∙∙Li bond energy. The observation reveals that the g configuration demonstrates a higher degree of interactivity compared to alternative configurations. Notably, a triad of fragments within the g configuration engages in a synergistic interplay, contributing to the elucidation of the relatively superior stability characteristic of the g configuration. In the case of short-chain lithium sulfide compounds, such as Li_2_S_1_and Li_2_S_2_, a tendency towards optimizing the three-fold synergy configuration emerged, with Li atoms directed towards the anion and S atoms oriented towards the cation. This arises from the heightened positive electrostatic potential exerted by short-chain LiPSs in close proximity to lithium atoms, and the corresponding heightened negative electrostatic potential in the vicinity of sulfur atoms. As a consequence, there is an innate propensity for these entities to migrate towards locales harboring a greater abundance of sites conducive to the binding of both anions and cations.

In contrast, long-chain lithium polysulfides, particularly the S_8_ compound, displayed a greater inclination to maintain the initial state after optimization. As an intermediate state, Li_2_S_4_ exhibited a propensity for transitioning towards the *g* configuration after optimization. In the process of binding diverse lithium polysulfides, an augmentation in the count of sulfur atoms unfolds, thereby unveiling a greater expanse of binding sites. This expansion engenders a heightened interaction domain, fostering an amplified formation of C-H∙∙∙S bonds. Nevertheless, the concurrent escalation in the quantity of sulfur atoms has a concomitant effect of attenuating the positive and negative electrostatic potentials, ultimately precluding a commensurate elevation in bond energy levels. The symmetric anionic system exhibits a slightly greater abundance of bonds and higher bonding energy in contrast to its asymmetric counterpart. This disparity can be attributed to the fact that the symmetric anionic fraction offers a more uniform negative electrostatic potential distribution area to the fluorine-containing termini on both ends.

#### 3.2.2. Independent Gradient Model Based on Hirshfeld Partition (IGMH) Analyses

In the year 2017, Lefebvre et al. introduced an electron-based density gradient (∇*ρ*) visual model method known as the independent gradient model (IGM) [88]. This approach enables users to define different fragments within a given system and discern the interactions occurring between these defined fragments. To further enhance the accuracy and precision of the IGM method, an advanced variant, referred to as the independent gradient model based on Hirshfeld partition (IGMH), was subsequently proposed. In this refined approach, the free-state atomic densities utilized in the IGM technique are substituted with atomic densities derived through the Hirshfeld partitioning of the actual molecular electron density [57]. 

**Figure 9:**
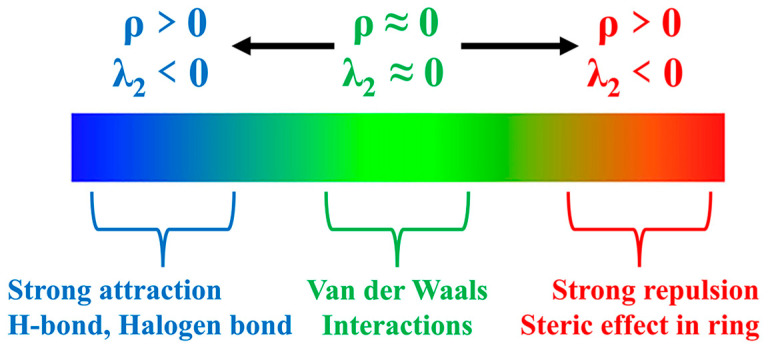
RGB standard fills different weak interaction isosurfaces.

Figure 9 presents a map illustrating the weak interactions among representative dimers with an isosurface value of 0.005 a.u. The isosurface is visually represented using the RGB standard, where the color blue signifies strong attraction, green represents weak van der Waals interactions, and red denotes repulsion. This visualization reveals a multitude of interactions, exhibiting varying strengths, between lithium polysulfides and both cations and anions. Additionally, a two-dimensional scatter plot illustrating δg^inter^ versus sign(λ_2_)*ρ* in the corresponding system is included. Notably, the negative region of the scatter map demonstrates an attractive effect, while a spike in the area exhibiting positive values indicates the presence of a repulsive effect attributed to hindrance within the ring structure.

**Figure 10:**
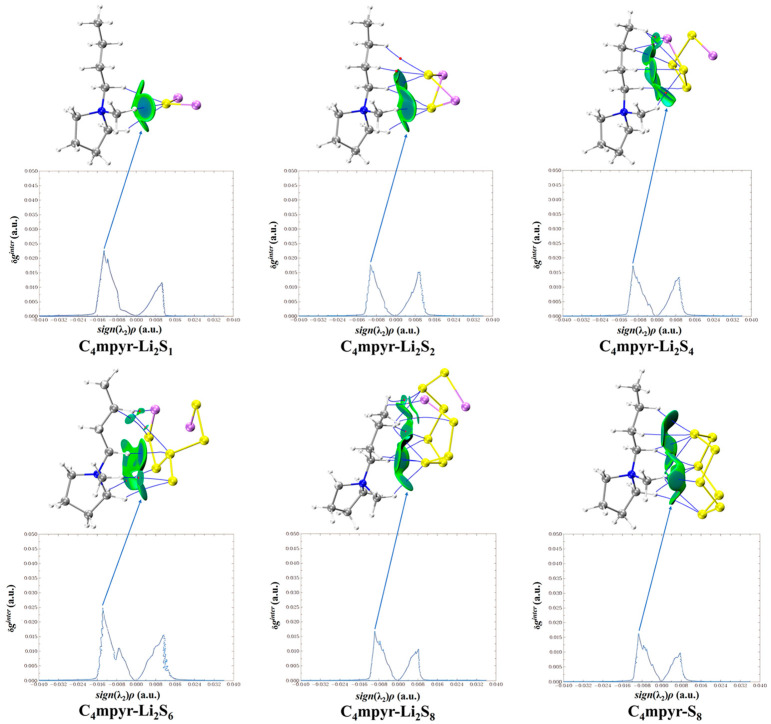
Cation weak interaction diagram with lithium polysulfide.

Upon examination of Figure 10, it becomes evident that cations and lithium polysulfides exhibit relatively weak interactions, as indicated by their proximity to the cyan color. Consistent with the preceding AIM analysis of the structure, it is observed that the interaction region expands proportionally with the augmentation in the quantity of sulfur (S) atoms.

**Figure 11:**
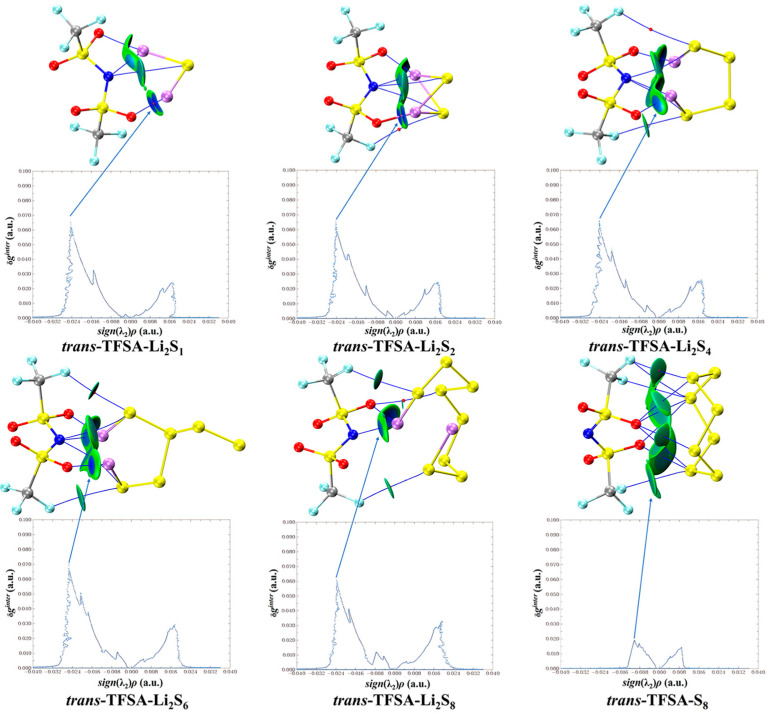
*trans*-[TFSA]^−^ anion weak interaction diagram with lithium polysulfide.

Similarly, when considering the interaction between *trans*-[TFSA]^−^ anions and different lithium polysulfides in Figure 11, it becomes apparent that, consistent with the pattern of fluctuations during interactions, the binding to S_8_ is the weakest, whereas the Li atoms in other lithium polysulfides form a stronger attraction with the O atoms.

**Figure 12:**
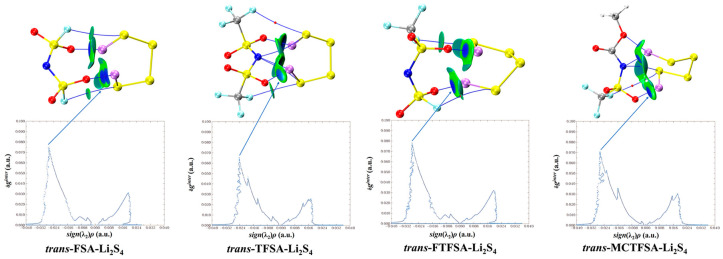
Weak interaction diagram of different anions with Li_2_S_4_.

Furthermore, it should be noted that, in Figure 12, the asymmetrical anions display a higher degree of interaction strength, with the [FTFSA]^−^ anion showing the highest affinity.

**Figure 13:**
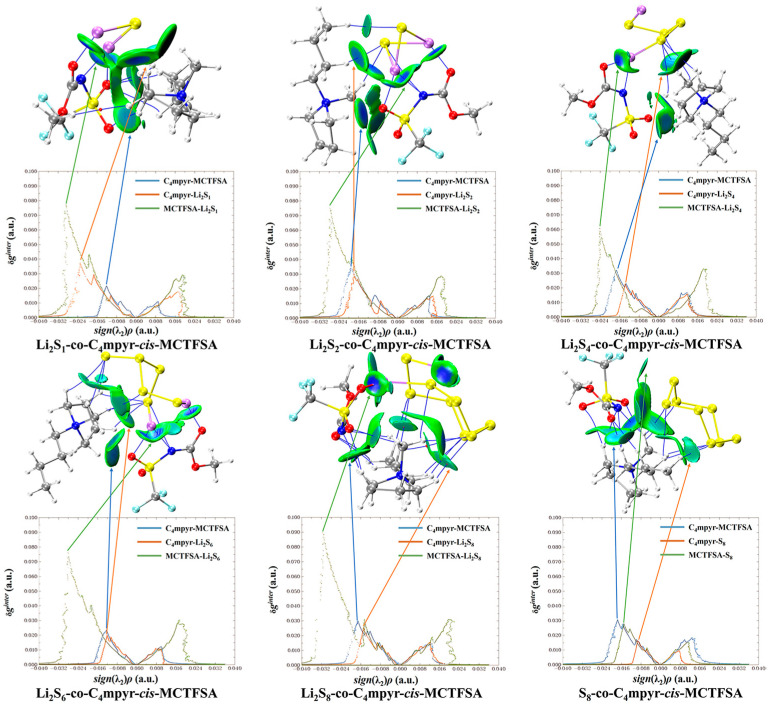
Weak interaction diagram of *cis*-[MCTFSA]^−^ binding to form an ionic liquid with lithium polysulfide.

According to the IGMH analysis of the trimer depicted in Figure 13, it can be observed that the anions form the most stable interaction with lithium polysulfides, with the exception of the structure that is bound to S_8_. This stability can be attributed to the remarkable electron-gain capability exhibited by the oxygen atoms in the anion, coupled with the robust electron-loss propensity demonstrated by the lithium atoms in lithium polysulfides.

**Figure 14:**
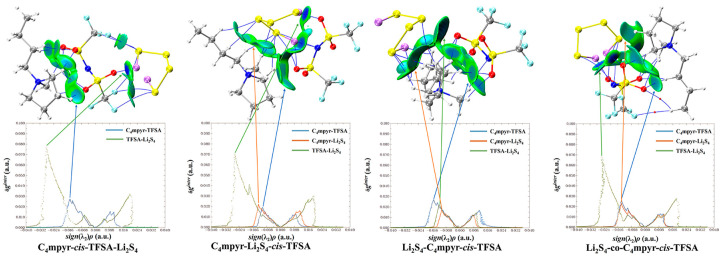
Weak interaction diagrams of different configurations of *cis*-[TFSA]^−^ binding forming ionic liquids with lithium polysulfide.

Subsequent investigations revealed a correlation between the physical phase of lithium polysulfide and the prevalence of cations and anions within the system, shown in Figure 14. In liquid-state long-chain lithium polysulfides, such as Li_2_S_6_ and Li_2_S_8_, as well as in the solid-state S_8_ system, it is evident that the interactions between anions and lithium polysulfides far surpass the interactions between cations and lithium polysulfides, as well as the interactions within anionic and cationic liquids. This observation underscores the dominant role played by anions during this phase.

**Figure 15:**
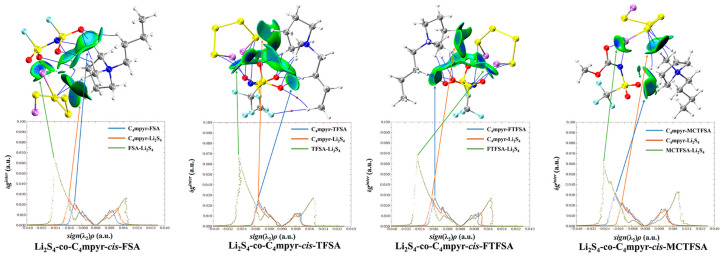
Weak interaction diagram of different anions binding to form ionic liquids with lithium polysulfide.

However, during the transition from the liquid state of Li_2_S_4_ to the insoluble short-chain lithium polysulfides (Li_2_S_1_ and Li_2_S_2_), an intriguing shift occurs (Figure 15). The interaction between cations and lithium polysulfides gradually intensifies over this transition period. Eventually, in the Li_2_S_1_ system, it exceeds the interaction between anions and cations within ionic liquids (ILs). Although not as strong as the interaction between anions and lithium polysulfides, this finding highlights the significant influence of cations that cannot be disregarded during this stage.

It is noteworthy that there is no significant discrepancy between the symmetric and asymmetric anions. However, [MCTFSA]^−^ exhibits a more pronounced ionic–liquid interaction, indicative of its heightened affinity in this context.

### 3.3. Electron Transfer Analysis

#### 3.3.1. Electron Density Difference (EDD) Analyses

The representative system electron density difference (EDD) analysis structure is shown in Figure 11 and is defined as follows:

(3) $$\Delta \rho =\rho_{\mathrm{system}}-\sum_{i}\rho_{i}$$

where *ρ*_system_ denotes the electron density of the entirety of the system, *ρ_i_* refers to the electron density pertaining to the distinct fragment *i*, with the coordinates mirroring those within the complex. Through the depiction of the *ρ* function via isosurfaces in three dimensions, and the subsequent filling of isosurfaces with varying types to differentiate positive and negative values, we are bestowed with the means to discern the increase or decrease in electron density among the fragments. Consequently, this framework allows for the tendency of electron migration between the complexes to be determined. 

**Figure 16:**
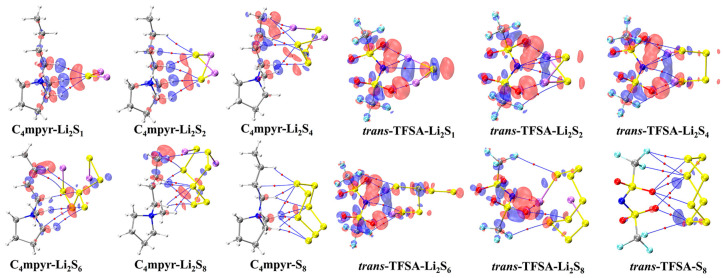
Plot of electron density differences of representative dimers. The red and blue regions represent increased electron density and decreased electron density, respectively.

In the surface EDD mapping of the cation and LiPSs dimers, a noteworthy redistribution of electron density is observed along the hydrogen-bond axis (Figure 16). Specifically, the electron density increases in the area of the hydrogen bond acceptor S atoms, while it decreases in the region of the hydrogen bond donor H atoms. This suggests a migration of electrons from the Li atoms to the S atoms within the lithium polysulfides, as well as from the H atoms to the C atoms within the cation, along the covalent C–H bond. Overall, in the intermolecular region, there is a tendency for electrons to migrate from the lithium polysulfides to the cation. 

In the cases of the anion and lithium polysulfides, the O∙∙∙Li bond emerges as the prominent feature. The internal electrons of the lithium polysulfides undergo migration from the Li atoms to the S atoms, and this electron transfer abates with an increasing number of S atoms. The phenomenon of electron transfer elucidates the inclination towards facile interaction formation as the count of sulfur atoms escalates, providing an augmented number of binding sites. However, concomitantly, there is a reduction in the electron transfer, which consequently restricts the reinforcement of these interactions. However, there is only minimal electron transfer between the cation or anion and S_8_, aligning with the relatively weak strengths illustrated by the IGMH isosurface. This propensity can be attributed to the dominance of dispersive interactions rather than electrostatic interactions in the binding of both anionic and cationic species to S_8_.

**Figure 17:**
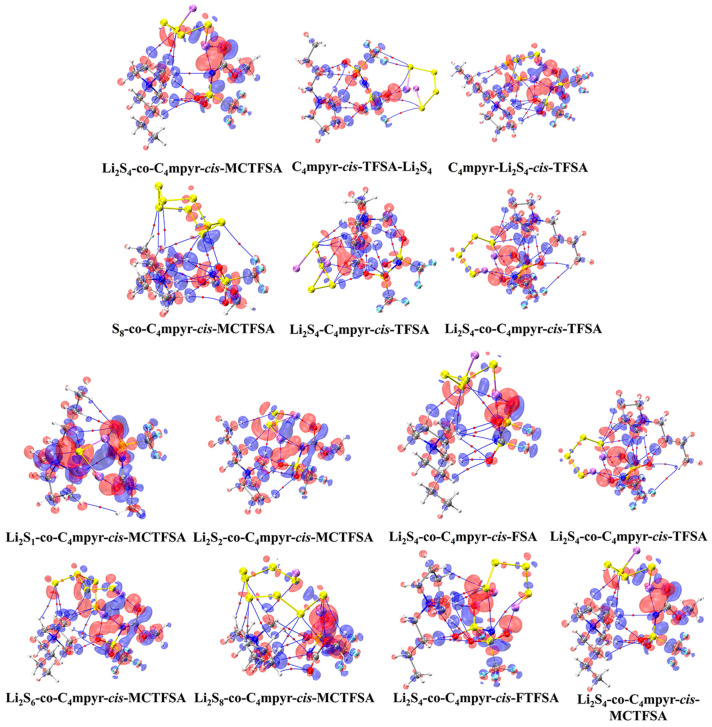
Plot of electron density differences of representative trimers. The red and blue regions represent increased electron density and decreased electron density, respectively.

Furthermore, in the trimer system, consistent with the findings of the IGMH investigation, the anions predominantly carry the charge transfer within the system and serve as the primary electron donors (Figure 17). In their area, coupled with the cation and lithium polysulfide, a considerable increase in electron density is apparent.

#### 3.3.2. Charge Decomposition Analysis (CDA) 

The charge decomposition analysis (CDA) method, proposed by Dapprich and Frenking in 1995 [58], uses the concept of a segment orbit to decompose the electron transfer between different segments, which defines three quantities, as follows:

(4) $$d_{i}=\sum_{m\in A}^{\mathrm{occ}}\sum_{n\in B}^{\mathrm{vir}}\eta_{i}C_{m,i}C_{n,i}S_{m,n}$$

(5) $$b=\sum_{m\in A}^{\mathrm{vir}}\sum_{n\in B}^{\mathrm{occ}}\eta_{i}C_{m,i}C_{n,i}S_{m,n}$$

(6) $$r_{i}=\sum_{m\in A}^{\mathrm{occ}}\sum_{n\in B}^{\mathrm{vir}}\eta_{i}C_{m,i}C_{n,i}S_{m,n}$$

Refer to Note S1 for specific parameter definitions. The term ‘*d*’ signifies electron transfer from the donor to the acceptor, while ‘*b*’ represents electron feedback from the acceptor to the donor. The electron polarization term ‘*r*’ signifies repulsion or bonding characteristics in the orbital overlap region.

The previously stated definition of CDA exhibits certain constraints. Initially, it is applicable solely to closed-shell systems. Additionally, while complex orbitals can employ natural orbitals with non-integer occupancies, fragment orbitals are unable to do so. This discrepancy arises from the fact that the occupation number of fragment orbitals is neglected, focusing merely on occupation and non-occupation. In order to overcome this limitation, Xiao and Lu introduced an enhanced version of CDA, termed Generalized CDA (GCDA) [89]. In this revised framework, the terms ‘*d*’ and ‘*b*’ are redefined as ‘*t*’:

(7) $$t_{i}=\sum_{m\in A}\sum_{n\in B}\eta_{i}\frac{\left|\eta_{m}^{\mathrm{FO}}-\eta_{n}^{\mathrm{FO}}\right|}{\eta_{\mathrm{ref}}}C_{m,i}C_{n,i}S_{m,n}$$

(8) $$r_{i}=\sum_{m\in A}\sum_{n\in B}2\frac{\min\left(\eta_{m}^{\mathrm{FO}},\eta_{n}^{\mathrm{FO}}\right)}{\eta_{\mathrm{ref}}}\eta_{i}C_{m,i}C_{n,i}S_{m,n}$$

**Figure 18:**
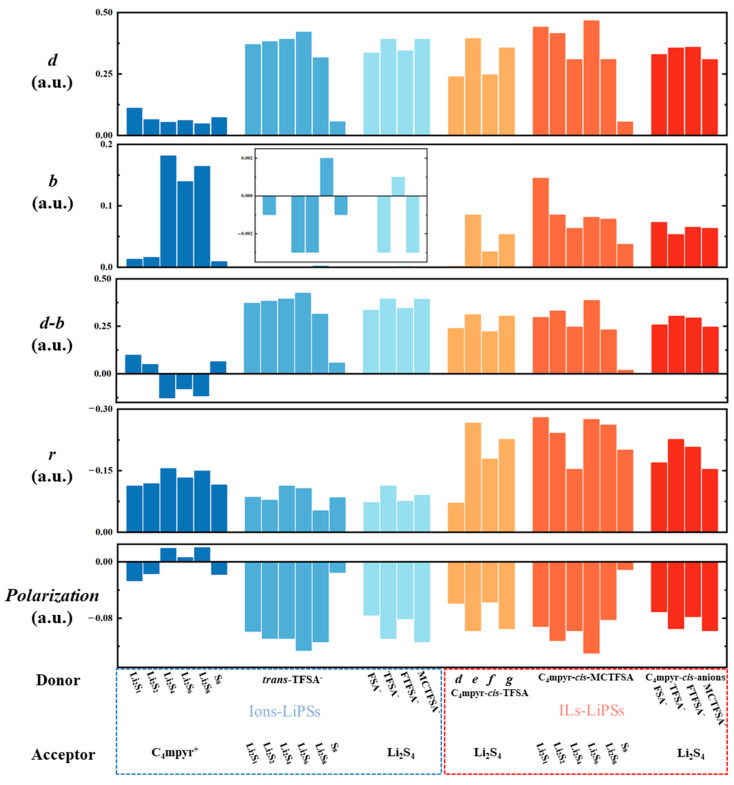
The calculated results of CDA for selected complexes.

The symbol ‘*d-b*’ signifies the difference between the total number of transferred electrons and the total number of feedback-transferred electrons. By employing the Extended CDA (ECDA) [90], one can derive the net number of electron transfers from the donor to the acceptor, where the polarization component is defined as the difference between ‘*d*-*b*’ and the net transfer number. Figure 12 presents the results of CDA and ECDA calculations for the representative system. The results demonstrate that the ‘*d*-*b*’ term of the anion and lithium polysulfides in the dimer exceeds the corresponding value for the cation and lithium polysulfides by a factor of more than three, revealing the large electron migration between the anion and lithium polysulfides (Figure 18).

This is also consistent with the apparently greater electron transfer of anions with lithium polysulfide in the EDD analysis. Within the dimer and trimer configurations, *trans*-TFSA-Li_2_S_6_ and Li_2_S_6_-co-C_4_mpyr-*cis*-MCTFSA emerge as being noteworthy, as they exhibit the most substantial ‘*d*’ values, thereby signifying the inherent capacity of Li_2_S_6_ to embrace a significant charge influx. This observation may elucidate the pivotal role of the binding with Li_2_S_6_ in the transition of the interaction. While the majority of systems typically exhibit ‘*b*’ values surpassing zero, a notable exception arises with the anion–lithium polysulfide systems. In these cases, ‘*b*’ values predominantly fall below zero. This observation underscores the prevailing influence of electron polarization (negative) over charge transfer (positive) within the anion–lithium polysulfide system. The cumulative ‘*r_i_*’ values across all complexes demonstrate a negative sum, a phenomenon given the inherently repulsive nature of occupied orbitals. Notably, in most of the configurations, the ‘*d*-*b*’ term tends to assume small values for the cation and lithium polysulfides arrangement.

**Figure 19:**
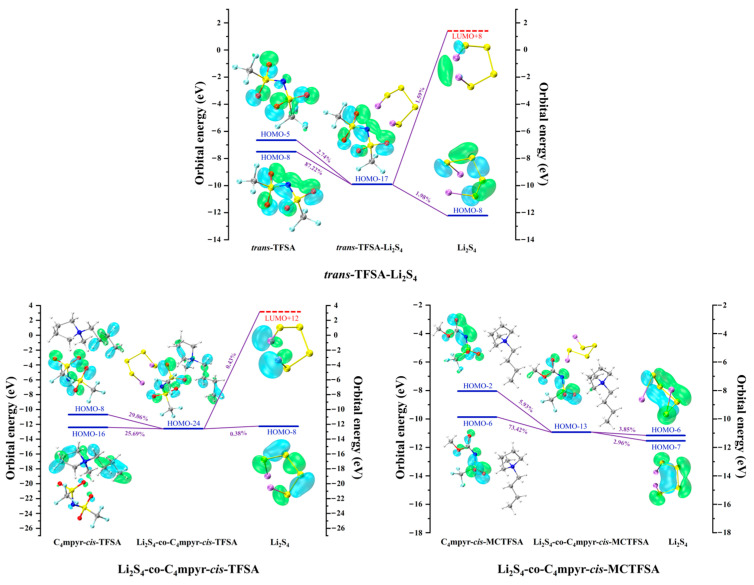
Representative donor–acceptor orbitals of selected complexes.

Figure 19 presents representative molecular orbital maps elucidating the donor and acceptor contributions within the corresponding complexes. Notably, in the dimer *trans*-TFSA-Li_2_S_4_, the key donor orbital is the HOMO-8 orbital, predominantly localized along the N-O bond. However, in Li_2_S_4_-co-C_4_mpyr-*cis*-TFSA, where ionic liquids are formed, the orbital of the anion undertakes a remarkable dispersion, rendering its relative contribution to the entire complex, as opposed to being confined to a singular orbital. Remarkably, HOMO-8 (29.06%) primarily resides on the anions, whereas HOMO-16 (25.69%) predominantly resides on the cations. 

This observation emphasizes the pronounced influence of ionic liquid formation on orbital interactions and charge transfer. Interestingly, within the trimer Li_2_S_4_-co-C_4_mpyr-*cis*-MCTFSA, the most significant donor orbital emerges as the HOMO-6 orbital (73.42%), primarily distributed across the anions. Furthermore, the asymmetric anion [MCTFSA]^−^ exhibits more robust orbital interaction and charge transfer than its symmetric counterpart, the anion [TFSA]^−^.

### 3.4. Energy Decomposition Analysis

The widely recognized Symmetry-Adapted Perturbation Theory (SAPT) serves as a well-established approach for determining intermolecular interaction energies, encompassing physical effects including electrostatics, induction (polarization), dispersion, and exchange [59,91,92,93]. The total interaction energy (Δ*E*_int_) derived from the SAPT2+ method can be dissected into the following four components:

(9) $$\Delta E_{\mathrm{int}}=E_{\mathrm{es}}+E_{\mathrm{ex}}+E_{\mathrm{ind}}+E_{\mathrm{disp}}$$

*E*_es_, a descriptor of the electrostatic component, characterizes the Coulombic forces exerted by static electricity. *E*_ind_ represents the amalgamation of charge transfer and orbital interactions, denoting the induced energy. *E*_disp_ epitomizes the dispersion energy within the system. *E*_ex_ embodies the repulsive exchange energy arising from the Pauli exclusion principle.

**Figure 20:**
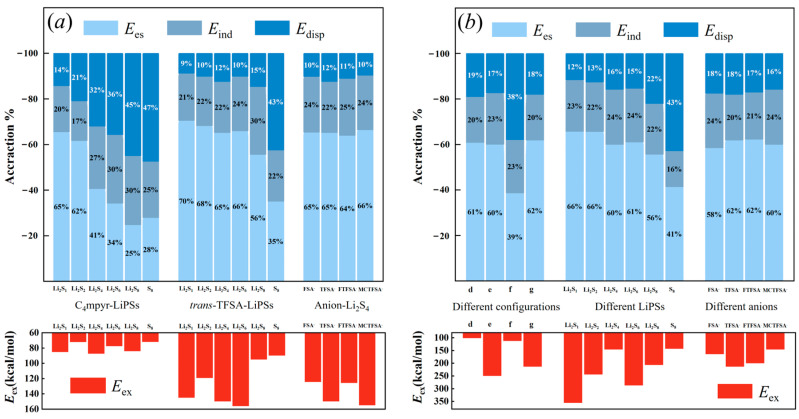
The energy contributions for: (**a**) dimer; and (**b**) trimer systems.

The energy decomposition analysis depicted in Figure 20 unveils a discernible trend in the binding of cations and lithium polysulfides. As the number of sulfur atoms increases, there is a clear evolution in the contributions of different energy components. Specifically, the contribution of electrostatic energy gradually diminishes, while the contribution of dispersion energy exhibits a gradual increase.

Conversely, in the case of anions combined with lithium polysulfides, the electrostatic energy consistently remains the most dominant contributor, wherein S_8_ exhibits the utmost dispersion energy. This observation implies that the solid, insoluble S_8_ predominantly engages in dispersive interactions. However, upon its amalgamation with Li^+^ ions, the dispersive energy swiftly diminishes, giving way to a more dominant electrostatic energy that fosters the formation of soluble Li_2_S_8_. This inherent instability of S_8_ can be attributed to the rapid transition between these energy states. Additionally, it is noteworthy that the anion exhibits significantly higher exchange exclusion energy compared to the cation. This disparity may be attributed to the shorter O∙∙∙Li bond length of the anion, which produces a heightened steric exclusion effect. The absence of the Li atom in S8 further corroborates this hypothesis, as its exclusion energy is relatively low. 

In the trimer structures, a substantial majority of the systems exhibit the highest contribution from electrostatic energy (approximately 60%). This finding aligns with the substantial charge transfer observed in the analysis of these systems using EDD analysis, as supported by the heightened ‘*d*-*b*’ values derived from the CDA data. Similar to the dimer systems, the asymmetric anionic system also showcases a greater electrostatic energy contribution. While the binding to S_8_ exhibits a slightly higher contribution from dispersion energy compared to electrostatic energy, the configuration of LiPSs–cation–anion requires a greater amount of energy from the dispersion component. This necessity arises due to the distantly bound mode of the anion to lithium polysulfides. As the number of sulfur atoms increases in the trimer, the exchange system associated with different binding configurations of lithium polysulfides aligns with the interaction energy (refer to Figure 5).

### 3.5. Comparison with the Experimental Findings

The solid-state structure of Na [MCTFSA] was discerned by Matsumoto K through the utilization of single-crystal X-ray diffraction. A comparative analysis was then drawn against the X-ray structure of Na [TFSA] [94]. Pertaining to the S-O and S-C bond lengths, they exhibit marginal deviations from those observed in Na [TFSA]. Conversely, the N-S bond in [MCTFSA]^−^ displays a slight contraction, possibly attributable to a reduced extent of charge dispersion [95]. This observation is in line with the discovery that *trans*-[TFSA]^−^ (−75.4 meV) and *trans*-[MCTFSA]^−^ (−85.3 meV) exhibit N-S bond energies when combined with Li_2_S_4_, as indicated by the AIM analysis. However, the EDA reveals a slightly diminished dispersive energy (a reduction of 2%) for *trans*-[MCTFSA]^−^.

The temperature-dependent ionic conductivity of a 1 m Na [MCTFSA] solution in EC:PC (1:1 *v*/*v*) was estimated by Kaitlyn et al. [17], and its results were compared with the findings reported by Ponrouch et al. [96] for the corresponding Na [TFSA] salt dissolved in the same solvent. The experimental data manifest that, across the range of temperatures examined, the conductivities of the Na [MCTFSA] salt solution consistently lag behind those recorded for the [TFSA]^−^ salt. For instance, at 25 °C, the former registers a value of 1.8 mScm^−1^ versus the latter’s 7 mScm^−1^. By replacing a strongly electron-withdrawing trifluoromethansulfonyl moiety in the [TFSA]^−^ anion with a methyl carbonate group, the degree of negative charge delocalization is diminished, thereby augmenting the prevalence of electrostatic interactions between the cation and anion. These findings align with energetic data demonstrating that the [MCTFSA]^−^ anion exhibits the most robust interaction with the [C_4_mpyr]^+^ cation, with calculated energies of −83.9 kcal/mol for the cis configuration and −83.7 kcal/mol for the trans configuration.

## 4. Conclusions

In this study, we employed the M06-2X hybrid functional to explore the impact of anion species on the interplay between ionic liquids and polysulfide, as well as their underlying mechanisms in lithium–sulfur batteries. The ionic liquid consisted of a prototypical cation [C_4_mpyr]^+^, accompanied by four distinct configurations of class-eight anions: *cis/trans*-[TFSA]^−^, *cis/trans*-[FSA]^−^, *cis/trans*-[FTFSA]^−^, *cis/trans*-[MCTFSA]^−^. To gain a qualitative, quantitative, and visual comprehension of the intricate nature and behavior of these interactions, we employed an array of analytical techniques, including ESP, AIM, IGMH, EDD, CDA and EDA analyses.

The investigation revealed that the [MCTFSA]^−^ anion exhibited robust interactions, both when bound to lithium polysulfide and as an ionic liquid. Notably, its heightened electrostatic interactions with Li atoms in lithium polysulfide effectively enhanced the solubility of lithium polysulfide within the electrolyte. Furthermore, the asymmetric anions formed fewer, yet more sturdy, hydrogen bonds to foster a stronger overall interaction. Asymmetric anions, [MCTFSA]^−^, displayed enhanced orbital interactions and charge transfer compared to their symmetric counterparts.

In the systems of ionic liquid and lithium polysulfides with solid S_8_, leading to the formation of long-chain liquid Li_2_S_n_ (4 ≤ n ≤ 8), interesting observations emerged. The interaction strength increased during the transition until Li_2_S_6_, affirming that the existence of Li_2_S_4_ was unstable compared to other long-chain lithium polysulfides, with a greater inclination towards solid short-chain Li_2_S_2_ and Li_2_S_1_ transitions. This observation aligns with the overall trend observed in anion–lithium polysulfide binding interactions within the dimer. It is evident that the anion plays a pivotal role, as supported by its prominent involvement in the IGMH analysis and its dominant contribution to electron transfer according to the EDD analysis. Nevertheless, the hydrogen bonding facilitated by the cation emerges as a significant component of the interaction as the system transitions to the insoluble short-chain lithium polysulfides. This is corroborated by the sustained increase in this interaction in the IGMH analysis, as well as the positive d-b values in the cation binding to the short-chain lithium polysulfide in the CDA analysis.

Concurrently, there is a transfer of electrons from the anions to the lithium polysulfide and from the lithium polysulfide to the cations. Moreover, electrons are also transferred directly from the anions to the cations, with the anions playing a crucial role as the primary electron donors, facilitating charge transfer within the system. Additionally, it is noteworthy that Li_2_S_6_ possesses the capacity to accommodate a substantial influx of charge. The advantages of the asymmetric anion [MCTFSA]^−^, showing higher electrostatic interactions and more stability compared to the symmetric anion in this theoretical study, were also verified in specific experiments.

This study confirms the potential of asymmetric anion [MCTFSA]^−^ to reduce the solubility of lithium polysulfide with higher stability. The selectivity of different physical phases of lithium polysulfide to anions and cations during the interaction with ionic liquids was also determined, which provides theoretical guidance for the design of new ionic liquid electrolytes and the improvement of ionic liquid performance. Based on our theoretical study, ionic liquids are expected to improve the safe cycling of lithium–sulfur batteries.

We hope that the outcomes reported within this study will serve as a reference when probing the potential of ionic liquids to ameliorate the quandaries that beset the domain of lithium–sulfur batteries.

## Data Availability

Data are contained within the article and Supplementary Materials.

## Supplementary Materials

The following supporting information can be downloaded at: https://www.mdpi.com/article/10.3390/ma17112689/s1, Figure S1. Anionic and cationic composition of ionic liquids AIM molecular graphs. Figure S2. Cations with different lithium polysulfide AIM molecular graphs. Figure S3. [TFSA]^−^ anions with different lithium polysulfide AIM molecular graphs. Figure S4. [FSA]^−^ anions with different lithium polysulfide AIM molecular graphs. Figure S5. [FTFSA]^−^ anions with different lithium polysulfide AIM molecular graphs. Figure S6. [MCTFSA]^−^ anions with different lithium polysulfide AIM molecular graphs. Figure S7. Different configurations of [TFSA]^−^ anionic liquid and Li_2_S_1_ AIM molecular graphs. Figure S8. Different configurations of [TFSA]^−^ anionic liquid and Li_2_S_2_ AIM molecular graphs. Figure S9. Different configurations of [TFSA]^−^ anionic liquid and Li_2_S_4_ AIM molecular graphs. Figure S10. Different configurations of [TFSA]^−^ anionic liquid and Li_2_S_6_ AIM molecular graphs. Figure S11. Different configurations of [TFSA]^−^ anionic liquid and Li_2_S_8_ AIM molecular graphs. Figure S12. Different configurations of [TFSA]^−^ anionic liquid and S_8_ AIM molecular graphs. Figure S13. Different configurations of [FSA]^−^ anionic liquid and Li_2_S_1_ AIM molecular graphs. Figure S14. Different configurations of [FSA]^−^ anionic liquid and Li_2_S_2_ AIM molecular graphs. Figure S15. Different configurations of [FSA]^−^ anionic liquid and Li_2_S_4_ AIM molecular graphs. Figure S16. Different configurations of [FSA]^−^ anionic liquid and Li_2_S_6_ AIM molecular graphs. Figure S17. Different configurations of [FSA]^−^ anionic liquid and Li_2_S_8_ AIM molecular graphs. Figure S18. Different configurations of [FSA]^−^ anionic liquid and S_8_ AIM molecular graphs. Figure S19. Different configurations of [FTFSA]^−^ anionic liquid and Li_2_S_1_ AIM molecular graphs. Figure S20. Different configurations of [FTFSA]^−^ anionic liquid and Li_2_S_2_ AIM molecular graphs. Figure S21. Different configurations of [FTFSA]^−^ anionic liquid and Li_2_S_4_ AIM molecular graphs. Figure S22. Different configurations of [FTFSA]^−^ anionic liquid and Li_2_S_6_ AIM molecular graphs. Figure S23. Different configurations of [FTFSA]^−^ anionic liquid and Li_2_S_8_ AIM molecular graphs. Figure S24. Different configurations of [FTFSA]^−^ anionic liquid and S_8_ AIM molecular graphs. Figure S25. Different configurations of [MCTFSA]^−^ anionic liquid and Li_2_S_1_ AIM molecular graphs. Figure S26. Different configurations of [MCTFSA]^−^ anionic liquid and Li_2_S_2_ AIM molecular graphs. Figure S27. Different configurations of [MCTFSA]^−^ anionic liquid and Li_2_S_4_ AIM molecular graphs. Figure S28. Different configurations of [MCTFSA]^−^ anionic liquid and Li_2_S_6_ AIM molecular graphs. Figure S29. Different configurations of [MCTFSA]^−^ anionic liquid and Li_2_S_8_ AIM molecular graphs. Figure S30. Different configurations of [MCTFSA]^−^ anionic liquid and S_8_ AIM molecular graphs. Table S1. The AIM data calculated with M06-2x for all the studied complexes. Note S1. Parameter definitions for formulas in CDA analysis.
