# Supplementary material for: The Interactions between Ionic Liquids and Lithium Polysulfides in Lithium–Sulfur Batteries: A Systematic Density Functional Theory Study
Source: Materials (Basel). 2024 Jun 2;17(11):2689. doi: 10.3390/ma17112689 (PMC11173497; doi:10.3390/ma17112689)
Supplement: Supplementary file 1 [file materials-17-02689-s001.zip › materials-3004804-supplementary.pdf]

The Interactions between Ionic Liquids and Lithium Polysulfides in Lithium-Sulfur Batteries: A Systematic Density Functional Theory Study

Chengren Li,[†,‡] Nan Zhou,[†,‡] Rongde Sun,[†,‡] Jiaxin Tang,[†,‡] Jianglu Liu,[†,‡] Jianhua He,[†,‡] Changjun Peng,[§] Honglai Liu,[§] Shaoze Zhang*,[†,‡]

[†]National Engineering Research Center of Vacuum Metallurgy, Kunming University of Science and Technology, Kunming 650093, Yunnan province, China

[‡]Engineering Laboratory for Advanced Battery and Materials of Yunnan Province, Kunming University of Science and Technology, Kunming 650093, Yunnan province, China

[§]Key Laboratory for Advanced Materials and School of Chemistry & Molecular Engineering, East China University of Science and Technology, Shanghai 200237, China

*Corresponding author: szzhang@kust.edu.cn

Supplemental Information

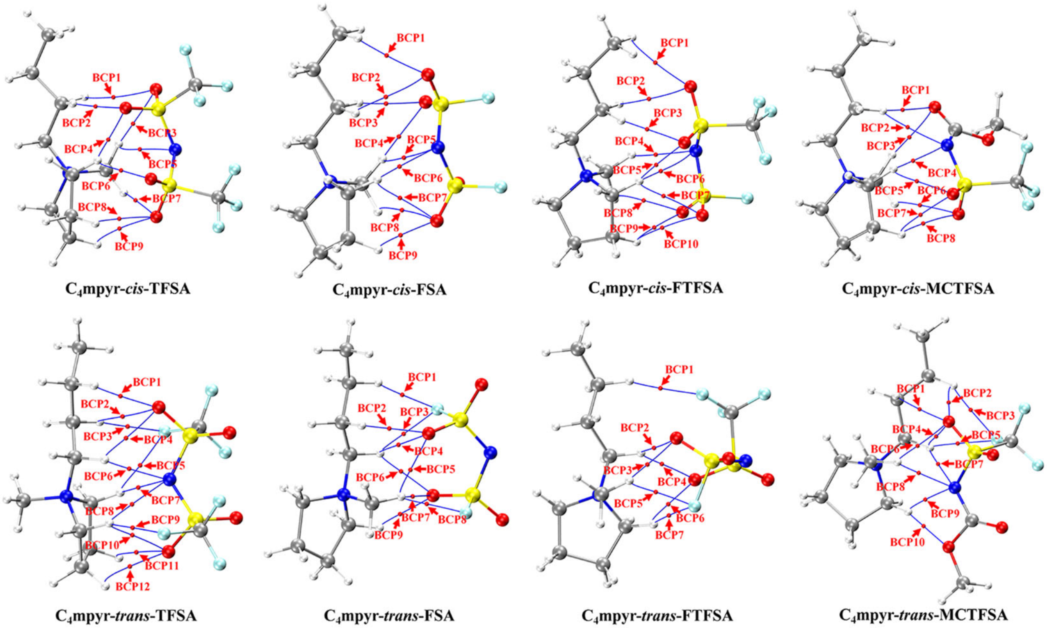

Figure S1. Anionic and cationic composition of ionic liquids AIM molecular graphs.

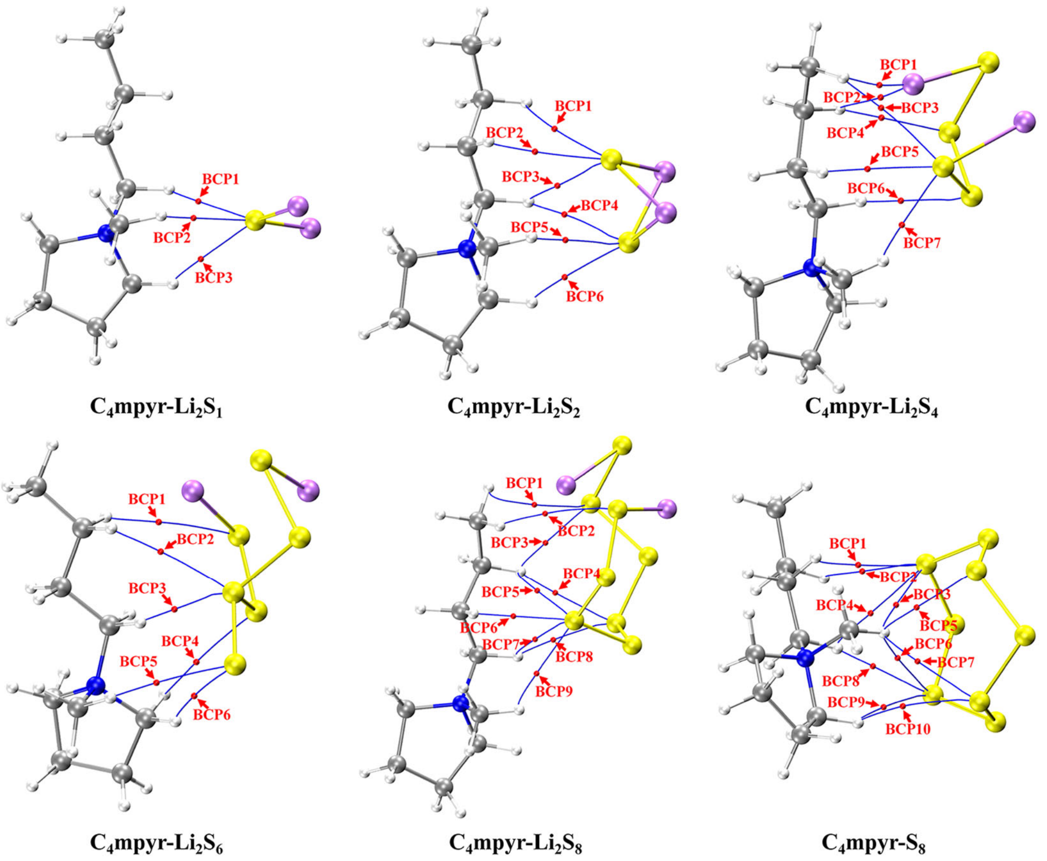

Figure S2. Cations with different lithium polysulfide AIM molecular graphs.

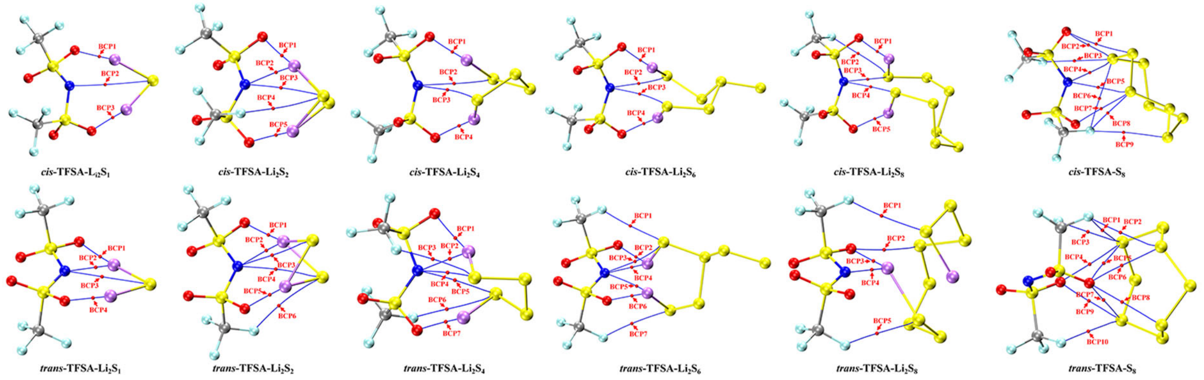

Figure S3. [TFSA]- anions with different lithium polysulfide AIM molecular graphs.

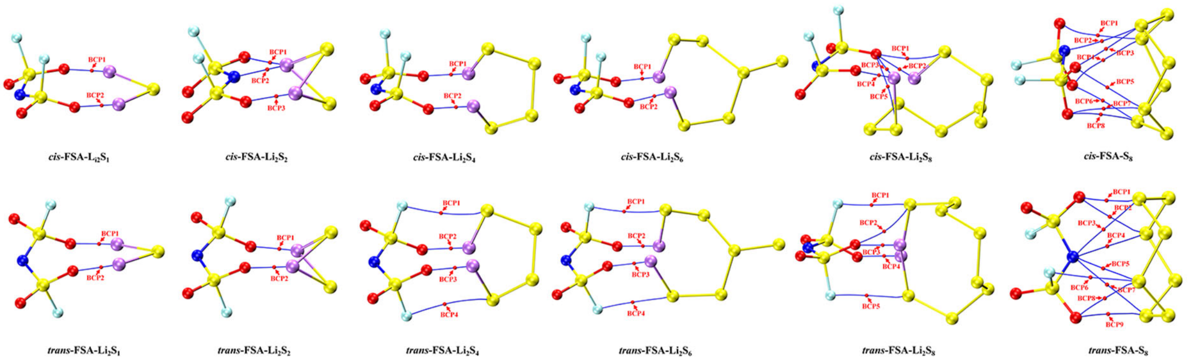

Figure S4. [FSA]- anions with different lithium polysulfide AIM molecular graphs.

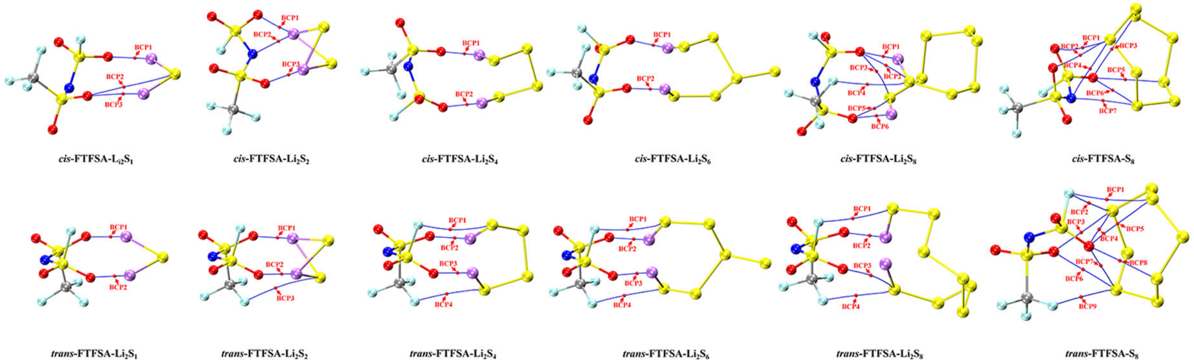

Figure S5. [FTFSA]$^-$ anions with different lithium polysulfide AIM molecular graphs.

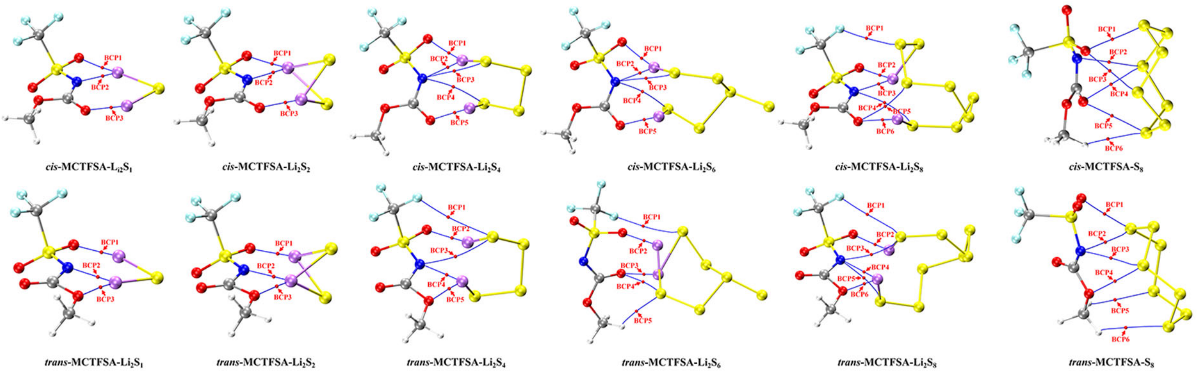

Figure S6. [MCTFSA]$^-$ anions with different lithium polysulfide AIM molecular graphs.

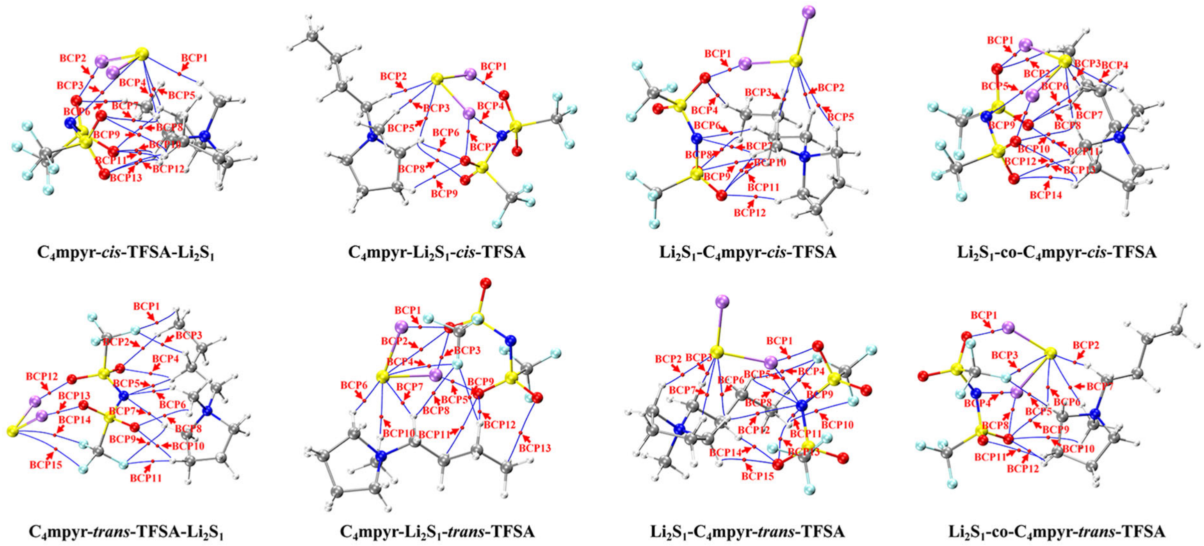

Figure S7. Different configurations of [TFSA]$^-$ anionic liquid and Li_2S_1 AIM molecular graphs.

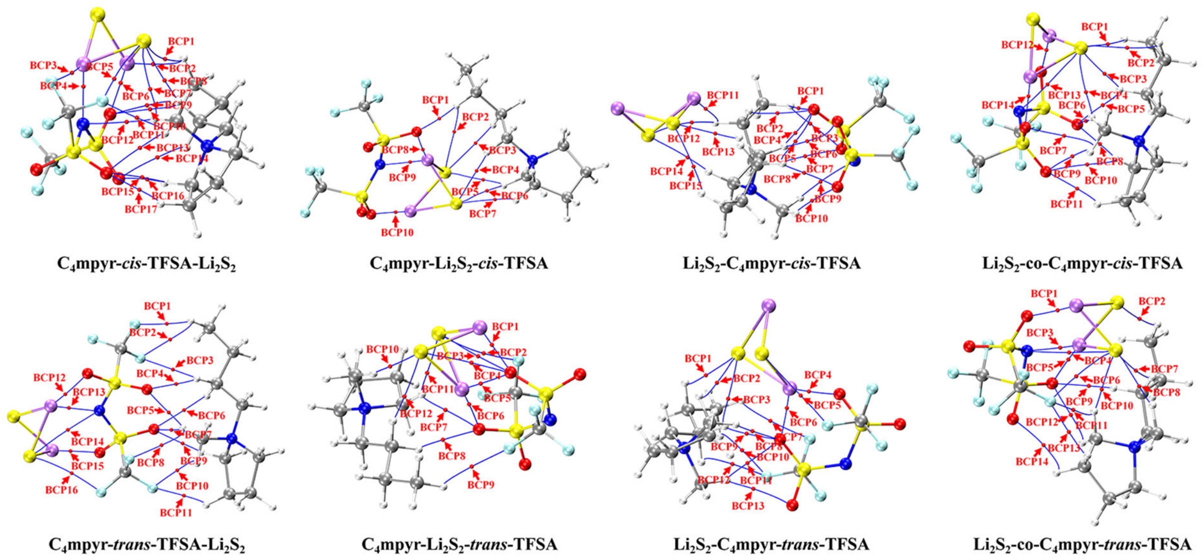

Figure S8. Different configurations of $[TFSA]^-$ anionic liquid and Li_2S_2 AIM molecular graphs.

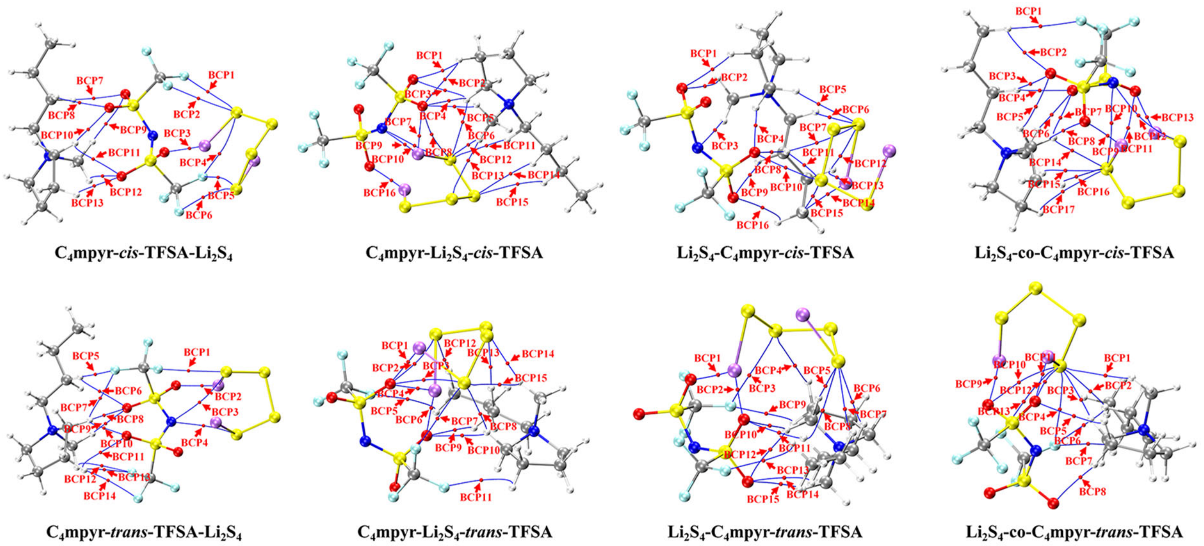

Figure S9. Different configurations of $[TFSA]^-$ anionic liquid and Li_2S_4 AIM molecular graphs.

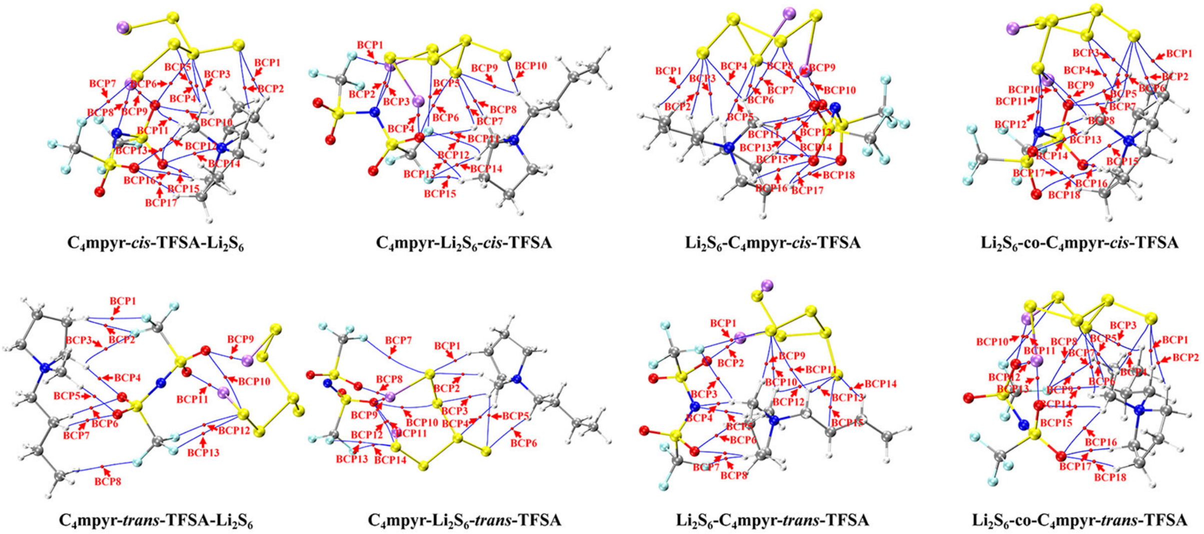

Figure S10. Different configurations of $[TFSA]^-$ anionic liquid and Li_2S_6 AIM molecular graphs.

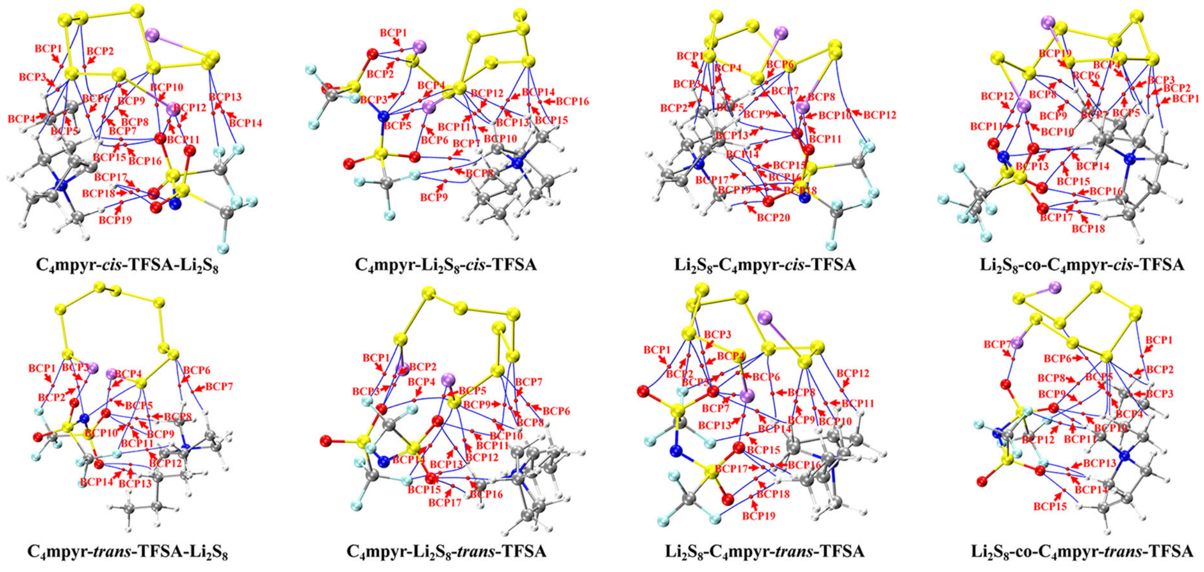

Figure S11. Different configurations of $[TFSA]^-$ anionic liquid and Li_2S_8 AIM molecular graphs.

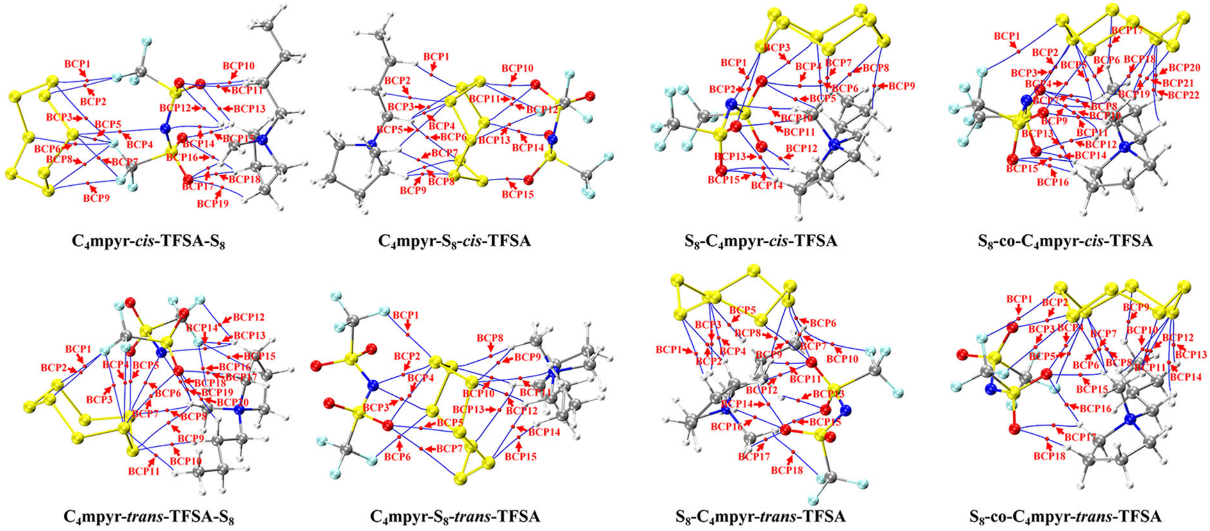

Figure S12. Different configurations of $[TFSA]^-$ anionic liquid and S_8 AIM molecular graphs.

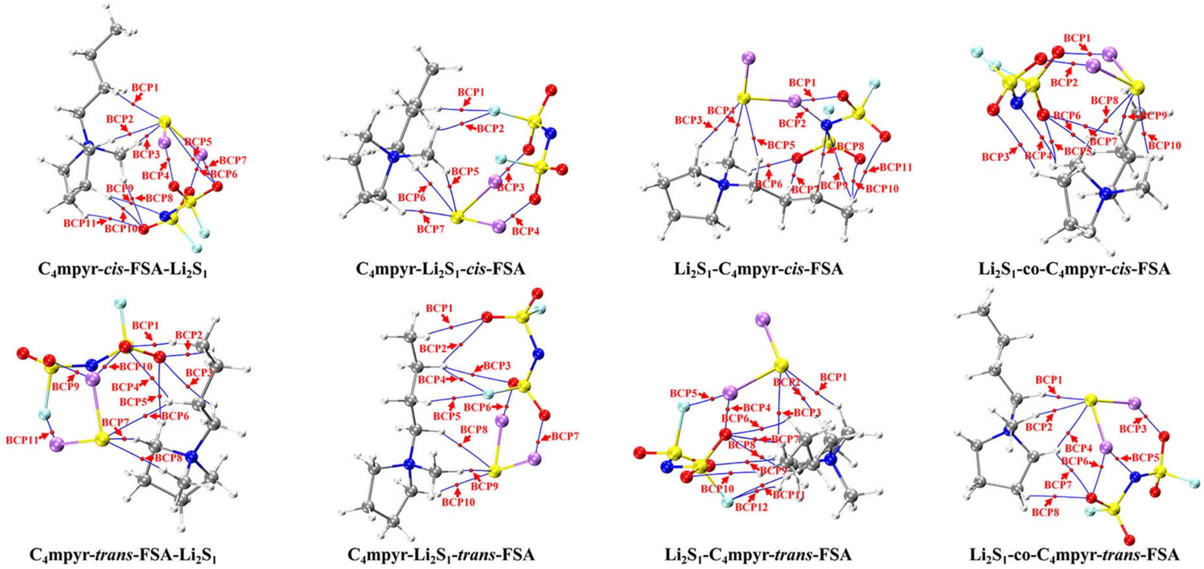

Figure S13. Different configurations of $[FSA]^-$ anionic liquid and Li_2S_1 AIM molecular graphs.

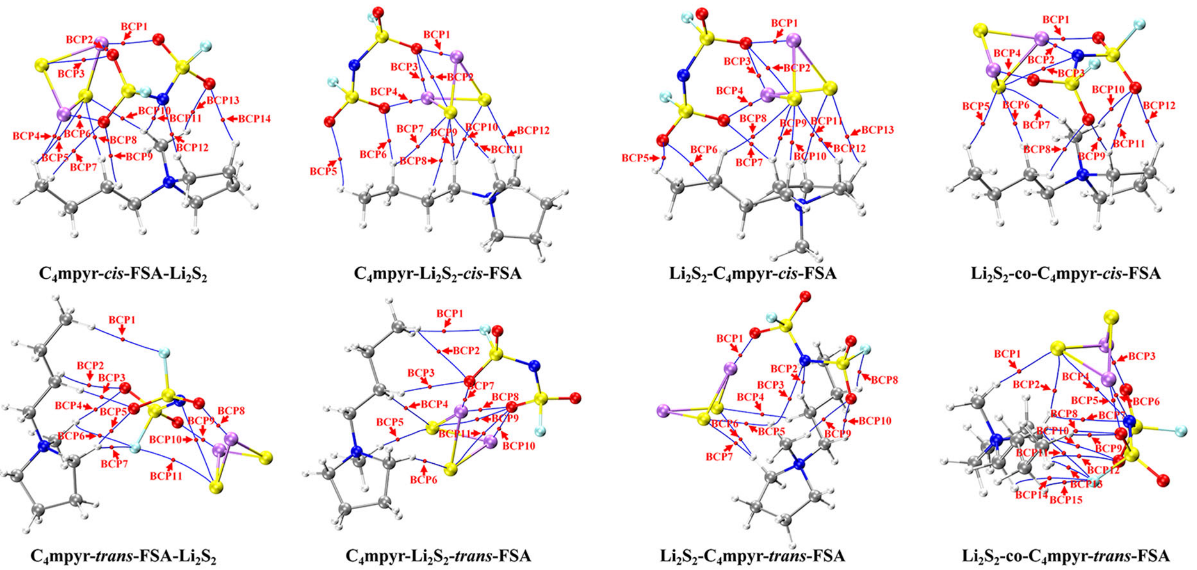

Figure S14. Different configurations of [FSA]$^-$ anionic liquid and Li_2S_2 AIM molecular graphs.

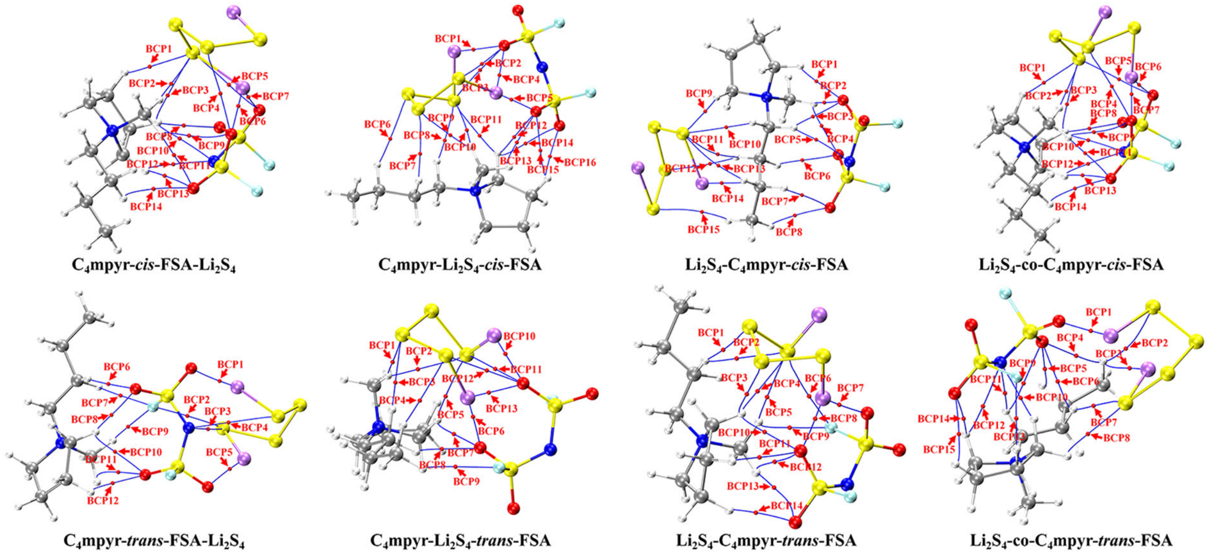

Figure S15. Different configurations of [FSA]$^-$ anionic liquid and Li_2S_4 AIM molecular graphs.

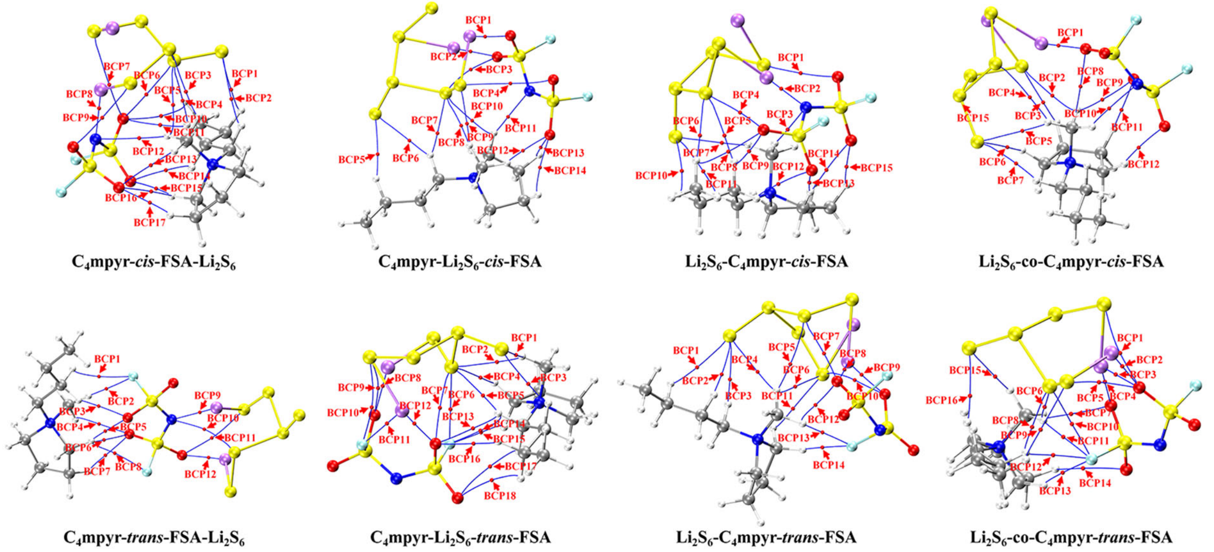

Figure S16. Different configurations of [FSA]$^-$ anionic liquid and Li_2S_6 AIM molecular graphs.

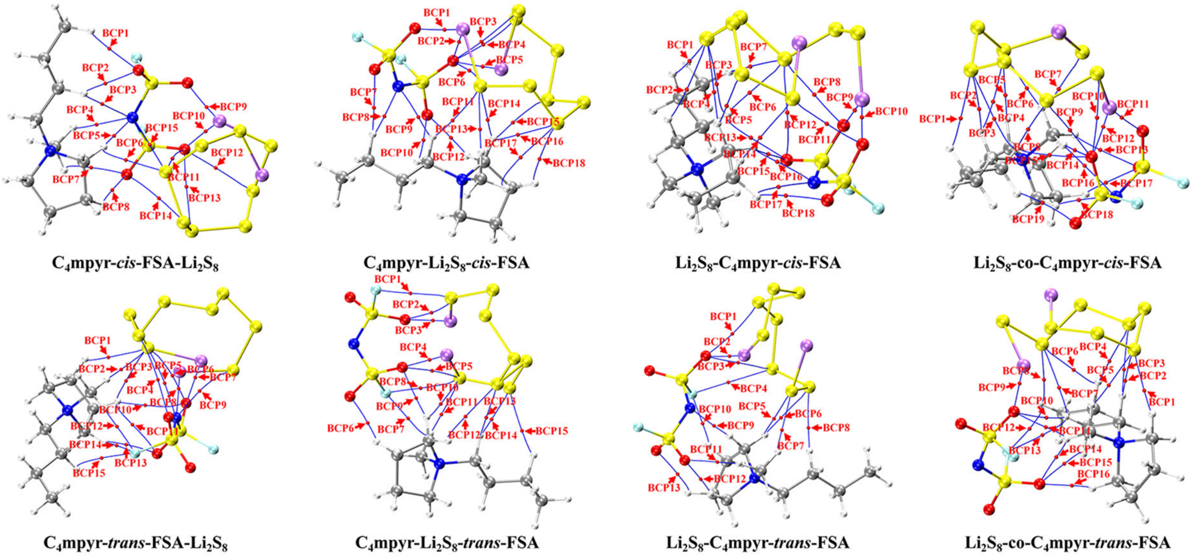

Figure S17. Different configurations of [FSA]$^-$ anionic liquid and Li_2S_8 AIM molecular graphs.

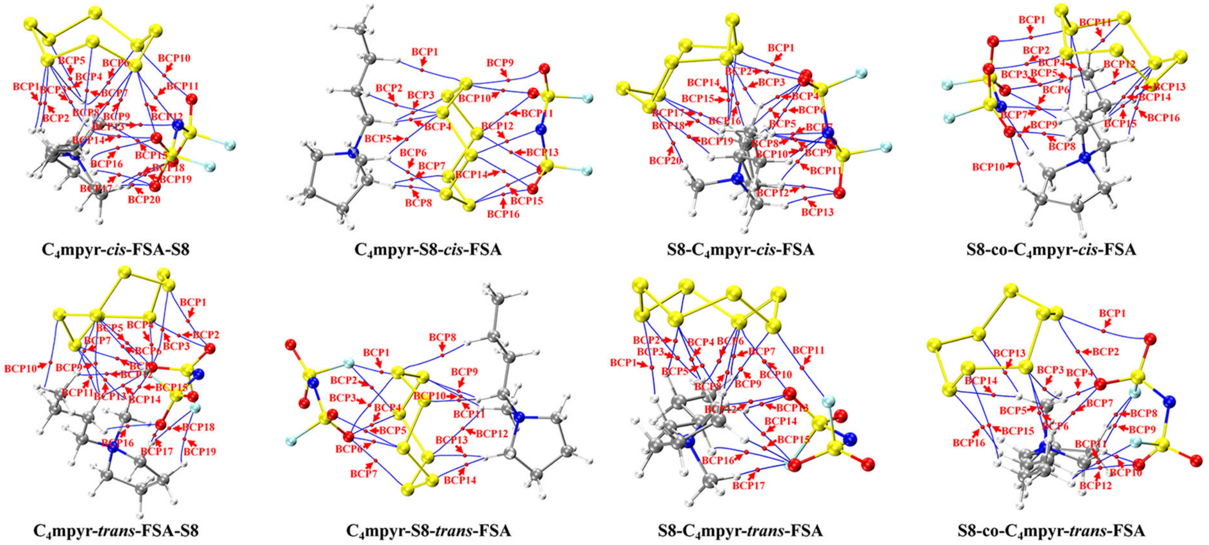

Figure S18. Different configurations of [FSA]$^-$ anionic liquid and S_8 AIM molecular graphs.

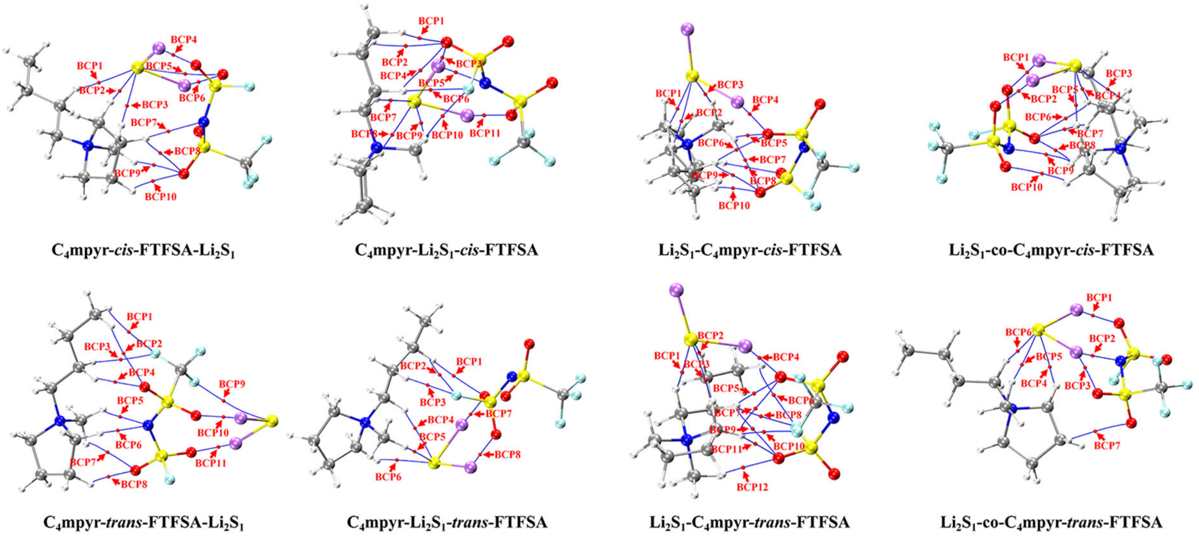

Figure S19. Different configurations of [FTFSA]$^-$ anionic liquid and Li_2S_1 AIM molecular graphs.

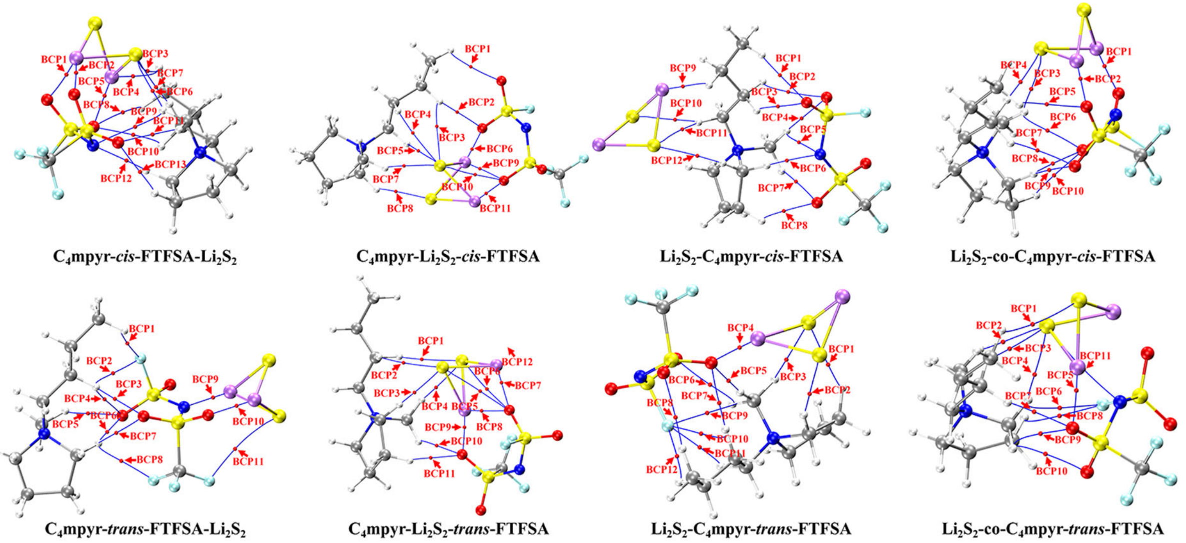

Figure S20. Different configurations of [FTFSA]⁻ anionic liquid and Li_2S_2 AIM molecular graphs.

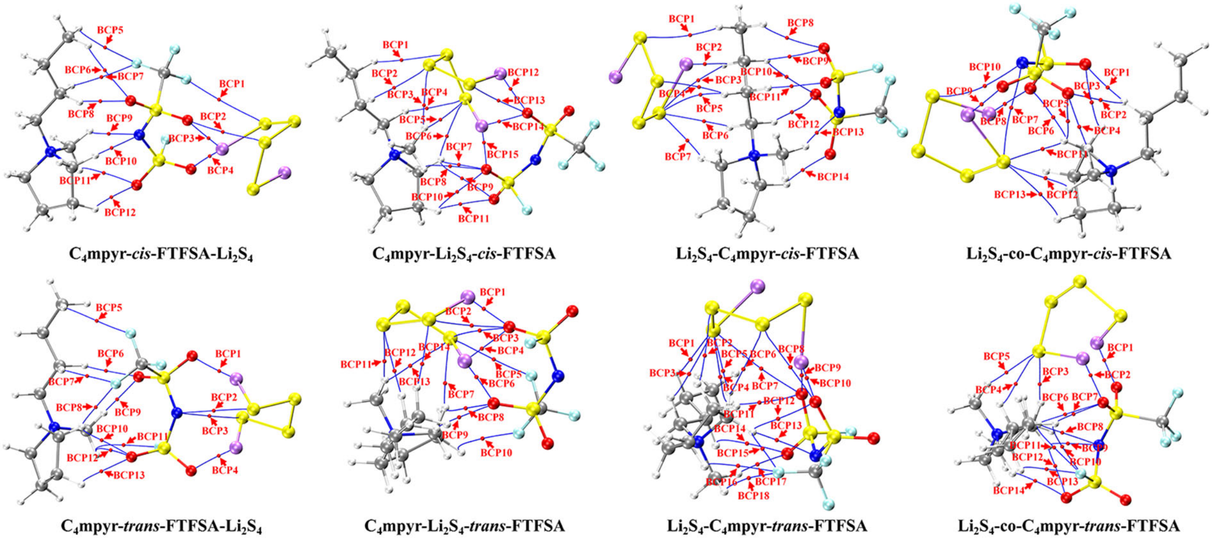

Figure S21. Different configurations of [FTFSA]⁻ anionic liquid and Li_2S_4 AIM molecular graphs.

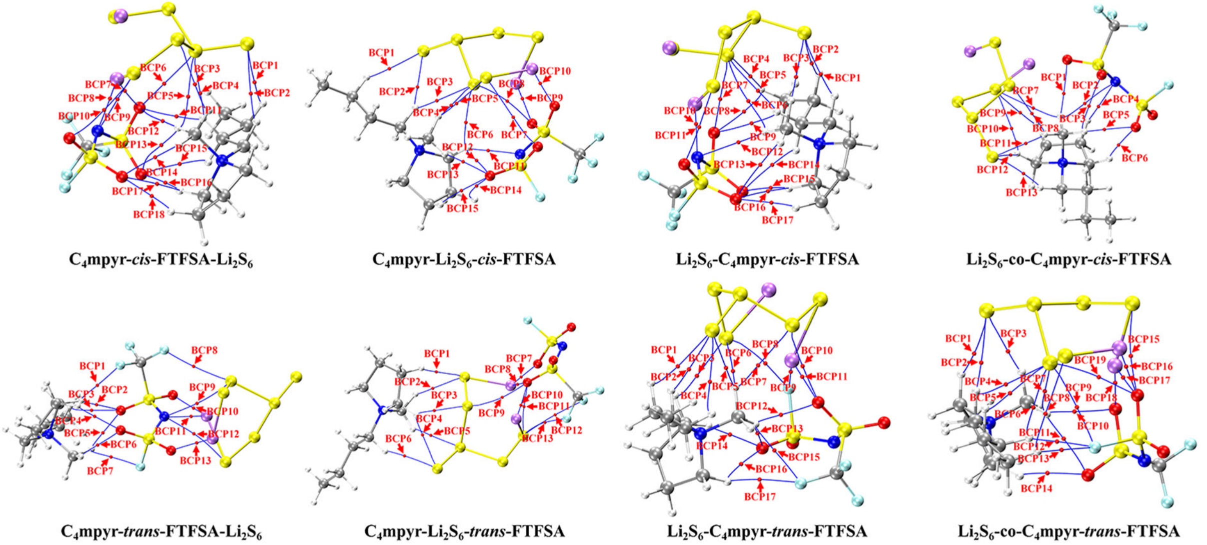

Figure S22. Different configurations of [FTFSA]⁻ anionic liquid and Li_2S_6 AIM molecular graphs.

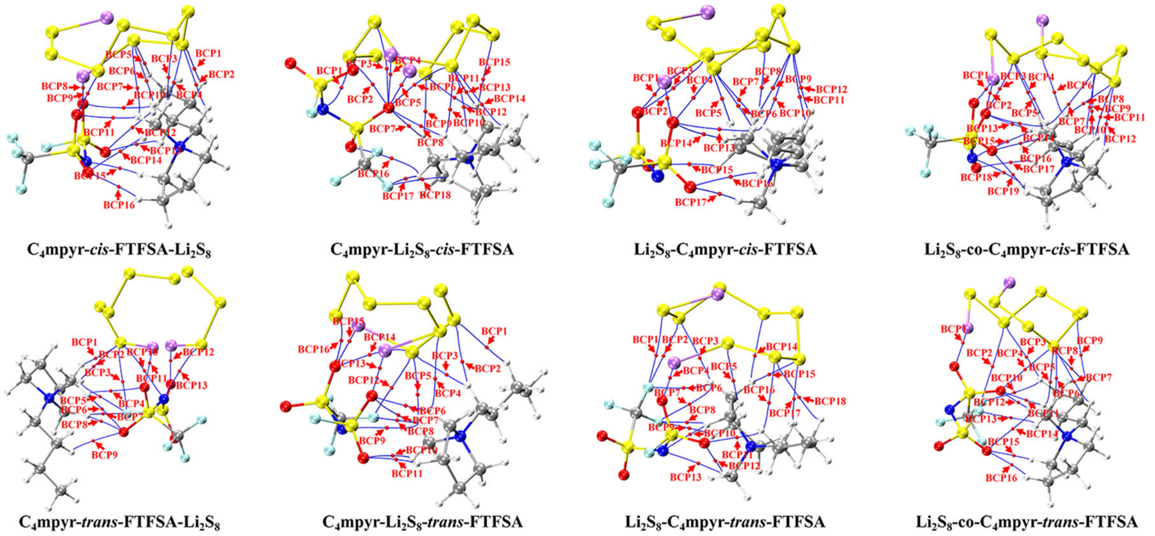

Figure S23. Different configurations of $[FTFSA]^-$ anionic liquid and Li_2S_8 AIM molecular graphs.

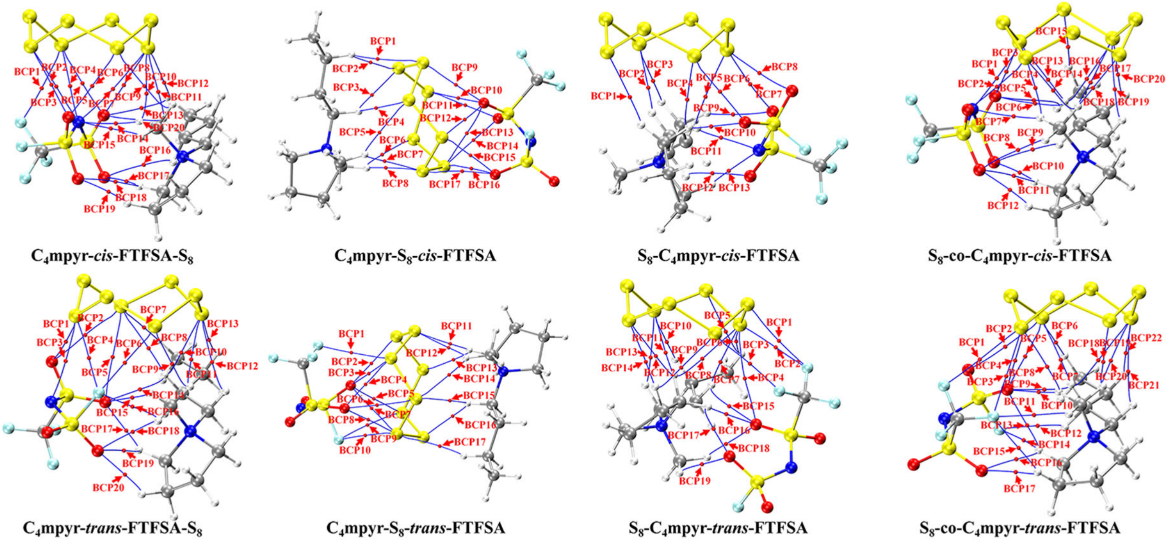

Figure S24. Different configurations of $[FTFSA]^-$ anionic liquid and S_8 AIM molecular graphs.

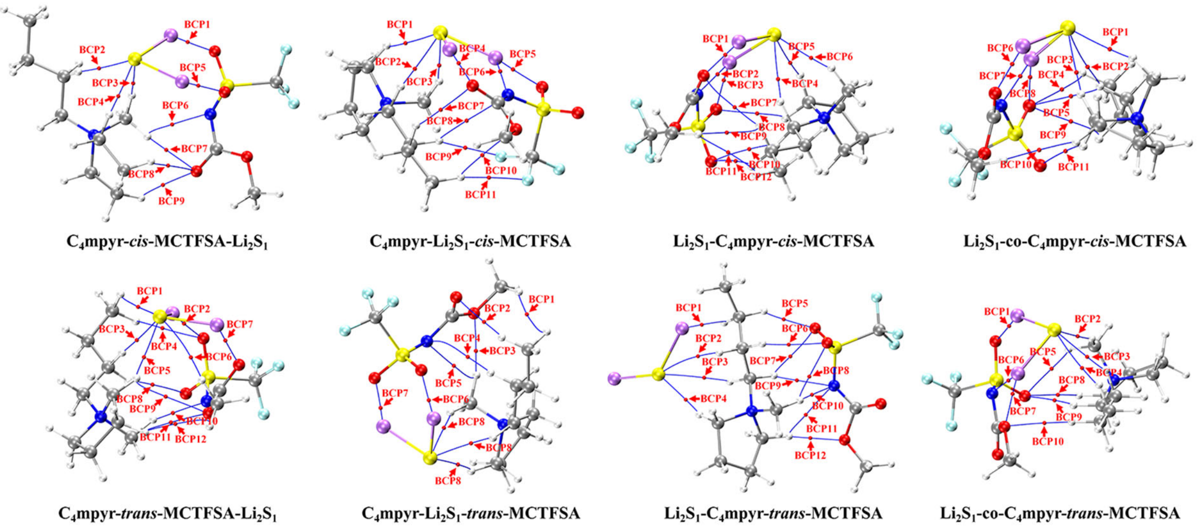

Figure S25. Different configurations of $[MCTFSA]^-$ anionic liquid and Li_2S_1 AIM molecular graphs.

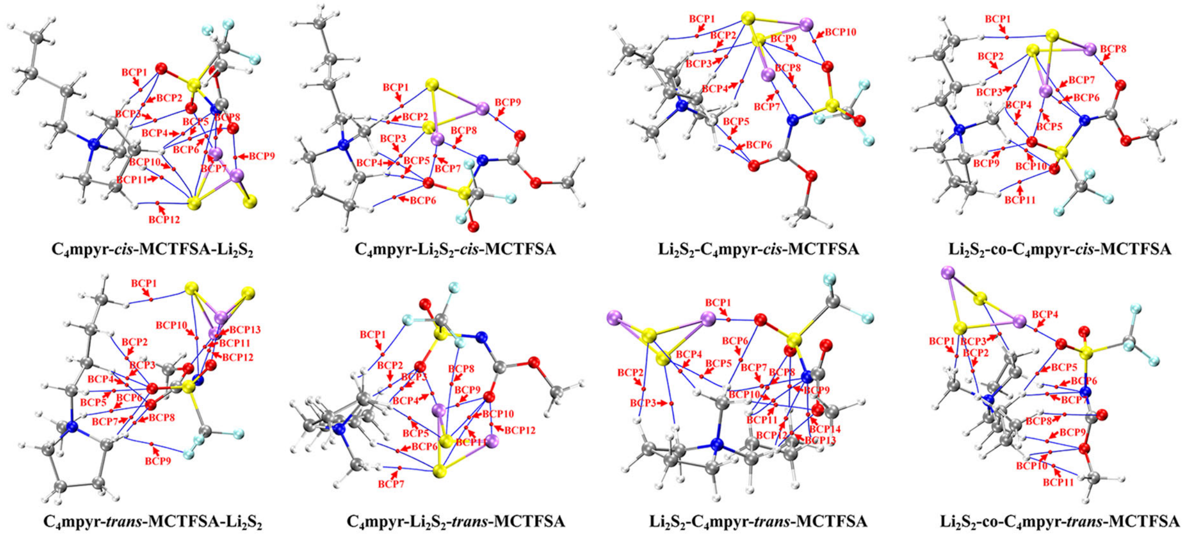

Figure S26. Different configurations of [MCTFSA]$^{-}$ anionic liquid and Li_2S_2 AIM molecular graphs.

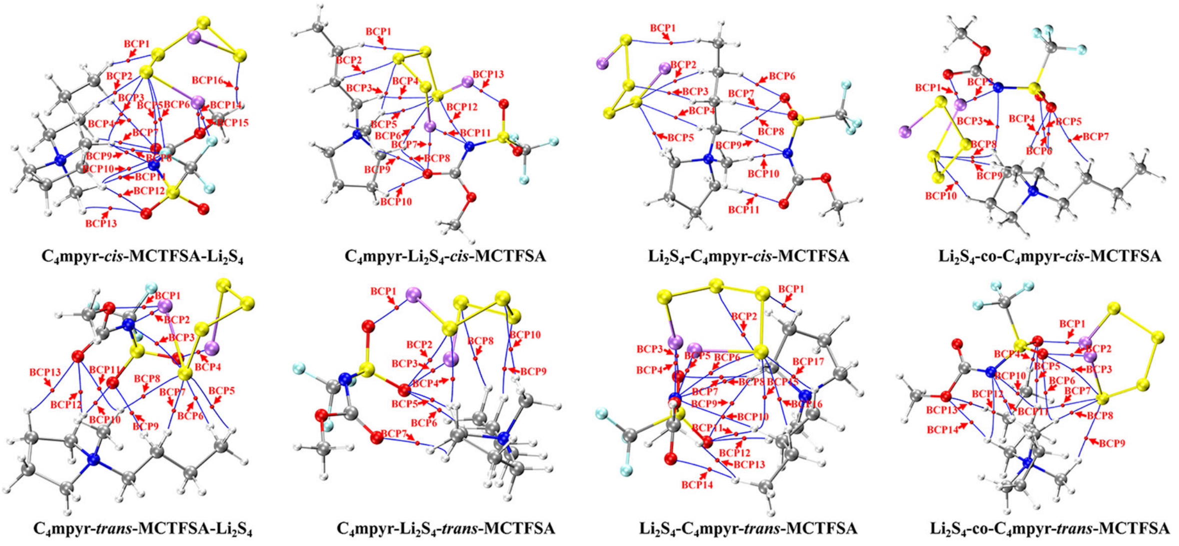

Figure S27. Different configurations of [MCTFSA]$^{-}$ anionic liquid and Li_2S_4 AIM molecular graphs.

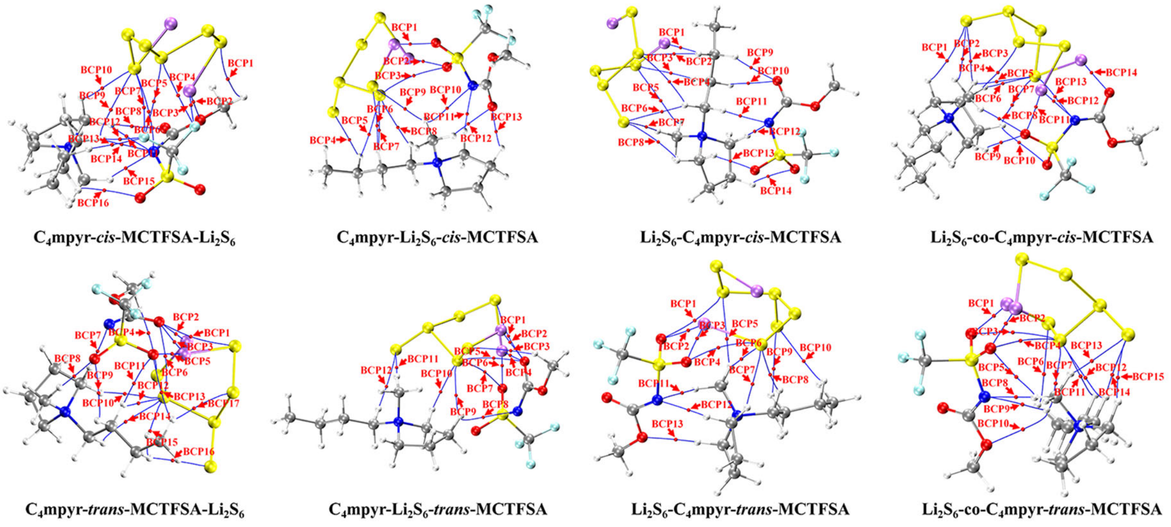

Figure S28. Different configurations of [MCTFSA]$^{-}$ anionic liquid and Li_2S_6 AIM molecular graphs.

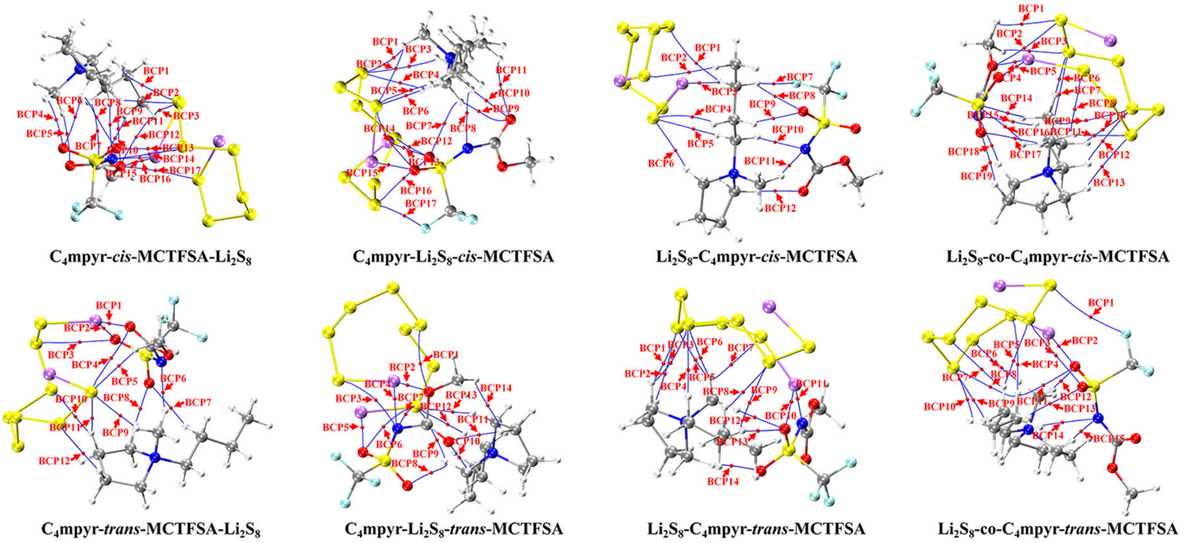

Figure S29. Different configurations of [MCTFSA]$^-$ anionic liquid and Li_2S_8 AIM molecular graphs.

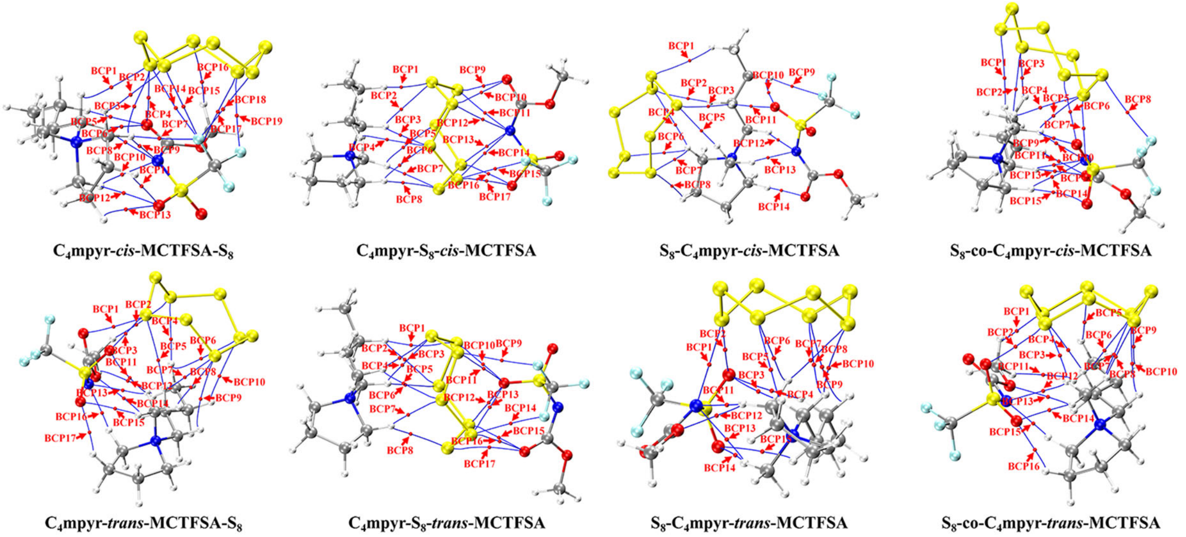

Figure S30. Different configurations of [MCTFSA]$^-$ anionic liquid and S_8 AIM molecular graphs.

Table S1. The AIM data calculated with M06-2x for all the studied complexes[a].

	Interaction	No.BCP	E_B (meV)		Interaction	No.BCP	E_B (meV)
Ion-pair							
C$_4$mpyr-*cis*-TFSA	C-H···O	1	-48.0	C$_4$mpyr-*trans*-TFSA	C-H···O	1	-114.3
	C-H···O	2	-98.1		C-H···O	2	-81.2
	C-H···O	3	-97.2		C-H···F	3	-70.0
	C-H···O	4	-159.2		C-H···O	4	-89.2
	C-H···N	5	-88.1		C-H···F	5	-41.4
	C-H···O	6	-71.2		C-H···N	6	-116.9
	C-H···O	7	-156.2		C-H···N	7	-137.9
	C-H···O	8	-106.7		C-H···N	8	-76.9
	C-H···O	9	-67.4		C-H···F	9	-71.7
					C-H···O	10	-106.0
					C-H···O	11	-89.7

					C-H···O	12	-106.9
C_4mpyr-*cis*-FSA	C-H···O	1	-52.9	C_4mpyr-*trans*-FSA	C-H···F	1	-104.8
	C-H···O	2	-58.9		C-H···O	2	-117.1
	C-H···O	3	-133.4		C-H···F	3	-69.1
	C-H···O	4	-104.9		C-H···O	4	-109.9
	C-H···N	5	-90.2		C-H···O	5	-118.5
	C-H···N	6	-113.0		C-H···O	6	-105.0
	C-H···O	7	-97.5		C-H···O	7	-119.9
	C-H···O	8	-79.0		C-H···F	8	-123.0
	C-H···O	9	-134.1		C-H···O	9	-168.5
C_4mpyr-*cis*-FTFSA	C-H···O	1	-51.6	C_4mpyr-*trans*-FTFSA	C-H···F	1	-56.3
	C-H···O	2	-73.2		C-H···O	2	-154.2
	C-H···O	3	-110.8		C-H···O	3	-166.2
	C-H···N	4	-118.2		C-H···O	4	-155.1
	C-H···O	5	-124.4		C-H···F	5	-108.7
	C-H···N	6	-75.8		C-H···O	6	-113.4
	C-H···O	7	-93.6		C-H···F	7	-135.5
	C-H···O	8	-106.2				
	C-H···O	9	-100.4				
	C-H···O	10	-75.5				
C_4mpyr-*cis*-MCTFSA	C-H···O	1	-136.3	C_4mpyr-*trans*-MCTFSA	C-H···O	1	-103.4
	C-H···N	2	-67.8		C-H···O	2	-69.5
	C-H···O	3	-135.0		C-H···F	3	-62.5
	C-H···N	4	-134.3		C-H···O	4	-139.7
	C-H···O	5	-118.9		C-H···F	5	-75.3
	C-H···O	6	-106.6		C-H···O	6	-117.7
	C-H···O	7	-85.1		C-H···N	7	-109.6
	C-H···O	8	-86.7		C-H···N	8	-132.4
					C-H···N	9	-119.1
					C-H···O	10	-128.8
Cation-LiPSs							
C_4mpyr-Li_2S_1	C-H···S	1	-100.4	C_4mpyr-Li_2S_2	C-H···S	1	-33.0
	C-H···S	2	-99.9		C-H···S	2	-46.1
	C-H···S	3	-85.9		C-H···S	3	-80.1
					C-H···S	4	-73.9
					C-H···S	5	-82.9
					C-H···S	6	-61.3
C_4mpyr-Li_2S_4	C-H···Li	1	-59.1	C_4mpyr-Li_2S_6	C-H···S	1	-34.7
	C-H···Li	2	-70.6		C-H···S	2	-26.6

	C-H···S	3	-39.6		C-H···S	3	-122.5
	C-H···S	4	-61.7		C-H···S	4	-40.8
	C-H···S	5	-58.8		C-H···S	5	-32.8
	C-H···S	6	-83.5		C-H···S	6	-45.2
	C-H···S	7	-77.8				
C_4mpyr-Li_2S_8	C-H···S	1	-33.2	C_4mpyr-S_8	C-H···S	1	-45.8
	C-H···S	2	-37.6		C-H···S	2	-42.4
	C-H···S	3	-56.3		C-H···S	3	-49.6
	C-H···S	4	-37.4		C-H···S	4	-39.2
	C-H···S	5	-47.5		C-H···S	5	-50.3
	C-H···S	6	-70.9		C-H···S	6	-33.7
	C-H···S	7	-55.4		C-H···S	7	-41.2
	C-H···S	8	-44.1		C-H···S	8	-69.2
	C-H···S	9	-53.2		C-H···S	9	-46.1
					C-H···S	10	-23.9
Anions-LiPSs							
cis-TFSA-Li_2S_1	Li···O	1	-389.3	*trans*-TFSA-Li_2S_1	O···Li	1	-209.4
	S···N	2	-41.9		N···Li	2	-185.6
	Li···O	3	-385.7		N···S	3	-77.8
					O···Li	4	-374.5
cis-TFSA-Li_2S_2	O···Li	1	-288.5	*trans*-TFSA-Li_2S_2	O···Li	1	-284.9
	N···Li	2	-157.7		N···Li	2	-155.4
	N···S	3	-66.3		N···S	3	-70.5
	F···S	4	-19.5		N···S	4	-71.0
	O···Li	5	-400.3		O···Li	5	-370.4
					F···S	6	-23.3
cis-TFSA-Li_2S_4	O···Li	1	-386.9	*trans*-TFSA-Li_2S_4	O···Li	1	-281.3
	N···S	2	-55.5		N···Li	2	-175.3
	N···S	3	-56.5		F···S	3	-40.4
	O···Li	4	-407.2		N···S	4	-79.3
					N···S	5	-75.4
					F···S	6	-19.6
					O···Li	7	-371.1
cis-TFSA-Li_2S_6	O···Li	1	-365.7	*trans*-TFSA-Li_2S_6	F···S	1	-35.6
	N···S	2	-71.7		O···Li	2	-307.2
	N···S	3	-73.9		N···S	3	-82.1
	O···Li	4	-396.2		N···Li	4	-236.0
					N···S	5	-75.6
					O···Li	6	-393.0

					F···S	7	-47.6
cis-TFSA-Li_2S_8	O···Li	1	-383.6	*trans*-TFSA-Li_2S_8	F···S	1	-36.3
	F···S	2	-29.9		O···S	2	-59.8
	N···S	3	-97.4		O···Li	3	-361.3
	N···S	4	-87.5		N···Li	4	-249.7
	O···Li	5	-378.0		F···S	5	-35.0
cis-TFSA-S_8	O···S	1	-62.4	*trans*-TFSA-S_8	F···S	1	-35.4
	O···S	2	-76.9		F···S	2	-41.0
	F···S	3	-33.1		F···S	3	-75.5
	N···S	4	-63.3		O···S	4	-71.7
	N···S	5	-79.6		O···S	5	-63.7
	F···S	6	-21.2		O···S	6	-44.1
	O···S	7	-71.6		O···S	7	-54.2
	F···S	8	-46.8		O···S	8	-32.7
	F···S	9	-17.2		O···S	9	-88.7
					F···S	10	-62.1
cis-FSA-Li_2S_1	O···Li	1	-464.3	*trans*-FSA-Li_2S_1	O···Li	1	-426.5
	O···Li	2	-458.2		O···Li	2	-426.5
cis-FSA-Li_2S_2	O···Li	1	-216.2	*trans*-FSA-Li_2S_2	O···Li	1	-439.8
	N···Li	2	-204.1		O···Li	2	-439.8
	O···Li	3	-387.9				
cis-FSA-Li_2S_4	O···Li	1	-472.1	*trans*-FSA-Li_2S_4	F···S	1	-31.5
	O···Li	2	-446.1		O···Li	2	-426.2
					O···Li	3	-438.2
					F···S	4	-37.4
cis-FSA-Li_2S_6	O···Li	1	-479.1	*trans*-FSA-Li_2S_6	F···S	1	-47.8
	O···Li	2	-495.7		O···Li	2	-477.0
					O···Li	3	-441.5
					F···S	4	-36.3
cis-FSA-Li_2S_8	O···Li	1	-141.4	*trans*-FSA-Li_2S_8	O···Li	1	-437.6
	O···Li	2	-333.0		O···S	2	-84.9
	O···Li	3	-257.7		F···S	3	-59.2
	O···Li	4	-444.0		F···S	4	-43.6
	O···Li	5	-117.8		O···Li	5	-453.5
cis-FSA-S_8	O···S	1	-59.5	*trans*-FSA-S_8	O···S	1	-89.9
	O···S	2	-42.7		O···S	2	-100.9

	O···S	3	-68.5		N···S	3	-45.1
	O···S	4	-62.6		N···S	4	-42.8
	O···S	5	-76.1		N···S	5	-38.7
	O···S	6	-75.5		F···S	6	-43.0
	O···S	7	-54.6		N···S	7	-35.9
	O···S	8	-50.0		O···S	8	-86.0
					O···S	9	-85.9
cis-FTFSA-Li_2S_1	O···Li	1	-248.2	*trans*-FTFSA-Li_2S_1	O···Li	1	-456.4
	O···S	2	-21.4		O···Li	2	-444.7
	O···Li	3	-38.5				
cis-FTFSA-Li_2S_2	O···Li	1	-242.9	*trans*-FTFSA-Li_2S_2	O···Li	1	-462.7
	N···Li	2	-187.0		O···Li	2	-455.6
	O···Li	3	-406.5		F···S	3	-28.7
cis-FTFSA-Li_2S_4	O···Li	1	-471.0	*trans*-FTFSA-Li_2S_4	F···S	1	-27.5
	O···Li	2	-450.0		O···Li	2	-463.7
					O···Li	3	-462.1
					F···S	4	-49.3
cis-FTFSA-Li_2S_6	O···Li	1	-518.2	*trans*-FTFSA-Li_2S_6	F···S	1	-32.3
	O···Li	2	-468.6		O···Li	2	-510.9
					O···Li	3	-463.7
					F···S	4	-43.6
cis-FTFSA-Li_2S_8	O···Li	1	-457.2	*trans*-FTFSA-Li_2S_8	F···S	1	-37.6
	O···S	2	-102.7		O···Li	2	-490.7
	O···S	3	-95.0		O···S	3	-98.3
	F···S	4	-17.2		F···S	4	-52.8
	O···S	5	-110.6				
	O···Li	6	-522.1				
cis-FTFSA-S_8	O···S	1	-60.5	*trans*-FTFSA-S_8	F···S	1	-28.7
	O···S	2	-85.2		F···S	2	-76.7
	O···S	3	-41.0		O···S	3	-90.1
	N···S	4	-56.0		O···S	4	-53.9
	O···S	5	-49.2		O···S	5	-57.1
	O···S	6	-48.0		O···S	6	-80.9
	N···S	7	-83.7		O···S	7	-63.9
					O···S	8	-66.2
					F···S	9	-44.5
cis-MCTFSA-Li_2S_1	O···Li	1	-196.6	*trans*-MCTFSA-Li_2S_1	O···Li	1	-425.2

	N···Li	2	-258.2		N···Li	2	-281.8
	O···Li	3	-429.5		O···Li	3	-204.2
cis-MCTFSA-Li_2S_2	O···Li	1	-236.9	*trans*-MCTFSA-Li_2S_2	O···Li	1	-428.2
	N···Li	2	-248.3		N···Li	2	-286.7
	O···Li	3	-427.3		O···Li	3	-227.8
cis-MCTFSA-Li_2S_4	O···Li	1	-272.8	*trans*-MCTFSA-Li_2S_4	F···S	1	-16.6
	N···Li	2	-262.7		O···Li	2	-415.1
	N···S	3	-89.5		N···S	3	-85.3
	N···S	4	-82.0		N···Li	4	-310.6
	O···Li	5	-414.9		O···Li	5	-234.6
cis-MCTFSA-Li_2S_6	O···Li	1	-323.6	*trans*-MCTFSA-Li_2S_6	F···S	1	-27.7
	N···Li	2	-271.1		O···Li	2	-570.1
	N···S	3	-85.1		O···Li	3	-568.0
	N···S	4	-101.6		O···S	4	-109.8
	O···Li	5	-441.6		C-H···S	5	-24.9
cis-MCTFSA-Li_2S_8	F···S	1	-24.5	*trans*-MCTFSA-Li_2S_8	F···S	1	-26.1
	O···Li	2	-288.0		O···Li	2	-427.3
	N···Li	3	-261.4		N···S	3	-107.1
	N···S	4	-89.1		N···Li	4	-296.8
	O···S	5	-95.8		N···S	5	-105.3
	O···Li	6	-513.9		O···Li	6	-271.7
cis-MCTFSA-S_8	O···S	1	-85.0	*trans*-MCTFSA-S_8	O···S	1	-120.7
	N···S	2	-50.9		N···S	2	-60.8
	O···S	3	-104.4		N···S	3	-124.4
	O···S	4	-83.9		O···S	4	-85.1
	O···S	5	-59.6		C-H···S	5	-32.2
	C-H···S	6	-51.8		C-H···S	6	-23.5
ILs-LiPSs							
C_4mpyr-*cis*-TFSA-Li_2S_1	C-H···S	1	-119.6	C_4mpyr-*trans*-TFSA-Li_2S_1	C-H···F	1	-28.6
	O···Li	2	-460.1		C-H···O	2	-87.7
	N···S	3	-45.1		C-H···F	3	-72.9
	C-H···S	4	-132.0		C-H···O	4	-99.6
	C-H···S	5	-72.2		C-H···N	5	-60.1
	C-H···O	6	-62.0		C-H···N	6	-88.8
	C-H···O	7	-36.9		C-H···N	7	-130.7
	C-H···O	8	-94.1		C-H···O	8	-86.9
	C-H···O	9	-38.9		C-H···F	9	-72.9
	C-H···O	10	-69.8		C-H···O	10	-117.1

	C-H···O	11	-93.7		C-H···F	11	-60.3
	C-H···O	12	-55.3		O···Li	12	-376.5
	C-H···O	13	-104.8		O···Li	13	-375.5
					F···S	14	-61.6
					F···S	15	-43.6
C_4mpyr-Li_2S_1-*cis*-TFSA	O···Li	1	-465.4	C_4mpyr-Li_2S_1-*trans*-TFSA	C-H···S	1	-153.7
	C-H···S	2	-135.9		O···Li	1	-336.5
	C-H···S	3	-139.5		O···S	2	-138.7
	N···Li	4	-196.8		O···Li	3	-297.3
	C-H···S	5	-81.0		F···S	4	-28.2
	C-H···O	6	-69.8		O···Li	5	-445.0
	O···Li	7	-320.8		C-H···S	6	-130.3
	C-H···O	8	-75.1		C-H···S	7	-110.1
	C-H···O	9	-62.5		C-H···F	8	-28.2
					C-H···F	9	-56.0
					C-H···O	11	-37.4
					C-H···O	12	-79.9
					C-H···O	13	-54.8
Li_2S_1-C_4mpyr-*cis*-TFSA	O···Li	1	-555.4	Li_2S_1-C_4mpyr-*trans*-TFSA	O···Li	1	-431.2
	C-H···S	2	-131.8		C-H···S	2	-115.3
	C-H···S	3	-127.4		C-H···S	3	-112.2
	C-H···O	4	-51.6		C-H···F	4	-52.2
	C-H···S	5	-34.4		N···Li	5	-216.1
	C-H···N	6	-102.1		C-H···S	6	-100.8
	C-H···N	7	-87.0		C-H···S	7	-59.7
	C-H···O	8	-50.0		C-H···N	8	-22.4
	C-H···O	9	-48.6		C-H···F	9	-64.2
	C-H···O	10	-133.8		C-H···F	10	-37.8
	C-H···O	11	-51.5		C-H···N	11	-21.2
	C-H···O	12	-124.3		C-H···N	12	-29.1
					C-H···O	13	-24.9
					C-H···O	14	-150.2
					C-H···O	15	-103.2
Li_2S_1-co-C_4mpyr-*cis*-TFSA	O···Li	1	-459.9	Li_2S_1-co-C_4mpyr-*trans*-TFSA	O···Li	1	-428.1
	C-H···O	2	-62.1		C-H···S	2	-150.7
	C-H···S	3	-72.1		F···S	3	-18.6
	C-H···S	4	-119.7		N···Li	4	-265.7
	N···S	5	-45.1		C-H···F	5	-84.4
	C-H···O	6	-94.1		C-H···S	6	-64.1
	C-H···S	7	-131.9		C-H···S	7	-133.7
	C-H···O	8	-69.7		O···Li	8	-280.8

	O···Li	9	-454.6		C-H···O	9	-59.1
	C-H···O	10	-36.9		C-H···O	10	-27.5
	C-H···O	11	-93.6		C-H···O	11	-61.0
	C-H···O	12	-39.0		C-H···O	12	-71.2
	C-H···O	13	-104.7				
	C-H···O	14	-55.4				
C_4mpyr-*cis*-TFSA-Li_2S_2	C-H···S	1	-42.2	C_4mpyr-*trans*-TFSA-Li_2S_2	C-H···F	1	-56.1
	C-H···Li	2	-60.4		C-H···F	2	-67.1
	F···Li	3	-183.3		C-H···F	3	-32.5
	N···Li	4	-234.8		C-H···O	4	-90.6
	F···S	5	-24.2		C-H···O	5	-146.8
	O···Li	6	-428.5		C-H···O	6	-69.5
	C-H···S	7	-69.9		C-H···O	7	-136.0
	C-H···S	8	-108.1		C-H···F	8	-57.8
	C-H···O	9	-65.9		C-H···O	9	-156.1
	C-H···O	10	-59.3		C-H···F	10	-73.5
	C-H···F	11	-78.0		C-H···F	11	-108.6
	C-H···S	12	-67.4		O···Li	12	-195.6
	C-H···O	13	-81.3		N···Li	13	-171.3
	C-H···O	14	-65.4		N···S	14	-81.2
	C-H···O	15	-79.6		O···Li	15	-334.3
	C-H···O	16	-133.2		F···S	16	-69.0
	C-H···O	17	-116.2				
C_4mpyr-Li_2S_2-*cis*-TFSA	C-H···O	1	-27.4	C_4mpyr-Li_2S_2-*trans*-TFSA	O···Li	1	-338.6
	C-H···S	2	-34.1		C-H···S	2	-57.2
	C-H···S	3	-61.2		O···S	3	-125.4
	C-H···S	4	-113.3		O···S	4	-126.0
	C-H···S	5	-107.2		O···Li	5	-353.3
	C-H···S	6	-104.1		O···Li	6	-504.9
	C-H···S	7	-85.1		C-H···O	7	-59.7
	O···Li	8	-378.4		C-H···O	8	-118.7
	N···Li	9	-281.3		C-H···F	9	-24.8
	O···Li	10	-468.6		C-H···S	10	-118.3
					C-H···S	11	-114.3
					C-H···S	12	-73.3
Li_2S_2-C_4mpyr-*cis*-TFSA	C-H···O	1	-84.0	Li_2S_2-C_4mpyr-*trans*-TFSA	C-H···S	1	-51.3
	C-H···O	2	-77.4		C-H···S	2	-77.6
	C-H···O	3	-59.8		C-H···S	3	-70.6
	C-H···O	4	-158.5		O···Li	4	-435.3
	C-H···O	5	-70.2		F···S	5	-29.0
	C-H···O	6	-63.8		O···Li	6	-392.9

	C-H···O	7	-107.8		C-H···F	7	-19.0
	C-H···O	8	-114.3		C-H···O	8	-162.3
	C-H···N	9	-76.5		C-H···O	9	-108.4
	C-H···O	10	-143.8		C-H···F	10	-41.5
	C-H···Li	11	-134.0		C-H···O	11	-84.6
	C-H···S	12	-73.2		C-H···F	12	-62.9
	C-H···S	13	-104.4		C-H···O	13	-61.6
	C-H···S	14	-80.9				
	C-H···S	15	-56.1				
Li_2S_2-co-C_4mpyr-*cis*-TFSA	C-H···S	1	-27.8	Li_2S_2-co-C_4mpyr-*trans*-TFSA	O···Li	1	-417.8
	C-H···S	2	-36.5		C-H···S	2	-36.8
	C-H···S	3	-100.9		N···Li	3	-273.7
	C-H···S	4	-96.3		N···S	4	-85.3
	C-H···O	5	-99.3		O···Li	5	-261.1
	C-H···O	6	-66.7		F···S	6	-70.5
	C-H···O	7	-69.4		C-H···S	7	-77.8
	C-H···O	8	-117.5		C-H···S	8	-126.7
	C-H···O	9	-85.8		C-H···O	9	-115.6
	C-H···O	10	-40.2		C-H···S	10	-101.1
	C-H···O	11	-85.8		C-H···F	11	-64.0
	O···Li	12	-419.3		C-H···O	12	-81.9
	N···S	13	-61.9		C-H···F	13	-85.3
	O···Li	14	-329.4		C-H···O	14	-40.3
C_4mpyr-*cis*-TFSA-Li_2S_4	F···S	1	-49.0	C_4mpyr-*trans*-TFSA-Li_2S_4	C-H···S	1	-63.7
	F···S	2	-55.0		O···Li	2	-347.3
	O···Li	3	-446.9		N···S	3	-83.1
	F···S	4	-46.6		N···Li	4	-92.0
	F···S	5	-60.2		C-H···F	5	-81.7
	F···S	6	-48.8		C-H···O	6	-79.5
	C-H···O	7	-120.3		C-H···F	7	-50.8
	C-H···O	8	-84.1		C-H···O	8	-127.2
	C-H···O	9	-75.8		C-H···O	9	-162.6
	C-H···O	10	-145.8		C-H···O	10	-114.5
	C-H···O	11	-84.6		C-H···O	11	-71.3
	C-H···O	12	-119.4		C-H···F	12	-48.9
	C-H···O	13	-136.6		C-H···F	13	-43.4
					C-H···F	14	-29.3
C_4mpyr-Li_2S_4-*cis*-TFSA	C-H···O	1	-45.9	C_4mpyr-Li_2S_4-*trans*-TFSA	O···Li	1	-345.7
	C-H···O	2	-82.7		O···S	2	-131.0
	C-H···O	3	-97.6		O···S	3	-122.8
	C-H···O	4	-65.5		O···Li	4	-293.3

	C-H···O	5	-100.3		C-H···F	5	-9.6
	C-H···S	6	-78.8		O···Li	6	-488.8
	O···Li	7	-261.1		F···S	7	-40.3
	O···S	8	-62.7		C-H···S	8	-111.3
	N···Li	9	-245.7		C-H···O	9	-123.6
	N··S	10	-78.5		C-H···O	10	-58.9
	C-H···S	11	-104.4		C-H···S	11	-36.0
	C-H···S	12	-82.8		C-H···S	12	-48.9
	C-H···S	13	-67.2		C-H···S	13	-81.8
	C-H···S	14	-39.6		C-H···S	14	-104.7
	C-H···S	15	-29.6		C-H···S	15	-68.0
	O···Li	16	-423.8				
Li_2S_4-C_4mpyr-*cis*-TFSA	C-H···O	1	-104.5	Li_2S_4-C_4mpyr-*trans*-TFSA	O···Li	1	-425.8
	C-H···O	2	-129.4		O···Li	2	-431.5
	C-H···N	3	-86.4		F···S	3	-17.0
	C-H···O	4	-151.6		C-H···S	4	-36.9
	C-H···S	5	-61.8		C-H···S	5	-116.8
	C-H···S	6	-75.2		C-H···S	6	-94.6
	C-H···S	7	-44.9		C-H···S	7	-32.7
	C-H···O	8	-105.3		C-H···S	8	-41.3
	C-H···O	9	-92.1		C-H···S	9	-67.6
	O···S	10	-110.0		C-H···O	10	-111.0
	C-H···S	11	-35.7		C-H···O	11	-102.0
	C-H···S	12	-54.4		C-H···O	12	-70.1
	C-H···Li	13	-75.0		C-H···F	13	-30.1
	C-H···Li	14	-90.4		C-H···O	14	-65.9
	C-H···S	15	-66.5		C-H···O	15	-106.6
	C-H···O	16	-51.2				
Li_2S_4-co-C_4mpyr-*cis*-TFSA	C-H···F	1	-14.4	Li_2S_4-co-C_4mpyr-*trans*-TFSA	C-H···S	1	-62.5
	C-H···O	2	-21.4		C-H···S	2	-81.4
	C-H···O	3	-98.5		C-H···S	3	-113.0
	C-H···O	4	-98.1		F···S	4	-38.9
	C-H···O	5	-94.4		C-H···O	5	-43.4
	C-H···O	6	-138.4		C-H···F	6	-84.7
	C-H···O	7	-87.3		C-H···F	7	-36.8
	C-H···O	8	-93.6		C-H···O	8	-96.3
	O···Li	9	-375.6		O···Li	9	-393.1
	N··S	10	-51.4		O···S	10	-91.2
	O···S	11	-94.8		O···Li	11	-440.2
	N··S	12	-52.1		O···S	12	-90.6
	O···Li	13	-388.7		F···S	13	-20.6
	C-H···S	14	-103.5				

	C-H···S	15	-70.1				
	C-H···S	16	-61.8				
	C-H···S	17	-35.9				
C_4mpyr-*cis*-TFSA-Li_2S_6	C-H···S	1	-44.8	C_4mpyr-*trans*-TFSA-Li_2S_6	C-H···F	1	-34.3
	C-H···S	2	-52.6		C-H···F	2	-49.5
	C-H···S	3	-65.2		C-H···F	3	-46.1
	C-H···S	4	-58.5		C-H···O	4	-173.1
	C-H···S	5	-66.3		C-H···O	5	-185.9
	O···S	6	-59.7		C-H···O	6	-98.6
	F···S	7	-69.7		C-H···O	7	-87.1
	N···Li	8	-208.9		C-H···F	8	-17.0
	O···Li	9	-357.8		O···Li	9	-361.4
	C-H···O	10	-85.5		O···S	10	-97.6
	C-H···O	11	-44.1		O···Li	11	-430.3
	C-H···O	12	-40.0		F···S	12	-45.1
	C-H···O	13	-98.4		F···S	13	-30.0
	C-H···O	14	-95.9				
	C-H···O	15	-125.1				
	C-H···O	16	-51.7				
	C-H···O	17	-113.8				
C_4mpyr-Li_2S_6-*cis*-TFSA	F···Li	1	-211.7	C_4mpyr-Li_2S_6-*trans*-TFSA	C-H···S	1	-75.6
	N···Li	2	-221.6		C-H···S	2	-101.7
	N··S	3	-65.7		C-H···S	3	-89.7
	O···Li	4	-494.5		C-H···S	4	-91.4
	F···S	5	-32.3		C-H···S	5	-60.4
	O···S	6	-88.6		C-H···S	6	-69.1
	C-H···S	7	-76.8		F···S	7	-14.3
	C-H···S	8	-83.9		O···Li	8	-494.3
	C-H···S	9	-64.3		O···Li	9	-295.8
	C-H···S	10	-76.2		O···S	10	-97.0
	C-H···O	11	-94.2		O···Li	11	-443.9
	C-H···F	12	-71.6		O···S	12	-115.7
	C-H···O	13	-67.6		F···S	13	-32.2
	C-H···F	14	-105.2		F···S	14	-40.9
	C-H···F	15	-30.5				
Li_2S_6-C_4mpyr-*cis*-TFSA	C-H···S	1	-41.7	Li_2S_6-C_4mpyr-*trans*-TFSA	F···S	1	-31.1
	C-H···S	2	-58.9		O···Li	2	-602.7
	C-H···S	3	-59.2		C-H···F	3	-41.7
	C-H···S	4	-74.0		C-H···N	4	-162.3
	C-H···S	5	-48.2		C-H···N	5	-120.9
	C-H···S	6	-70.2		C-H···O	6	-51.9

	C-H···S	7	-52.8		C-H···O	7	-125.6
	O···S	8	-85.2		C-H···F	8	-31.7
	O···S	9	-48.3		C-H···S	9	-74.8
	O···Li	10	-365.6		C-H···S	10	-61.5
	C-H···O	11	-63.4		C-H···S	11	-34.6
	C-H···N	12	-90.8		C-H···S	12	-45.6
	C-H···O	13	-81.1		C-H···S	13	-33.0
	C-H···O	14	-55.8		C-H···S	14	-43.3
	C-H···O	15	-38.7		C-H···S	15	-52.6
	C-H···O	16	-69.9				
	C-H···O	17	-96.8				
	C-H···O	18	-97.8				
Li_2S_6-co-C_4mpyr-*cis*-TFSA	C-H···S	1	-30.8	Li_2S_6-co-C_4mpyr-*trans*-TFSA	C-H···S	1	-79.7
	C-H···S	2	-54.6		C-H···S	2	-49.4
	C-H···S	3	-42.5		C-H···S	3	-38.8
	C-H···S	4	-50.9		C-H···S	4	-99.8
	C-H···S	5	-71.3		C-H···S	5	-47.8
	C-H···S	6	-33.6		C-H···S	6	-87.4
	C-H···O	7	-42.6		C-H···S	7	-48.6
	C-H···O	8	-55.8		F···S	8	-30.1
	C-H···S	9	-72.1		C-H···F	9	-49.1
	O···Li	10	-326.3		O···Li	10	-340.1
	N··S	11	-67.2		F···S	11	-24.8
	C-H···O	12	-44.2		O···Li	12	-329.0
	C-H···N	13	-75.1		O···Li	13	-458.6
	C-H···O	14	-97.8		C-H···F	14	-82.1
	C-H···O	15	-106.5		C-H···O	15	-68.1
	C-H···O	16	-109.4		C-H···O	16	-72.3
	C-H···O	17	-95.2		C-H···O	17	-75.0
	C-H···O	18	-46.3		C-H···O	18	-84.9
C_4mpyr-*cis*-TFSA-Li_2S_8	C-H···S	1	-34.0	C_4mpyr-*trans*-TFSA-Li_2S_8	F···S	1	-41.3
	C-H···S	2	-23.7		N···S	2	-102.4
	C-H···S	3	-36.8		O···Li	3	-372.0
	C-H···S	4	-64.9		O···Li	4	-330.8
	C-H···S	5	-47.8		N···S	5	-91.2
	C-H···S	6	-85.3		C-H···S	6	-90.0
	C-H···S	7	-71.2		C-H···S	7	-40.1
	C-H···S	8	-17.8		C-H···S	8	-117.7
	C-H···S	9	-52.2		C-H···O	9	-108.0
	O···S	10	-115.2		F···S	10	-23.1
	O···Li	11	-386.5		C-H···O	11	-32.4
	O···Li	12	-408.2		C-H···F	12	-65.5

	F···S	13	-41.9		C-H···O	13	-67.5
	F···S	14	-41.6		C-H···O	14	-143.6
	C-H···O	15	-69.5				
	C-H···O	16	-90.0				
	C-H···N	17	-107.5				
	C-H···O	18	-128.9				
	C-H···O	19	-139.2				
C_4mpyr-Li_2S_8-*cis*-TFSA	O···Li	1	-432.1	C_4mpyr-Li_2S_8-*trans*-TFSA	F···S	1	-38.7
	O···S	2	-103.8		O···S	2	-126.7
	N··S	3	-89.5		O···Li	3	-464.2
	N··S	4	-88.6		O···S	4	-128.2
	N···Li	5	-252.9		O···Li	5	-508.9
	O···Li	6	-358.0		C-H···S	6	-56.3
	C-H···O	7	-118.8		C-H···S	7	-24.4
	C-H···F	8	-49.3		C-H···S	8	-80.2
	C-H···F	9	-62.5		C-H···S	9	-33.2
	C-H···S	10	-111.0		C-H···S	10	-84.2
	C-H···S	11	-71.6		C-H···O	11	-151.2
	C-H···S	12	-52.4		C-H···S	12	-85.8
	C-H···S	13	-60.1		C-H···O	13	-62.0
	C-H···S	14	-55.8		F···S	14	-32.7
	C-H···S	15	-66.9		C-H···F	15	-61.0
	C-H···S	16	-30.3		C-H···O	16	-111.6
					C-H···O	17	-103.9
Li_2S_8-C_4mpyr-*cis*-TFSA	C-H···S	1	-59.3	Li_2S_8-C_4mpyr-*trans*-TFSA	O···S	1	-27.2
	C-H···S	2	-64.6		F···S	2	-20.7
	C-H···S	3	-71.7		O···S	3	-70.3
	C-H···S	4	-30.8		O···S	4	-55.9
	C-H···S	5	-34.0		F···S	5	-30.8
	C-H···S	6	-72.6		O···S	6	-52.8
	C-H···S	7	-49.6		O···Li	7	-437.4
	O···S	8	-109.2		C-H···S	8	-67.4
	O···S	9	-59.8		C-H···S	9	-80.7
	O···Li	10	-393.0		C-H···S	10	-29.2
	O···Li	11	-379.9		C-H···S	11	-109.0
	F···S	12	-16.6		C-H···S	12	-67.7
	C-H···O	13	-58.4		O···Li	13	-427.9
	C-H···O	14	-121.1		C-H···O	14	-21.8
	C-H···O	15	-36.3		C-H···F	15	-71.3
	C-H···O	16	-59.3		C-H···O	16	-143.0
	C-H···O	17	-102.5		C-H···O	17	-47.0
	C-H···O	18	-95.3		C-H···O	18	-141.7

	C-H···N	19	-68.5		C-H···F	19	-34.7
	C-H···O	20	-96.8				
Li_2S_8-co-C_4mpyr-*cis*-TFSA	C-H···S	1	-63.8	Li_2S_8-co-C_4mpyr-*trans*-TFSA	C-H···S	1	-32.5
	C-H···S	2	-41.8		C-H···S	2	-45.8
	C-H···S	3	-31.6		C-H···S	3	-36.6
	C-H···S	4	-42.7		C-H···S	4	-40.6
	C-H···S	5	-81.4		C-H···S	5	-54.6
	C-H···S	6	-40.5		C-H···S	6	-32.1
	C-H···S	7	-72.9		O···Li	7	-518.5
	C-H···S	8	-33.0		O···S	8	-56.6
	C-H···S	9	-57.1		C-H···F	9	-27.1
	O···Li	10	-381.1		C-H···O	10	-160.6
	N···Li	11	-161.8		C-H···O	11	-187.7
	C-H···S	12	-97.3		C-H···F	12	-30.1
	C-H···N	13	-65.4		C-H···F	13	-20.5
	C-H···O	14	-91.8		C-H···O	14	-144.6
	C-H···O	15	-96.8		C-H···O	15	-98.9
	C-H···O	16	-134.3				
	C-H···O	17	-46.9				
	C-H···O	18	-112.6				
	C-H···S	19	-34.2				
C_4mpyr-*cis*-TFSA-S_8	F···S	1	-70.6	C_4mpyr-*trans*-TFSA-S_8	F···S	1	-37.9
	F···S	2	-70.8		F···S	2	-60.2
	F···S	3	-18.0		F···S	3	-34.8
	N··S	4	-12.3		F···S	4	-25.6
	F···S	5	-53.7		O···S	5	-97.3
	F···S	6	-12.3		N···S	6	-80.2
	F···S	7	-71.4		O···S	7	-47.9
	F···S	8	-34.1		C-H···S	8	-49.6
	F···S	9	-65.4		C-H···S	9	-42.5
	C-H···O	10	-104.9		C-H···S	10	-56.9
	C-H···O	11	-36.4		C-H···S	11	-27.7
	C-H···O	12	-88.4		C-H···F	12	-64.5
	C-H···O	13	-175.0		C-H···F	13	-46.8
	C-H···N	14	-76.8		C-H···N	14	-28.0
	C-H···O	15	-78.7		C-H···F	15	-49.6
	C-H···O	16	-63.4		C-H···F	16	-65.3
	C-H···O	17	-163.2		C-H···O	17	-202.5
	C-H···O	18	-106.5		C-H···N	18	-93.2
	C-H···O	19	-66.5		C-H···O	19	-135.1
					C-H···O	20	-49.5

C_4mpyr-S_8-*cis*-TFSA	C-H···S	1	-69.1	C_4mpyr-S_8-*trans*-TFSA	F···S	1	-70.7
	C-H···S	2	-43.1		N···S	2	-94.6
	C-H···S	3	-51.6		N···S	3	-153.1
	C-H···S	4	-67.1		O···S	4	-87.7
	C-H···S	5	-31.4		O···S	5	-59.0
	C-H···S	6	-27.5		O···S	6	-70.5
	C-H···S	7	-67.3		O···S	7	-19.7
	C-H···S	8	-54.6		C-H···S	8	-72.5
	C-H···S	9	-62.0		C-H···S	9	-46.0
	O···S	10	-105.5		C-H···S	10	-89.9
	F···S	11	-60.9		C-H···S	11	-35.2
	O···S	12	-97.9		C-H···S	12	-70.8
	F···S	13	-30.1		C-H···S	13	-47.9
	O···S	14	-77.7		C-H···S	14	-55.5
	O···S	15	-133.0		C-H···S	15	-49.9
S_8-C_4mpyr-*cis*-TFSA	N···S	1	-79.0	S_8-C_4mpyr-*trans*-TFSA	C-H···S	1	-62.1
	O···S	2	-66.0		C-H···S	2	-62.1
	O···S	3	-97.3		C-H···S	3	-39.0
	C-H···O	4	-30.5		C-H···S	4	-28.0
	C-H···O	5	-83.1		C-H···S	5	-46.2
	C-H···S	6	-64.5		C-H···S	6	-42.1
	C-H···S	7	-53.5		O···S	7	-117.2
	C-H···S	8	-55.2		C-H···S	8	-81.8
	C-H···S	9	-39.9		O···S	9	-71.1
	C-H···N	10	-102.5		C-H···F	10	-3.5
	C-H···O	11	-135.9		C-H···O	11	-48.2
	C-H···O	12	-146.9		C-H···O	12	-159.6
	C-H···O	13	-92.5		C-H···O	13	-98.1
	C-H···O	14	-53.3		C-H···O	14	-70.6
	C-H···O	15	-65.8		C-H···O	15	-160.7
					C-H···O	16	-137.7
					C-H···O	17	-110.6
					C-H···F	18	-22.2
S_8-co-C_4mpyr-*cis*-TFSA	F···S	1	-17.3	S_8-co-C_4mpyr-*trans*-TFSA	F···S	1	-22.8
	O···S	2	-82.8		O···S	2	-115.1
	N···S	3	-91.8		F···S	3	-26.3
	O···S	4	-73.4		O···S	4	-73.9
	C-H···S	5	-56.6		F···S	5	-24.4
	C-H···S	6	-55.8		C-H···S	6	-44.6
	C-H···O	7	-47.3		C-H···S	7	-55.1
	C-H···O	8	-53.6		C-H···S	8	-83.8
	C-H···N	9	-81.0		C-H···S	9	-31.0

	C-H···O	10	-52.7		C-H···S	10	-33.8
	C-H···O	11	-100.7		C-H···S	11	-60.1
	C-H···O	12	-112.1		C-H···S	12	-29.4
	C-H···O	13	-75.3		C-H···S	13	-46.1
	C-H···O	14	-164.4		C-H···S	14	-32.4
	C-H···O	15	-63.9		C-H···O	15	-157.2
	C-H···O	16	-61.3		C-H···O	16	-113.5
	C-H···S	17	-26.7		C-H···O	17	-65.7
	C-H···S	18	-42.9		C-H···O	18	-106.0
	C-H···S	19	-34.4				
	C-H···S	20	-38.5				
	C-H···S	21	-57.2				
	C-H···S	22	-27.8				
C_4mpyr-*cis*-FSA-Li_2S_1	C-H···S	1	-88.3	C_4mpyr-*trans*-FSA-Li_2S_1	C-H···O	1	-55.8
	C-H···S	2	-167.1		C-H···O	2	-49.4
	C-H···S	3	-125.6		C-H···O	3	-54.7
	O···Li	4	-428.5		C-H···O	4	-73.1
	O···S	5	-96.5		C-H···O	5	-121.2
	O···Li	6	-317.2		C-H···S	6	-77.7
	O···Li	7	-278.5		C-H···S	7	-167.4
	C-H···O	8	-124.6		C-H···S	8	-132.4
	C-H···N	9	-71.2		O···Li	9	-255.9
	C-H···O	10	-58.5		O···Li	10	-351.3
	C-H···O	11	-67.6		F···Li	11	-466.1
C_4mpyr-Li_2S_1-*cis*-FSA	C-H···F	1	-115.0	C_4mpyr-Li_2S_1-*trans*-FSA	C-H···O	1	-47.6
	C-H···F	2	-57.2		C-H···O	2	-43.4
	O···Li	3	-507.4		C-H···O	3	-29.3
	O···Li	4	-491.2		C-H···F	4	-56.8
	C-H···S	5	-129.6		C-H···F	5	-89.4
	C-H···S	6	-132.8		O···Li	6	-482.6
	C-H···S	7	-118.7		O···Li	7	-457.0
					C-H···S	8	-95.2
					C-H···S	9	-136.2
					C-H···S	10	-144.8
Li_2S_1-C_4mpyr-*cis*-FSA	O···Li	1	-321.8	Li_2S_1-C_4mpyr-*trans*-FSA	C-H···S	1	-121.7
	N···Li	2	-290.9		C-H···S	2	-127.7
	C-H···S	3	-108.2		C-H···S	3	-112.7
	C-H···S	4	-134.5		O···Li	4	-386.6
	C-H···S	5	-125.9		F···Li	5	-368.9
	C-H···O	6	-125.1		C-H···O	6	-47.9
	C-H···O	7	-113.8		C-H···O	7	-42.8

	C-H···N	8	-35.3		C-H···O	8	-70.2
	C-H···N	9	-26.5		C-H···O	9	-56.3
	C-H···O	10	-36.9		C-H···O	10	-30.3
	C-H···O	11	-40.7		C-H···F	11	-99.4
					C-H···F	12	-86.0
Li_2S_1-co-C_4mpyr-*cis*-FSA	O···Li	1	-476.1	Li_2S_1-co-C_4mpyr-*trans*-FSA	C-H···S	1	-138.7
	O···Li	2	-483.6		C-H···S	2	-154.4
	C-H···O	3	-55.4		O···Li	3	-430.2
	C-H···N	4	-87.8		C-H···S	4	-105.3
	C-H···O	5	-64.1		N···Li	5	-282.4
	C-H···O	6	-61.8		O···Li	6	-243.5
	C-H···O	7	-50.2		C-H···O	7	-28.3
	C-H···S	8	-153.1		C-H···O	8	-38.7
	C-H···S	9	-71.2				
	C-H···S	10	-128.6				
C_4mpyr-*cis*-FSA-Li_2S_2	O···Li	1	-223.1	C_4mpyr-*trans*-FSA-Li_2S_2	C-H···F	1	-17.1
	O···Li	2	-226.8		C-H···O	2	-38.5
	O···S	3	-85.5		C-H···O	3	-133.6
	C-H···Li	4	-64.3		C-H···O	4	-129.0
	C-H···S	5	-44.4		C-H···O	5	-152.9
	O···Li	6	-348.8		C-H···F	6	-96.5
	C-H···O	7	-35.8		C-H···F	7	-165.8
	C-H···S	8	-132.0		O···Li	8	-322.3
	C-H···O	9	-55.4		O···Li	9	-304.6
	C-H···S	10	-126.1		N···S	10	-66.9
	C-H···N	11	-61.2		F···S	11	-46.4
	C-H···N	12	-135.7				
	C-H···O	13	-100.3				
	C-H···O	14	-101.4				
C_4mpyr-Li_2S_2-*cis*-FSA	O···Li	1	-377.1	C_4mpyr-Li_2S_2-*trans*-FSA	C-H···F	1	-32.2
	O···S	2	-127.9		C-H···O	2	-40.7
	O···Li	3	-282.7		C-H···O	3	-51.4
	O···Li	4	-476.9		C-H···S	4	-79.8
	C-H···O	5	-30.3		C-H···S	5	-143.7
	C-H···O	6	-76.8		C-H···S	6	-144.5
	C-H···S	7	-30.5		O···Li	7	-458.4
	C-H···S	8	-45.6		O···Li	8	-251.4
	C-H···S	9	-105.5		O···S	9	-121.5
	C-H···S	10	-95.4		O···S	10	-119.6
	C-H···S	11	-132.2		O···Li	11	-376.7
	C-H···S	12	-104.5				

Li_2S_2-C_4mpyr-*cis*-FSA	O···Li	1	-329.9	Li_2S_2-C_4mpyr-*trans*-FSA	O···Li	1	-548.4
	O···S	2	-132.9		C-H···N	2	-141.8
	O···Li	3	-319.9		C-H···N	3	-46.0
	O···Li	4	-482.3		C-H···S	4	-61.6
	C-H···O	5	-44.6		C-H···S	5	-79.6
	C-H···O	6	-61.1		C-H···S	6	-97.5
	C-H···O	7	-70.4		C-H···S	7	-95.1
	C-H···S	8	-32.6		C-H···F	8	-66.6
	C-H···S	9	-107.8		C-H···O	9	-147.8
	C-H···S	10	-133.3		C-H···O	10	-103.3
	C-H···S	11	-40.3				
	C-H···S	12	-90.0				
	C-H···S	13	-53.2				
Li_2S_2-co-C_4mpyr-*cis*-FSA	O···Li	1	-257.6	Li_2S_2-co-C_4mpyr-*trans*-FSA	C-H···S	1	-122.2
	N···Li	2	-183.3		C-H···S	2	-149.3
	N···S	3	-77.4		O···Li	3	-391.0
	O···Li	4	-390.8		N···S	4	-79.1
	C-H···S	5	-25.3		O···Li	5	-227.9
	C-H···S	6	-120.8		N···Li	6	-225.2
	C-H···S	7	-111.6		C-H···N	7	-56.5
	C-H···O	8	-70.3		C-H···O	8	-50.4
	C-H···O	9	-142.5		C-H···O	9	-69.8
	C-H···O	10	-61.6		C-H···O	10	-54.7
	C-H···O	11	-76.3		C-H···F	11	-58.5
	C-H···O	12	-95.5		C-H···O	12	-68.5
					C-H···F	13	-106.5
					C-H···F	14	-41.9
					C-H···F	15	-48.0
C_4mpyr-*cis*-FSA-Li_2S_4	C-H···S	1	-83.3	C_4mpyr-*trans*-FSA-Li_2S_4	O···Li	1	-261.2
	C-H···S	2	-120.5		C-H···O	2	-112.1
	C-H···S	3	-44.2		F···S	2	-79.8
	O···S	4	-40.4		N···S	3	-73.3
	O···S	5	-79.8		N···S	4	-57.4
	O···Li	6	-362.0		O···Li	5	-292.5
	O···Li	7	-348.6		C-H···O	6	-88.2
	C-H···O	8	-70.6		C-H···F	7	-92.7
	C-H···O	9	-53.5		C-H···O	8	-114.2
	C-H···N	10	-63.5		C-H···F	9	-168.2
	C-H···O	11	-111.8		C-H···O	10	-76.9
	C-H···N	12	-61.5		C-H···O	11	-81.4
	C-H···O	13	-122.2				

	C-H···O	14	-49.8				
C_4mpyr-Li_2S_4-*cis*-FSA	O···Li	1	-291.2	C_4mpyr-Li_2S_4-*trans*-FSA	C-H···S	1	-104.5
	O···S	2	-120.0		C-H···S	2	-68.1
	O···S	3	-127.3		C-H···S	3	-81.0
	O···Li	4	-314.2		C-H···S	4	-43.9
	O···Li	5	-487.3		C-H···S	5	-111.7
	C-H···S	6	-37.3		O···Li	6	-458.3
	C-H···S	7	-60.6		C-H···O	7	-61.3
	C-H···S	8	-112.8		C-H···O	8	-124.9
	C-H···S	9	-88.7		C-H···F	9	-33.6
	C-H···S	10	-60.7		O···Li	10	-298.1
	C-H···S	11	-79.0		O···S	11	-121.6
	C-H···O	12	-74.7		O···S	12	-125.4
	C-H···O	13	-50.0		O···Li	13	-259.1
	C-H···O	14	-45.1				
	C-H···O	15	-54.4				
	C-H···O	16	-53.6				
Li_2S_4-C_4mpyr-*cis*-FSA	C-H···O	1	-133.9	Li_2S_4-C_4mpyr-*trans*-FSA	C-H···S	1	-67.9
	C-H···O	2	-141.2		C-H···S	2	-68.3
	C-H···O	3	-142.1		C-H···S	3	-61.1
	C-H···O	4	-124.3		C-H···S	4	-76.0
	C-H···N	5	-72.2		C-H···S	5	-67.0
	C-H···O	6	-83.8		F···S	6	-42.3
	C-H···O	7	-91.3		O···Li	7	-369.5
	C-H···O	8	-69.1		O···Li	8	-377.6
	C-H···S	9	-87.4		C-H···F	9	-72.7
	C-H···S	10	-58.9		C-H···O	10	-92.7
	C-H···S	11	-44.3		C-H···O	11	-70.9
	C-H···S	12	-53.7		C-H···O	12	-57.5
	C-H···S	13	-57.0		C-H···O	13	-50.4
	C-H···Li	14	-134.3		C-H···O	14	-125.9
	C-H···S	15	-51.8				
Li_2S_4-co-C_4mpyr-*cis*-FSA	C-H···S	1	-83.2	Li_2S_4-co-C_4mpyr-*trans*-FSA	O···Li	1	-453.3
	C-H···S	2	-120.5		C-H···S	2	-50.9
	C-H···S	3	-44.3		C-H···Li	3	-82.4
	O···S	4	-40.4		C-H···O	4	-66.1
	O···S	5	-79.8		C-H···O	5	-89.6
	O···Li	6	-348.2		C-H···O	6	-81.8
	O···Li	7	-362.0		C-H···S	7	-117.3
	C-H···O	8	-53.8		C-H···S	8	-68.3
	C-H···O	9	-70.6		C-H···O	9	-61.0

	C-H···N	10	-63.4		C-H···N	10	-131.3
	C-H···O	11	-111.8		C-H···N	11	-93.7
	C-H···N	12	-61.7		C-H···N	12	-49.9
	C-H···O	13	-122.1		C-H···F	13	-70.3
	C-H···O	14	-49.8		C-H···O	14	-109.4
					C-H···O	15	-89.0
C_4mpyr-*cis*-FSA-Li_2S_6	C-H···S	1	-49.4	C_4mpyr-*trans*-FSA-Li_2S_6	C-H···F	1	-33.3
	C-H···S	2	-43.0		C-H···F	2	-112.8
	C-H···S	3	-60.9		C-H···O	3	-118.4
	C-H···S	4	-65.3		C-H···O	4	-88.8
	C-H···S	5	-44.6		C-H···O	5	-160.0
	O···S	6	-43.2		C-H···O	6	-44.5
	O···S	7	-26.9		C-H···O	7	-103.1
	N···Li	8	-410.0		C-H···F	8	-27.3
	O···S	9	-52.4		N···Li	9	-278.6
	C-H···O	10	-20.7		N···S	10	-79.9
	C-H···O	11	-81.8		O···S	11	-88.6
	C-H···N	12	-50.0		O···Li	12	-324.5
	C-H···O	13	-66.7				
	C-H···O	14	-90.8				
	C-H···O	15	-131.4				
	C-H···O	16	-68.0				
	C-H···O	17	-102.2				
C_4mpyr-Li_2S_6-*cis*-FSA	O···Li	1	-394.9	C_4mpyr-Li_2S_6-*trans*-FSA	C-H···S	1	-48.7
	O···Li	2	-465.8		C-H···S	2	-84.6
	O···S	3	-77.3		C-H···S	3	-55.4
	O···S	4	-18.9		C-H···S	4	-83.7
	C-H···S	5	-38.9		C-H···S	5	-117.5
	C-H···S	6	-44.0		F···S	6	-65.8
	C-H···S	7	-80.3		O···S	7	-88.5
	C-H···S	8	-91.9		O···Li	8	-366.8
	C-H···S	9	-95.3		O···S	9	-79.3
	C-H···S	10	-70.2		F···S	10	-81.1
	C-H···N	11	-122.1		F···Li	11	-232.0
	C-H···O	12	-149.5		O···Li	12	-371.9
	C-H···O	13	-63.1		C-H···O	13	-50.3
	C-H···O	14	-52.9		C-H···F	14	-64.1
					C-H···F	15	-97.4
					C-H···O	16	-44.3
					C-H···O	17	-29.5
					C-H···O	18	-17.5

Li_2S_6-C_4mpyr-*cis*-FSA	O···S	1	-78.5	Li_2S_6-C_4mpyr-*trans*-FSA	C-H···S	1	-38.4
	N···Li	2	-397.9		C-H···S	2	-45.9
	C-H···N	3	-86.0		C-H···S	3	-77.3
	O···S	4	-58.8		C-H···S	4	-52.7
	C-H···S	5	-51.0		C-H···S	5	-32.8
	C-H···S	6	-23.1		C-H···S	6	-48.9
	C-H···S	7	-68.0		O···S	7	-81.4
	C-H···O	8	-36.5		O···Li	8	-373.0
	C-H···O	9	-58.9		O···Li	9	-355.9
	C-H···S	10	-28.1		O···S	10	-77.3
	C-H···S	11	-76.5		C-H···S	11	-62.5
	C-H···O	12	-83.7		C-H···O	12	-110.3
	C-H···O	13	-119.1		C-H···F	13	-119.0
	C-H···O	15	-81.0		C-H···F	14	-130.4
	C-H···O	16	-100.0				
Li_2S_6-co-C_4mpyr-*cis*-FSA	O···Li	1	-613.4	Li_2S_6-co-C_4mpyr-*trans*-FSA	O···S	1	-112.1
	C-H···S	2	-49.4		O···Li	2	-344.9
	C-H···S	3	-61.7		O···Li	3	-261.4
	C-H···S	4	-23.3		O···Li	4	-429.4
	C-H···S	5	-57.7		O···S	5	-91.9
	C-H···S	6	-69.2		C-H···S	6	-79.0
	C-H···S	7	-63.4		C-H···O	7	-91.2
	C-H···O	8	-49.9		C-H···S	8	-91.3
	C-H···O	9	-75.6		C-H···S	9	-30.6
	C-H···N	10	-96.6		F···S	10	-34.5
	C-H···O	11	-91.5		C-H···F	11	-126.3
	C-H···O	12	-143.5		C-H···F	12	-64.3
					C-H···F	13	-96.6
					C-H···O	14	-21.5
					C-H···S	15	-100.8
					C-H···S	16	-84.4
C_4mpyr-*cis*-FSA-Li_2S_8	C-H···O	1	-53.1	C_4mpyr-*trans*-FSA-Li_2S_8	C-H···S	1	-71.4
	C-H···O	2	-108.9		C-H···S	2	-134.7
	C-H···N	3	-39.7		C-H···S	3	-71.3
	C-H···N	4	-106.1		O···S	4	-30.9
	C-H···N	5	-145.6		N···S	5	-76.1
	C-H···S	6	-67.7		O···S	6	-120.5
	C-H···O	7	-85.5		O···Li	7	-361.4
	C-H···O	8	-108.3		N···Li	8	-254.8
	O···Li	9	-325.9		N···S	9	-110.8
	O···Li	10	-341.8		C-H···O	10	-33.7
	O···S	11	-47.1		C-H···O	11	-52.4

	O···S	12	-61.9		C-H···F	12	-135.5
	O···S	13	-52.3		C-H···O	13	-76.3
	O···S	14	-35.3		C-H···F	14	-123.1
	N···S	15	-52.0		C-H···F	15	-68.3
C_4mpyr-Li_2S_8-*cis*-FSA	O···Li	1	-430.6	C_4mpyr-Li_2S_8-*trans*-FSA	F···S	1	-63.6
	O···Li	2	-212.7		O···S	2	-108.6
	O···S	3	-110.8		O···Li	3	-449.4
	O···S	4	-99.0		O···Li	4	-423.7
	O···Li	5	-333.0		O···S	5	-88.1
	O···S	6	-140.1		C-H···O	6	-101.5
	C-H···O	7	-75.3		C-H···F	7	-92.1
	C-H···N	8	-34.9		F···S	8	-76.9
	C-H···N	9	-73.3		C-H···O	9	-133.5
	C-H···O	10	-90.3		C-H···S	10	-31.8
	C-H···S	11	-76.0		C-H···S	11	-100.4
	C-H···O	12	-120.9		C-H···S	12	-34.6
	C-H···S	13	-98.2		C-H···S	13	-43.5
	C-H···S	14	-103.4		C-H···S	14	-95.8
	C-H···S	15	-30.6		C-H···S	15	-47.8
	C-H···S	16	-60.0				
	C-H···S	17	-30.8				
	C-H···S	18	-47.0				
Li_2S_8-C_4mpyr-*cis*-FSA	C-H···S	1	-40.4	Li_2S_8-C_4mpyr-*trans*-FSA	O···S	1	-72.2
	C-H···S	2	-31.7		O···Li	2	-493.5
	C-H···S	3	-30.3		O···S	3	-27.0
	C-H···S	4	-33.6		F···S	4	-22.8
	C-H···S	5	-73.0		C-H···S	5	-69.7
	C-H···S	6	-60.3		C-H···S	6	-78.6
	C-H···S	7	-51.4		C-H···S	7	-80.0
	O···S	8	-78.9		C-H···S	8	-81.5
	O···Li	9	-402.8		C-H···N	9	-132.9
	O···Li	10	-425.1		C-H···F	10	-96.1
	O···S	11	-78.2		C-H···O	11	-122.5
	O···S	12	-33.2		C-H···O	12	-153.0
	C-H···O	13	-60.5		C-H···O	13	-64.6
	C-H···S	14	-67.7				
	C-H···O	15	-65.2				
	C-H···N	16	-96.2				
	C-H···N	17	-46.8				
	C-H···O	18	-79.2				
Li_2S_8-co-C_4mpyr-*cis*-FSA	C-H···S	1	-46.0	Li_2S_8-co-C_4mpyr-*trans*-FSA	C-H···S	1	-87.2

	C-H···S	2	-30.9		C-H···S	2	-34.0
	C-H···S	3	-29.3		C-H···S	3	-51.1
	C-H···S	4	-35.7		C-H···S	4	-35.5
	C-H···S	5	-49.3		C-H···S	5	-66.0
	C-H···S	6	-116.1		C-H···S	6	-46.9
	C-H···S	7	-76.1		C-H···S	7	-44.3
	C-H···S	8	-35.9		C-H···S	8	-36.1
	O···S	9	-81.2		O···Li	9	-556.2
	O···S	10	-81.3		C-H···O	10	-33.4
	O···Li	11	-353.6		C-H···O	11	-133.6
	O···Li	12	-332.6		C-H···F	12	-35.4
	C-H···O	13	-68.0		C-H···F	13	-47.8
	C-H···O	14	-118.4		C-H···O	14	-124.1
	C-H···O	15	-50.3		C-H···O	15	-69.2
	C-H···N	16	-81.1		C-H···O	16	-183.7
	C-H···O	17	-100.0				
	C-H···N	18	-98.9				
	C-H···O	19	-78.7				
C_4mpyr-*cis*-FSA-S_8	C-H···S	1	-42.0	C_4mpyr-*trans*-FSA-S_8	O···S	1	-81.9
	C-H···S	2	-44.7		O···S	2	-45.4
	C-H···S	3	-43.3		O···S	3	-60.9
	C-H···S	4	-23.7		O···S	4	-69.3
	C-H···S	5	-68.8		O···S	5	-40.5
	C-H···S	6	-47.4		F···S	6	-51.7
	C-H···S	7	-20.1		C-H···S	7	-72.9
	C-H···S	8	-49.9		O···S	8	-39.3
	C-H···S	9	-47.1		C-H···S	9	-19.4
	O···S	10	-105.3		C-H···S	10	-31.1
	N···S	11	-106.4		C-H···S	11	-48.3
	O···S	12	-78.1		C-H···F	12	-39.9
	C-H···N	13	-94.5		C-H···S	13	-42.2
	C-H···O	14	-47.8		C-H···O	14	-104.6
	C-H···O	15	-66.4		C-H···O	15	-228.9
	C-H···O	16	-29.8		C-H···O	16	-97.4
	C-H···O	17	-100.3		C-H···O	17	-149.8
	C-H···O	18	-87.0		C-H···F	18	-130.1
	C-H···O	19	-113.1		C-H···F	19	-61.9
	C-H···O	20	-155.7				
C_4mpyr-S_8-*cis*-FSA	C-H···S	1	-50.7	C_4mpyr-S_8-*trans*-FSA	F···S	1	-103.3
	C-H···S	2	-33.9		F···S	2	-128.3
	C-H···S	3	-72.4		O···S	3	-69.4
	C-H···S	4	-69.8		O···S	4	-88.5

	C-H···S	5	-55.8		O···S	5	-84.3
	C-H···S	6	-59.4		O···S	6	-68.5
	C-H···S	7	-52.0		O···S	7	-69.2
	C-H···S	8	-84.7		C-H···S	8	-42.6
	O···S	9	-69.6		C-H···S	9	-78.6
	O···S	10	-75.9		C-H···S	10	-65.1
	O···S	11	-112.0		C-H···S	11	-68.7
	N···S	12	-59.8		C-H···S	12	-56.4
	O···S	13	-72.4		C-H···S	13	-84.4
	O···S	14	-103.6		C-H···S	14	-51.9
	O···S	15	-62.0				
	O···S	16	-92.3				
S_8-C_4mpyr-*cis*-FSA	O···S	1	-103.8	S_8-C_4mpyr-*trans*-FSA	C-H···S	1	-60.5
	O···S	2	-105.8		C-H···S	2	-33.7
	C-H···S	3	-38.1		C-H···S	3	-30.9
	C-H···O	4	-47.0		C-H···S	4	-55.1
	C-H···O	5	-46.3		C-H···S	5	-57.8
	C-H···O	6	-100.5		C-H···S	6	-28.8
	C-H···N	7	-113.5		C-H···S	7	-29.3
	C-H···O	8	-61.6		C-H···S	8	-73.9
	C-H···O	9	-96.1		C-H···S	9	-40.0
	C-H···N	10	-31.8		O···S	10	-111.3
	C-H···N	11	-82.8		F···S	11	-76.6
	C-H···O	12	-107.9		C-H···O	12	-133.3
	C-H···O	13	-143.0		C-H···O	13	-167.8
	C-H···S	14	-53.1		C-H···O	14	-82.6
	C-H···S	15	-23.5		C-H···O	15	-128.6
	C-H···S	16	-76.0		C-H···F	16	-53.7
	C-H···S	17	-41.8		C-H···F	17	-120.0
	C-H···S	18	-54.3				
	C-H···S	19	-36.8				
	C-H···S	20	-62.0				
S_8-co-C_4mpyr-*cis*-FSA	O···S	1	-104.9	S_8-co-C_4mpyr-*trans*-FSA	O···S	1	-27.5
	O···S	2	-90.7		O···S	2	-62.4
	C-H···O	3	-24.9		C-H···S	3	-36.7
	C-H···S	4	-78.2		C-H···O	4	-204.5
	C-H···S	5	-37.7		C-H···S	5	-83.3
	C-H···O	6	-119.9		C-H···S	6	-25.2
	C-H···N	7	-111.3		C-H···O	7	-98.5
	C-H···O	8	-126.8		C-H···F	8	-46.5
	C-H···N	9	-160.2		C-H···O	10	-170.1
	C-H···O	10	-115.8		C-H···F	11	-31.5

	C-H···S	11	-30.3		C-H···O	12	-107.4
	C-H···S	12	-47.0		C-H···S	13	-61.6
	C-H···S	13	-27.7		C-H···S	14	-50.6
	C-H···S	14	-35.8		C-H···S	15	-15.6
	C-H···S	15	-71.3		C-H···S	16	-43.0
	C-H···S	16	-40.3		C-H···F	19	-104.3
C_4mpyr-*cis*-FTFSA-Li_2S_1	C-H···S	1	-92.0	C_4mpyr-*trans*-FTFSA-Li_2S_1	C-H···F	1	-22.4
	C-H···S	2	-171.4		C-H···O	2	-24.7
	C-H···S	3	-125.2		C-H···F	3	-45.9
	O···Li	4	-435.8		C-H···O	4	-127.6
	O···S	5	-94.9		C-H···N	5	-103.4
	O···Li	6	-269.5		C-H···N	6	-154.8
	C-H···N	7	-69.2		C-H···O	7	-99.9
	C-H···O	8	-69.1		C-H···O	8	-104.7
	C-H···O	9	-136.9		F···S	9	-32.8
	C-H···O	10	-75.1		O···Li	10	-394.0
					O···Li	11	-389.2
C_4mpyr-Li_2S_1-*cis*-FTFSA	C-H···O	1	-39.3	C_4mpyr-Li_2S_1-*trans*-FTFSA	C-H···O	1	-47.9
	C-H···O	2	-37.9		C-H···F	2	-56.1
	O···Li	3	-274.9		C-H···F	3	-124.8
	C-H···O	4	-55.4		C-H···S	4	-104.0
	N···Li	5	-256.8		C-H···S	5	-134.5
	C-H···F	6	-63.0		C-H···S	6	-135.0
	C-H···S	7	-127.1		O···Li	7	-479.6
	C-H···S	8	-129.7		O···Li	8	-469.9
	C-H···S	9	-137.3				
	C-H···F	10	-42.1				
	O···Li	11	-436.9				
Li_2S_1-C_4mpyr-*cis*-FTFSA	C-H···S	1	-130.0	Li_2S_1-C_4mpyr-*trans*-FTFSA	C-H···S	1	-134.5
	C-H···S	2	-37.0		C-H···S	2	-30.2
	C-H···S	3	-115.4		C-H···S	3	-140.3
	O···Li	4	-533.7		O···Li	4	-551.6
	C-H···O	5	-97.6		C-H···O	5	-105.9
	C-H···O	6	-79.9		C-H···F	6	-47.0
	C-H···O	7	-116.9		C-H···O	7	-54.0
	C-H···O	8	-49.2		C-H···F	8	-49.1
	C-H···O	9	-69.9		C-H···O	9	-69.4
	C-H···O	10	-134.8		C-H···F	10	-64.9
					C-H···O	11	-171.3
					C-H···O	12	-104.8

Li_2S_1-co-C_4mpyr-*cis*-FTFSA	O···Li	1	-477.1	Li_2S_1-co-C_4mpyr-*trans*-FTFSA	O···Li	1	-431.8
	O···Li	2	-485.4		N···Li	2	-251.4
	C-H···S	3	-130.5		O···Li	3	-289.4
	C-H···S	4	-67.5		C-H···S	4	-108.4
	C-H···S	5	-151.6		C-H···S	5	-142.6
	C-H···O	6	-61.7		C-H···S	6	-140.2
	C-H···O	7	-67.8		C-H···O	7	-56.3
	C-H···O	8	-61.4				
	C-H···N	9	-84.0				
	C-H···O	10	-71.0				
C_4mpyr-*cis*-FTFSA-Li_2S_2	O···Li	1	-215.7	C_4mpyr-*trans*-FTFSA-Li_2S_2	C-H···F	1	-37.2
	O···Li	2	-248.9		C-H···F	2	-58.8
	C-H···S	3	-38.6		C-H···O	3	-130.1
	C-H···Li	4	-56.6		C-H···O	4	-49.0
	O···Li	5	-350.3		C-H···O	5	-142.2
	C-H···S	6	-132.8		C-H···O	6	-167.1
	C-H···S	7	-129.4		C-H···O	7	-102.5
	C-H···O	8	-36.8		C-H···F	8	-64.7
	C-H···N	9	-42.5		N···Li	9	-252.6
	C-H···O	10	-64.5		O···Li	10	-367.0
	C-H···O	11	-105.8		F···S	11	-25.3
	C-H···N	12	-137.8				
	C-H···O	13	-108.7				
C_4mpyr-Li_2S_2-*cis*-FTFSA	C-H···O	1	-31.9	C_4mpyr-Li_2S_2-*trans*-FTFSA	C-H···S	1	-77.8
	C-H···O	2	-71.2		C-H···S	2	-51.3
	C-H···S	3	-33.2		C-H···S	3	-136.4
	C-H···S	4	-47.3		C-H···S	4	-135.8
	C-H···S	5	-108.7		O···S	5	-122.4
	O···Li	6	-484.3		O···S	6	-124.3
	C-H···S	7	-135.7		O···Li	7	-343.7
	C-H···S	8	-102.1		O···Li	8	-316.2
	O···Li	9	-295.5		O···Li	9	-472.1
	O···S	10	-130.1		C-H···O	10	-100.3
	O···Li	11	-379.1		C-H···O	11	-101.7
Li_2S_2-C_4mpyr-*cis*-FTFSA	C-H···O	1	-56.8	Li_2S_2-C_4mpyr-*trans*-FTFSA	C-H···S	1	-68.7
	C-H···O	2	-68.3		C-H···S	2	-117.4
	C-H···O	3	-108.3		C-H···S	3	-73.9
	C-H···O	4	-107.3		O···Li	4	-538.0
	C-H···N	5	-60.7		C-H···O	5	-130.2
	C-H···N	6	-147.0		C-H···O	6	-73.4
	C-H···O	7	-121.8		C-H···O	7	-102.0

	C-H···O	8	-111.4		C-H···O	8	-42.3
	C-H···Li	9	-132.5		C-H···F	9	-54.5
	C-H···S	10	-110.0		C-H···F	10	-60.0
	C-H···S	11	-111.0		C-H···F	11	-111.0
	C-H···S	12	-59.1		C-H···F	12	-72.5
Li_2S_2-co-C_4mpyr-*cis*-FTFSA	O···Li	1	-395.7	Li_2S_2-co-C_4mpyr-*trans*-FTFSA	C-H···S	1	-15.6
	O···Li	2	-399.0		C-H···S	2	-144.1
	C-H···S	3	-123.6		C-H···S	3	-92.1
	C-H···S	4	-109.6		C-H···S	4	-116.5
	C-H···O	5	-40.0		O···Li	5	-260.0
	C-H···O	6	-76.2		C-H···F	6	-25.2
	C-H···O	7	-79.7		C-H···F	7	-40.0
	C-H···O	8	-102.1		C-H···O	8	-106.6
	C-H···O	9	-147.5		C-H···O	9	-47.8
	C-H···O	10	-74.0		C-H···O	10	-79.4
					F···S	11	-83.8
C_4mpyr-*cis*-FTFSA-Li_2S_4	F···S	1	-18.8	C_4mpyr-*trans*-FTFSA-Li_2S_4	O···Li	1	-300.1
	O···S	2	-49.8		N···S	2	-65.4
	O···Li	3	-336.1		N···S	3	-65.4
	O···Li	4	-271.6		O···Li	4	-276.0
	C-H···F	5	-20.8		C-H···F	5	-19.4
	C-H···O	6	-31.0		C-H···O	6	-130.5
	C-H···F	7	-29.6		C-H···F	7	-40.1
	C-H···O	8	-136.5		C-H···F	8	-54.0
	C-H···N	9	-108.4		C-H···O	9	-167.2
	C-H···N	10	-162.9		C-H···O	10	-112.6
	C-H···O	11	-94.1		C-H···F	11	-67.9
	C-H···O	12	-104.4		C-H···O	12	-55.5
					C-H···O	13	-116.7
C_4mpyr-Li_2S_4-*cis*-FTFSA	O···Li	12	-305.1	C_4mpyr-Li_2S_4-*trans*-FTFSA	O···Li	1	-336.7
	O···S	13	-123.0		O···S	2	-125.4
	C-H···S	1	-35.7		O···S	3	-124.2
	O···Li	14	-328.4		O···Li	4	-274.3
	C-H···S	2	-61.6		F···S	5	-44.4
	C-H···S	3	-87.7		O···Li	6	-474.7
	O···Li	15	-496.8		C-H···S	7	-112.3
	C-H···S	5	-61.9		C-H···O	8	-52.2
	C-H···S	4	-111.9		C-H···O	9	-115.4
	C-H···S	6	-78.2		C-H···F	10	-38.0
	C-H···O	7	-52.0		C-H···S	11	-105.3
	C-H···O	8	-74.6		C-H···S	12	-83.7

	C-H···O	10	-52.4		C-H···S	13	-68.9
	C-H···O	9	-50.0		C-H···S	14	-50.1
	C-H···O	11	-52.8				
Li_2S_4-C_4mpyr-*cis*-FTFSA	C-H···S	1	-49.3	Li_2S_4-C_4mpyr-*trans*-FTFSA	C-H···S	1	-46.8
	C-H···Li	2	-136.3		C-H···S	2	-47.7
	C-H···S	3	-56.6		C-H···S	3	-67.1
	C-H···S	4	-61.1		C-H···S	4	-60.2
	C-H···S	5	-48.4		C-H···S	5	-121.5
	C-H···S	6	-69.4		C-H···S	6	-33.7
	C-H···S	7	-76.5		O···S	7	-19.0
	C-H···O	8	-46.4		O···S	8	-66.6
	C-H···O	9	-84.7		O···Li	9	-382.1
	C-H···O	10	-47.7		O···Li	10	-367.2
	C-H···O	11	-101.0		C-H···O	11	-46.2
	C-H···O	12	-106.4		C-H···O	12	-68.9
	C-H···N	13	-172.8		C-H···N	13	-89.7
	C-H···O	14	-115.8		C-H···O	14	-65.2
					C-H···F	15	-84.6
					C-H···F	16	-42.6
					C-H···O	17	-119.5
					C-H···F	18	-31.5
Li_2S_4-co-C_4mpyr-*cis*-FTFSA	C-H···O	1	-97.3	Li_2S_4-co-C_4mpyr-*trans*-FTFSA	O···Li	1	-409.9
	C-H···O	2	-73.1		O···Li	2	-378.3
	C-H···O	3	-101.9		C-H···S	3	-97.7
	C-H···O	4	-144.1		C-H···S	4	-80.3
	C-H···O	5	-38.2		C-H···S	5	-93.9
	C-H···O	6	-59.7		C-H···O	6	-47.8
	N···S	7	-50.2		C-H···O	7	-48.2
	O···Li	8	-383.0		C-H···N	8	-52.8
	O···Li	9	-385.0		C-H···N	9	-123.1
	N···S	10	-49.1		C-H···F	10	-51.7
	C-H···S	11	-126.4		C-H···F	11	-50.4
	C-H···S	12	-78.9		C-H···O	12	-93.3
	C-H···S	13	-37.5		C-H···F	13	-95.2
					C-H···O	14	-114.8
C_4mpyr-*cis*-FTFSA-Li_2S_6	C-H···S	1	-51.6	C_4mpyr-*trans*-FTFSA-Li_2S_6	C-H···F	1	-58.0
	C-H···S	2	-48.5		C-H···O	2	-155.5
	C-H···S	3	-58.2		C-H···O	3	-68.3
	C-H···S	4	-70.5		C-H···O	4	-120.8
	C-H···S	5	-45.7		C-H···O	5	-124.8
	O···S	6	-49.5		C-H···O	6	-114.7

	N···S	7	-293.2		C-H···F	7	-68.1
	N···Li	8	-301.8		F···S	8	-37.1
	N···S	9	-73.9		N···S	9	-70.4
	O···S	10	-41.1		O···Li	10	-263.2
	C-H···O	11	-95.4		N···Li	11	-179.5
	C-H···O	12	-38.9		N···S	12	-77.6
	C-H···O	13	-30.7		O···Li	13	-321.2
	C-H···O	14	-80.9				
	C-H···O	15	-75.5				
	C-H···O	16	-141.6				
	C-H···O	17	-62.4				
	C-H···O	118	-98.3				
C_4mpyr-Li_2S_6-*cis*-FTFSA	C-H···S	1	-39.1	C_4mpyr-Li_2S_6-*trans*-FTFSA	C-H···S	1	-72.4
	C-H···S	2	-46.8		C-H···S	2	-101.5
	C-H···S	3	-68.6		C-H···S	3	-92.5
	C-H···S	4	-97.9		C-H···S	4	-85.1
	C-H···S	5	-90.5		C-H···S	5	-59.1
	C-H···S	6	-71.9		C-H···S	6	-68.1
	O···S	7	-21.6		O···Li	7	-471.3
	O···S	8	-77.6		O···Li	8	-278.9
	O···Li	9	-475.4		O···S	9	-99.6
	O···Li	10	-413.3		O···Li	10	-442.2
	C-H···N	11	-122.8		O···S	11	-114.4
	C-H···O	12	-76.1		F···S	12	-27.7
	C-H···O	13	-146.1		C-H···F	13	-35.8
	C-H···O	14	-49.9				
	C-H···O	15	-63.8				
Li_2S_6-C_4mpyr-*cis*-FTFSA	C-H···S	1	-79.3	Li_2S_6-C_4mpyr-*trans*-FTFSA	C-H···S	1	-29.9
	C-H···S	2	-21.8		C-H···S	2	-63.7
	C-H···S	3	-74.0		C-H···S	3	-61.8
	C-H···S	4	-20.0		C-H···S	4	-41.7
	C-H···S	5	-52.8		C-H···S	5	-70.5
	C-H···S	6	-21.1		C-H···S	6	-43.3
	O···S	7	-72.8		C-H···S	7	-44.2
	C-H···O	8	-35.2		F···S	8	-90.1
	C-H···O	9	-78.2		F···Li	9	-308.5
	N···Li	10	-372.5		O···S	10	-83.5
	O···S	11	-77.4		O···Li	11	-383.6
	C-H···N	12	-62.6		C-H···O	12	-115.2
	C-H···O	13	-54.9		C-H···O	13	-139.0
	C-H···O	14	-75.0		C-H···O	14	-145.7
	C-H···O	15	-130.0		C-H···F	15	-51.2

	C-H···O	16	-72.3		C-H···O	16	-50.7
	C-H···O	17	-104.6		C-H···F	17	-44.1
Li_2S_6-co-C_4mpyr-*cis*-FTFSA	C-H···O	1	-88.5	Li_2S_6-co-C_4mpyr-*trans*-FTFSA	C-H···S	1	-48.4
	C-H···N	2	-47.8		C-H···S	2	-55.1
	C-H···O	3	-46.9		C-H···S	3	-105.5
	C-H···N	4	-70.5		C-H···S	4	-48.2
	C-H···O	5	-123.1		C-H···S	5	-51.6
	C-H···O	6	-145.4		C-H···S	6	-76.1
	C-H···S	7	-33.7		C-H···S	7	-51.3
	C-H···S	8	-35.7		C-H···S	8	-18.9
	C-H···S	9	-57.5		F···S	9	-47.5
	C-H···S	10	-43.2		C-H···O	10	-110.9
	C-H···S	11	-78.8		C-H···O	11	-78.2
	C-H···S	12	-35.8		C-H···F	12	-67.2
	C-H···S	13	-67.4		C-H···F	13	-69.0
					C-H···O	14	-93.5
					O···S	15	-111.4
					O···Li	16	-335.5
					O···Li	17	-292.0
					O···Li	18	-459.1
					O···S	19	-98.9
C_4mpyr-*cis*-FTFSA-Li_2S_8	C-H···S	1	-38.4	C_4mpyr-*trans*-FTFSA-Li_2S_8	C-H···S	1	-123.7
	C-H···S	2	-74.9		C-H···S	2	-67.7
	C-H···S	3	-22.2		C-H···F	3	-40.3
	C-H···S	4	-57.4		C-H···O	4	-70.1
	C-H···S	5	-28.1		C-H···F	5	-64.0
	C-H···S	6	-73.6		C-H···O	6	-89.9
	C-H···S	7	-31.1		C-H···F	7	-96.6
	O···Li	8	-362.1		C-H···O	8	-141.6
	O···S	9	-106.5		C-H···O	9	-68.0
	C-H···O	10	-37.8		N···S	10	-80.9
	C-H···O	11	-103.6		O···Li	11	-359.5
	C-H···O	12	-44.8		O···Li	12	-345.4
	C-H···O	13	-90.3		N···S	13	-102.5
	C-H···O	14	-64.2				
	C-H···N	15	-121.3				
	C-H···O	16	-112.5				
C_4mpyr-Li_2S_8-*cis*-FTFSA	N···S	1	-58.9	C_4mpyr-Li_2S_8-*trans*-FTFSA	C-H···S	1	-47.3
	O···S	2	-94.8		C-H···S	2	-89.5
	O···S	3	-99.8		C-H···S	3	-92.9
	O···Li	4	-142.9		C-H···S	4	-29.0

	O···Li	5	-334.1		C-H···S	5	-90.4
	O···S	6	-147.5		C-H···O	6	-139.4
	C-H···O	7	-63.5		C-H···O	7	-41.9
	C-H···O	8	-100.8		C-H···F	8	-27.3
	C-H···S	9	-61.0		C-H···N	9	-41.5
	C-H···S	10	-76.0		C-H···O	10	-108.6
	C-H···S	11	-35.5		C-H···O	11	-108.1
	C-H···S	12	-30.7		F···S	12	-31.3
	C-H···S	13	-88.7		O···Li	13	-487.3
	C-H···S	14	-45.9		O···S	14	-125.7
	C-H···S	15	-63.5		O···Li	15	-476.4
	C-H···O	16	-45.2		O···S	16	-119.5
	C-H···F	17	-76.2				
	C-H···F	18	-94.7				
Li_2S_8-C_4mpyr-*cis*-FTFSA	O···Li	1	-390.0	Li_2S_8-C_4mpyr-*trans*-FTFSA	F···S	1	-57.6
	O···Li	2	-402.9		F···S	2	-52.6
	O···S	3	-63.9		C-H···S	3	-42.6
	O···S	4	-91.4		O···Li	4	-522.1
	C-H···S	5	-74.2		C-H···S	5	-70.3
	C-H···S	6	-55.6		C-H···F	6	-25.0
	C-H···S	7	-60.8		C-H···F	7	-52.6
	C-H···S	8	-95.8		C-H···F	8	-49.4
	C-H···S	9	-23.0		C-H···F	9	-76.3
	C-H···S	10	-28.7		C-H···N	10	-129.3
	C-H···S	11	-40.9		C-H···O	11	-87.7
	C-H···S	12	-43.5		C-H···O	12	-107.5
	C-H···O	13	-27.1		C-H···N	13	-67.7
	C-H···O	14	-107.0		C-H···S	14	-57.6
	C-H···O	15	-81.3		C-H···S	15	-53.3
	C-H···O	16	-62.4		C-H···S	16	-43.0
	C-H···O	17	-148.2		C-H···S	17	-80.1
					C-H···S	18	-35.5
Li_2S_8-co-C_4mpyr-*cis*-FTFSA	O···Li	1	-374.9	Li_2S_8-co-C_4mpyr-*trans*-FTFSA	O···Li	1	-514.1
	O···Li	2	-340.1		F···S	2	-13.4
	O···S	3	-74.9		C-H···S	3	-29.6
	C-H···S	4	-41.7		O···S	4	-65.9
	C-H···S	5	-28.6		C-H···S	5	-52.6
	C-H···S	6	-76.2		C-H···S	6	-44.5
	C-H···S	7	-30.1		C-H···S	7	-40.3
	C-H···S	8	-117.5		C-H···S	8	-46.1
	C-H···S	9	-46.4		C-H···S	9	-36.5
	C-H···S	10	-24.0		C-H···F	10	-28.0

	C-H···S	11	-40.1		C-H···O	11	-163.9
	C-H···S	12	-39.7		C-H···O	12	-186.7
	C-H···O	13	-134.3		C-H···F	13	-54.0
	C-H···O	14	-52.1		C-H···O	14	-66.0
	C-H···N	15	-69.6		C-H···O	15	-155.8
	C-H···O	16	-94.2		C-H···O	16	-65.8
	C-H···O	17	-83.7				
	C-H···N	18	-87.9				
	C-H···O	19	-100.6				
C_4mpyr-*cis*-FTFSA-S_8	F···S	1	-53.4	C_4mpyr-*trans*-FTFSA-S_8	O···S	1	-83.2
	O···S	2	-75.8		O···S	2	-57.2
	N···S	3	-41.7		N···S	3	-57.8
	N···S	4	-46.0		F···S	4	-61.7
	O···S	5	-99.3		O···S	5	-29.7
	O···S	6	-78.5		F···S	6	-53.9
	N···S	7	-24.7		C-H···S	7	-51.9
	O···S	8	-53.0		C-H···S	8	-39.3
	C-H···S	9	-48.7		C-H···S	9	-44.2
	C-H···S	10	-31.1		C-H···S	10	-44.0
	C-H···S	11	-80.3		C-H···S	11	-40.6
	C-H···S	12	-22.5		C-H···S	12	-39.5
	C-H···O	13	-53.0		C-H···S	13	-33.6
	C-H···N	14	-112.4		C-H···O	14	-93.3
	C-H···O	15	-53.2		C-H···O	15	-165.9
	C-H···O	16	-73.0		C-H···F	16	-82.0
	C-H···O	17	-160.0		C-H···O	17	-150.9
	C-H···O	18	-82.4		C-H···O	18	-89.9
	C-H···O	19	-116.8		C-H···O	19	-95.7
	C-H···O	20	-85.7		C-H···O	20	-125.6
C_4mpyr-S_8-*cis*-FTFSA	C-H···S	1	-47.8	C_4mpyr-S_8-*trans*-FTFSA	F···S	1	-57.7
	C-H···S	2	-47.4		C-H···S	2	-89.0
	C-H···S	3	-68.4		O···S	3	-90.9
	C-H···S	4	-70.9		O···S	4	-101.3
	C-H···S	5	-47.9		O···S	5	-83.0
	C-H···S	6	-48.7		O···S	6	-91.1
	C-H···S	7	-68.5		O···S	7	-84.2
	C-H···S	8	-92.0		O···S	8	-115.2
	O···S	9	-68.6		C-H···S	9	-43.1
	O···S	10	-73.1		F···S	10	-103.5
	O···S	11	-82.7		C-H···S	11	-84.4
	O···S	12	-86.4		C-H···S	12	-55.3
	O···S	13	-70.4		C-H···S	13	-50.8

	O···S	14	-66.4		C-H···S	14	-64.0
	O···S	15	-122.3		C-H···S	15	-80.6
	O···S	16	-101.3		C-H···S	16	-73.5
	O···S	17	-109.2		C-H···S	17	-40.1
S_8-C_4mpyr-*cis*-FTFSA	C-H···S	1	-44.6	S_8-C_4mpyr-*trans*-FTFSA	F···S	1	-49.6
	C-H···S	2	-78.4		F···S	2	-56.5
	C-H···N	3	-171.9		F···S	3	-43.1
	C-H···S	3	-54.3		O···S	4	-18.9
	C-H···S	4	-56.4		C-H···S	5	-23.7
	C-H···S	5	-36.5		C-H···S	6	-24.7
	O···S	6	-81.9		C-H···S	7	-71.7
	O···S	7	-59.3		C-H···S	8	-27.6
	O···S	8	-85.9		C-H···S	9	-45.2
	C-H···O	9	-70.3		C-H···S	10	-55.4
	C-H···O	10	-160.2		C-H···S	11	-30.2
	C-H···N	11	-97.6		C-H···S	12	-49.6
	C-H···O	12	-118.1		C-H···S	13	-61.5
					C-H···S	14	-62.1
					C-H···O	15	-78.7
					C-H···O	16	-144.8
					C-H···O	17	-83.3
					C-H···O	18	-140.0
					C-H···O	19	-93.9
S_8-co-C_4mpyr-*cis*-FTFSA	N···S	1	-102.9	S_8-co-C_4mpyr-*trans*-FTFSA	F···S	1	-24.4
	O···S	2	-106.6		O···S	2	-46.9
	O···S	3	-91.1		F···S	3	-29.8
	C-H···S	4	-50.2		O···S	4	-85.7
	C-H···O	5	-30.6		O···S	5	-81.6
	C-H···O	6	-58.6		C-H···S	6	-53.1
	C-H···N	7	-89.6		C-H···S	7	-81.9
	C-H···O	8	-60.4		C-H···S	8	-46.8
	C-H···O	9	-96.5		C-H···O	9	-73.9
	C-H···O	10	-156.9		C-H···O	10	-199.8
	C-H···O	11	-83.1		C-H···F	11	-31.9
	C-H···O	12	-116.2		C-H···F	12	-105.5
	C-H···S	13	-45.6		C-H···O	13	-90.0
	C-H···S	14	-47.2		C-H···F	14	-64.7
	C-H···S	15	-19.0		C-H···F	15	-51.6
	C-H···S	16	-46.8		C-H···O	16	-129.2
	C-H···S	17	-23.5		C-H···O	17	-79.7
	C-H···S	18	-64.6		C-H···S	18	-25.2
	C-H···S	19	-45.4		C-H···S	19	-65.2

	C-H···S	20	-40.3		C-H···S	20	-25.2
					C-H···S	21	-28.8
					C-H···S	22	-34.0
C_4mpyr-*cis*-MCTFSA-Li_2S_1	O···Li	1	-513.3	C_4mpyr-*trans*-MCTFSA-Li_2S_1	C-H···S	1	-34.7
	O···Li	2	-499.4		O···Li	2	-528.5
	C-H···S	3	-107.1		C-H···S	3	-105.3
	C-H···S	4	-98.8		C-H···O	4	-39.0
	C-H···S	5	-164.0		C-H···S	5	-138.7
	C-H···N	6	-73.6		C-H···S	6	-35.6
	C-H···O	7	-126.3		O···Li	7	-537.3
	C-H···O	8	-56.2		C-H···O	8	-77.4
	C-H···O	9	-106.1		C-H···O	9	-30.0
					C-H···N	10	-164.6
					C-H···O	11	-38.7
					C-H···H	12	-30.3
C_4mpyr-Li_2S_1-*cis*-MCTFSA	C-H···S	1	-118.4	C_4mpyr-Li_2S_1-*trans*-MCTFSA	C-H···H	1	-12.8
	C-H···S	2	-155.0		C-H···O	2	-51.6
	C-H···S	3	-151.8		C-H···O	3	-94.1
	O···Li	4	-482.0		C-H···N	4	-63.4
	O···Li	5	-301.9		C-H···N	5	-61.0
	N···Li	6	-304.9		O···Li	6	-513.8
	C-H···O	7	-54.6		O···Li	7	-512.2
	C-H···N	8	-14.4		C-H···S	8	-130.9
	C-H···F	9	-81.5		C-H···S	9	-144.5
	C-H···O	10	-31.8		C-H···S	10	-101.7
	C-H···F	11	-46.8				
Li_2S_1-C_4mpyr-*cis*-MCTFSA	O···Li	1	-471.8	Li_2S_1-C_4mpyr-*trans*-MCTFSA	C-H···Li	1	-169.5
	N···Li	2	-313.0		C-H···S	2	-53.8
	O···Li	3	-286.0		C-H···S	3	-72.5
	C-H···S	4	-137.3		C-H···S	4	-120.0
	C-H···S	5	-117.6		C-H···O	5	-155.1
	C-H···S	6	-142.5		C-H···O	6	-74.5
	C-H···O	7	-47.7		C-H···O	7	-53.8
	C-H···O	8	-63.4		C-H···O	8	-90.7
	C-H···H	9	-22.4		C-H···N	9	-109.8
	C-H···O	10	-94.6		C-H···N	10	-127.6
	C-H···H	11	-17.8		C-H···N	11	-63.5
	C-H···O	12	-60.8		C-H···O	12	-136.2
Li_2S_1-co-C_4mpyr-*cis*-MCTFSA	C-H···S	1	-137.0	Li_2S_1-co-C_4mpyr-*trans*-MCTFSA	O···Li	1	-468.2
	C-H···S	2	-69.2		C-H···S	2	-131.5

	C-H···S	3	-172.1		C-H···S	3	-127.9
	C-H···O	4	-57.6		C-H···S	4	-30.4
	C-H···O	5	-61.6		C-H···O	5	-103.1
	O···Li	6	-474.0		N···Li	6	-284.3
	N···Li	7	-272.9		O···Li	7	-258.6
	O···Li	8	-293.5		C-H···O	8	-116.3
	C-H···O	9	-55.2		C-H···O	9	-96.3
	C-H···H	10	-15.6		C-H···O	10	-78.9
	C-H···O	11	-119.1				
C_4mpyr-*cis*-MCTFSA-Li_2S_2	C-H···O	1	-174.5	C_4mpyr-*trans*-MCTFSA-Li_2S_2	C-H···S	1	-79.5
	C-H···O	2	-66.8		C-H···O	2	-55.8
	C-H···O	3	-89.2		C-H···O	3	-101.5
	C-H···N	4	-37.5		C-H···O	4	-116.2
	C-H···O	5	-38.4		C-H···O	5	-66.6
	O···Li	6	-243.1		C-H···O	6	-53.5
	N···S	7	-85.8		C-H···O	7	-122.8
	N···Li	8	-284.7		C-H···O	8	-196.8
	O···Li	9	-434.4		C-H···F	9	-47.0
	C-H···S	10	-87.2		N···S	10	-90.9
	C-H···S	11	-136.0		O···Li	11	-237.9
	C-H···S	12	-92.1		N···Li	12	-260.2
					O···Li	13	-399.6
C_4mpyr-Li_2S_2-*cis*-MCTFSA	C-H···S	1	-145.7	C_4mpyr-Li_2S_2-*trans*-MCTFSA	C-H···F	1	-23.5
	C-H···S	2	-134.9		C-H···O	2	-134.7
	C-H···S	3	-98.8		C-H···O	3	-37.1
	C-H···O	4	-93.8		O···Li	4	-538.5
	C-H···O	5	-43.0		C-H···S	5	-135.3
	C-H···O	6	-72.2		C-H···S	6	-71.6
	O···Li	7	-307.7		C-H···S	7	-123.6
	N···Li	8	-411.7		F···S	8	-23.8
	O···Li	9	-460.8		O···Li	9	-359.2
					O···S	10	-128.7
					O···S	11	-131.7
					O···Li	12	-454.6
Li_2S_2-C_4mpyr-*cis*-MCTFSA	C-H···S	1	-26.6	Li_2S_2-C_4mpyr-*trans*-MCTFSA	O···Li	1	-589.0
	C-H···S	2	-100.8		C-H···S	2	-44.8
	C-H···S	3	-131.8		C-H···S	3	-110.6
	C-H···S	4	-124.0		C-H···S	4	-83.0
	C-H···O	5	-130.0		C-H···S	5	-65.0
	C-H···O	6	-86.9		C-H···O	6	-75.7
	N···Li	7	-459.5		C-H···N	7	-62.4

	F···S	8	-21.8		C-H···O	8	-63.6
	O···S	9	-115.1		C-H···O	9	-74.6
	O···Li	10	-421.7		C-H···N	10	-57.9
					C-H···O	11	-102.8
					C-H···N	12	-58.7
					C-H···O	13	-98.2
					C-H···N	14	-58.7
Li_2S_2-co-C_4mpyr-*cis*-MCTFSA	C-H···S	1	-49.1	Li_2S_2-co-C_4mpyr-*trans*-MCTFSA	C-H···S	1	-92.4
	C-H···S	2	-126.9		C-H···S	2	-76.4
	C-H···S	3	-141.6		C-H···S	3	-111.1
	C-H···O	4	-39.1		O···Li	4	-581.1
	O···Li	5	-282.4		C-H···O	5	-92.2
	N···Li	6	-304.0		C-H···N	6	-143.2
	N···S	7	-87.8		C-H···N	7	-74.4
	O···Li	8	-436.3		C-H···O	8	-45.1
	C-H···O	9	-186.0		C-H···O	9	-60.6
	C-H···O	10	-86.0		C-H···O	10	-72.9
	C-H···O	11	-81.1		C-H···O	11	-54.5
C_4mpyr-*cis*-MCTFSA-Li_2S_4	C-H···S	1	-53.8	C_4mpyr-*trans*-MCTFSA-Li_2S_4	O···Li	1	-312.6
	C-H···S	2	-38.7		N···Li	2	-249.1
	C-H···S	3	-42.8		N···S	3	-84.5
	C-H···O	4	-31.8		O···Li	4	-436.1
	N···S	5	-31.7		C-H···S	5	-21.6
	F···S	6	-32.8		C-H···O	6	-53.3
	C-H···O	7	-102.6		C-H···S	7	-112.2
	C-H···F	8	-36.7		C-H···S	8	-91.1
	C-H···H	9	-149.8		C-H···O	9	-116.0
	C-H···F	10	-48.3		C-H···O	10	-162.0
	C-H···N	11	-154.8		C-H···O	11	-73.4
	C-H···O	12	-109.5		C-H···O	12	-134.1
	C-H···O	13	-81.4		C-H···O	13	-61.5
	O···Li	14	-387.0				
	F···Li	15	-273.1				
	C-H···S	16	-44.1				
C_4mpyr-Li_2S_4-*cis*-MCTFSA	C-H···S	1	-37.9	C_4mpyr-Li_2S_4-*trans*-MCTFSA	O···Li	1	-456.8
	C-H···S	2	-58.3		O···S	2	-100.1
	C-H···S	3	-114.4		O···Li	3	-495.8
	C-H···S	4	-106.8		C-H···S	4	-102.1
	C-H···S	5	-50.5		C-H···O	5	-60.2
	C-H···S	6	-81.6		C-H···O	6	-133.3
	O···Li	7	-277.8		C-H···O	7	-114.2

	C-H···O	8	-45.8		C-H···S	8	-27.2
	C-H···O	9	-76.7		C-H···S	9	-81.7
	C-H···O	10	-89.2		C-H···S	10	-88.5
	N···Li	11	-418.8				
	N···S	12	-96.5				
	O···Li	13	-425.9				
Li_2S_4-C_4mpyr-*cis*-MCTFSA	C-H···S	1	-50.7	Li_2S_4-C_4mpyr-*trans*-MCTFSA	C-H···S	1	-66.9
	C-H···S	2	-59.4		C-H···S	2	-38.7
	C-H···S	3	-57.2		O···Li	3	-385.3
	C-H···S	4	-53.2		N···Li	4	-304.4
	C-H···S	5	-77.1		O···Li	5	-374.3
	C-H···O	6	-145.6		N···S	6	-37.6
	C-H···O	7	-56.6		C-H···O	7	-95.9
	C-H···O	8	-103.4		C-H···N	8	-21.9
	C-H···N	9	-129.2		C-H···O	9	-58.5
	C-H···N	10	-123.9		C-H···N	10	-37.5
	C-H···O	11	-178.5		C-H···O	11	-73.7
					C-H···O	12	-123.8
					C-H···H	13	-34.2
					C-H···O	14	-76.1
					C-H···S	15	-45.5
					C-H···S	16	-53.1
					C-H···S	17	-92.1
Li_2S_4-co-C_4mpyr-*cis*-MCTFSA	O···Li	1	-370.1	Li_2S_4-co-C_4mpyr-*trans*-MCTFSA	O···Li	1	-422.5
	N···Li	2	-275.7		O···Li	2	-444.2
	C-H···N	3	-25.8		O···S	3	-85.2
	C-H···O	4	-176.8		C-H···O	4	-71.8
	C-H···O	5	-90.1		C-H···O	5	-71.4
	C-H···O	6	-134.8		C-H···O	6	-62.7
	C-H···O	7	-39.5		C-H···S	7	-81.1
	C-H···S	8	-38.7		C-H···S	8	-118.5
	C-H···S	9	-105.8		C-H···S	9	-88.6
	C-H···S	10	-80.0		C-H···N	10	-62.0
					C-H···N	11	-145.9
					C-H···N	12	-52.1
					C-H···O	13	-66.7
					C-H···O	14	-105.0
C_4mpyr-*cis*-MCTFSA-Li_2S_6	C-H···S	1	-28.4	C_4mpyr-*trans*-MCTFSA-Li_2S_6	O···S	1	-117.2
	O···Li	2	-398.7		O···Li	2	-394.1
	F···Li	3	-324.0		O···Li	3	-320.1
	F···S	4	-46.2		C-H···S	4	-70.7

	F···S	5	-41.7		O···Li	5	-490.9
	N···S	6	-48.3		O···S	6	-103.7
	F···S	7	-27.2		C-H···N	7	-147.7
	C-H···O	8	-138.2		C-H···O	8	-129.0
	C-H···S	9	-35.0		C-H···O	9	-71.9
	C-H···S	10	-56.5		C-H···S	10	-88.9
	C-H···N	11	-79.6		C-H···O	11	-59.0
	C-H···F	12	-42.9		C-H···S	12	-82.9
	C-H···F	13	-102.4		C-H···O	13	-37.4
	C-H···N	14	-125.1		C-H···S	14	-38.7
	C-H···N	15	-150.5		C-H···S	15	-36.7
	C-H···O	16	-71.5		C-H···S	16	-66.9
					C-H···S	17	-27.3
C_4mpyr-Li_2S_6-*cis*-MCTFSA	O···Li	1	-436.5	C_4mpyr-Li_2S_6-*trans*-MCTFSA	O···S	1	-110.3
	O···Li	2	-511.5		O···Li	2	-411.0
	O···S	3	-77.1		C-H···S	3	-39.1
	C-H···S	4	-48.6		O···Li	4	-358.3
	C-H···S	5	-44.2		O···S	5	-132.3
	C-H···S	6	-56.5		O···Li	6	-523.8
	C-H···S	7	-60.9		O···S	7	-49.3
	C-H···S	8	-90.1		C-H···N	8	-23.6
	C-H···S	9	-66.1		C-H···S	9	-55.3
	C-H···N	10	-57.4		C-H···S	10	-108.6
	C-H···N	11	-126.1		C-H···S	11	-78.4
	C-H···O	12	-146.2		C-H···S	12	-44.2
	C-H···O	13	-119.6				
Li_2S_6-C_4mpyr-*cis*-MCTFSA	C-H···Li	1	-157.4	Li_2S_6-C_4mpyr-*trans*-MCTFSA	O···S	1	-65.5
	C-H···S	2	-70.4		O···Li	2	-283.4
	C-H···S	3	-79.1		O···Li	3	-331.7
	C-H···S	4	-22.2		O···S	4	-77.2
	C-H···S	5	-27.4		C-H···S	5	-38.6
	C-H···S	6	-44.8		C-H···S	6	-77.3
	C-H···S	7	-64.0		C-H···S	7	-79.4
	C-H···S	8	-31.7		C-H···S	8	-53.8
	C-H···O	9	-131.0		C-H···S	9	-55.6
	C-H···O	10	-81.1		C-H···S	10	-41.5
	C-H···N	11	-148.1		C-H···N	11	-110.7
	C-H···N	12	-156.4		C-H···N	12	-171.3
	C-H···O	13	-172.0		C-H···O	13	-93.9
	C-H···O	14	-70.4				
Li_2S_6-co-C_4mpyr-*cis*-MCTFSA	C-H···S	1	-30.7	Li_2S_6-co-C_4mpyr-*trans*-MCTFSA	O···Li	1	-420.7

	C-H···S	2	-51.2		O···Li	2	-604.5
	C-H···S	3	-27.7		O···S	3	-75.4
	C-H···S	4	-44.7		O···S	4	-37.8
	C-H···S	5	-68.0		C-H···O	5	-87.6
	C-H···S	6	-88.7		C-H···S	6	-28.0
	C-H···S	7	-80.8		C-H···S	7	-80.8
	C-H···O	8	-130.9		C-H···N	8	-160.2
	C-H···O	9	-124.4		C-H···N	9	-66.7
	C-H···O	10	-23.4		C-H···O	10	-62.8
	O···Li	11	-283.0		C-H···S	11	-56.7
	N···Li	12	-330.4		C-H···S	12	-53.7
	N···S	13	-98.6		C-H···S	13	-81.0
	O···Li	14	-447.1		C-H···S	14	-64.1
					C-H···S	15	-45.5
C_4mpyr-*cis*-MCTFSA-Li_2S_8	C-H···S	1	-56.3	C_4mpyr-*trans*-MCTFSA-Li_2S_8	O···Li	1	-537.7
	C-H···S	2	-81.6		O···Li	2	-473.7
	C-H···S	3	-27.3		O···S	3	-60.4
	C-H···O	4	-95.4		O···S	4	-42.9
	C-H···O	5	-113.3		C-H···S	5	-52.5
	C-H···O	6	-82.1		C-H···N	6	-160.0
	C-H···H	7	-18.9		C-H···O	7	-99.7
	C-H···O	8	-60.5		C-H···O	8	-177.4
	C-H···N	9	-110.1		C-H···S	9	-111.8
	C-H···H	10	-34.9		C-H···S	10	-43.3
	C-H···O	11	-49.3		C-H···S	11	-76.7
	C-H···O	12	-25.7		C-H···S	12	-43.1
	O···S	13	-117.9				
	N···S	14	-37.6				
	N···Li	15	-338.1				
	O···Li	16	-280.8				
	O···S	17	-79.0				
C_4mpyr-Li_2S_8-*cis*-MCTFSA	C-H···S	1	-26.4	C_4mpyr-Li_2S_8-*trans*-MCTFSA	O···S	1	-55.4
	C-H···S	2	-71.8		O···Li	2	-282.7
	C-H···S	3	-28.0		N···S	3	-93.8
	C-H···S	4	-54.9		N···Li	4	-261.9
	C-H···S	5	-98.0		O···Li	5	-425.3
	C-H···S	6	-102.6		O···S	6	-106.4
	C-H···O	7	-68.7		N···S	7	-94.5
	C-H···N	8	-92.1		C-H···O	8	-112.5
	C-H···O	9	-82.9		C-H···S	9	-87.9
	C-H···O	10	-150.9		C-H···O	10	-112.6
	C-H···O	11	-53.3		C-H···O	11	-120.6

	O···S	12	-75.3		C-H···S	12	-97.6
	O···Li	13	-449.5		C-H···S	13	-95.4
	O···S	14	-105.8		C-H···H	14	-35.2
	O···Li	15	-483.4				
	O···S	16	-83.8				
	F···S	17	-43.8				
Li_2S_8-C_4mpyr-*cis*-MCTFSA	C-H···S	1	-44.1	Li_2S_8-C_4mpyr-*trans*-MCTFSA	C-H···S	1	-53.2
	C-H···S	2	-51.9		C-H···S	2	-32.9
	C-H···Li	3	-141.0		C-H···S	3	-28.3
	C-H···S	4	-48.3		C-H···S	4	-48.1
	C-H···S	5	-62.4		C-H···S	5	-65.2
	C-H···S	6	-85.2		C-H···S	6	-77.0
	C-H···F	7	-55.9		C-H···S	7	-56.2
	C-H···O	8	-95.4		C-H···S	8	-36.2
	C-H···O	9	-111.9		C-H···S	9	-46.8
	C-H···N	10	-168.9		O···Li	10	-284.5
	C-H···N	11	-181.0		N···Li	11	-380.7
	C-H···O	12	-185.2		C-H···O	12	-201.4
					C-H···O	13	-114.0
					C-H···O	14	-154.7
Li_2S_8-co-C_4mpyr-*cis*-MCTFSA	C-H···S	1	-63.1	Li_2S_8-co-C_4mpyr-*trans*-MCTFSA	F···S	1	-16.5
	O···S	2	-68.7		O···Li	2	-615.5
	C-H···S	3	-51.9		O···S	3	-22.5
	O···Li	4	-204.4		C-H···S	4	-13.6
	O···Li	5	-510.7		C-H···S	5	-30.5
	C-H···S	6	-52.3		C-H···S	6	-88.3
	C-H···S	7	-49.8		C-H···S	7	-37.0
	C-H···S	8	-68.5		C-H···S	8	-43.7
	C-H···S	9	-27.7		C-H···S	9	-73.5
	C-H···S	10	-31.1		C-H···S	10	-43.9
	C-H···S	11	-119.9		C-H···O	11	-64.9
	C-H···S	12	-42.1		C-H···O	12	-176.5
	C-H···S	13	-47.0		C-H···O	13	-48.0
	C-H···O	14	-17.6		C-H···N	14	-92.8
	C-H···N	15	-121.7		C-H···N	15	-178.7
	C-H···O	16	-78.0				
	C-H···O	17	-69.6				
	C-H···N	18	-146.3				
	C-H···O	19	-118.8				
C_4mpyr-*cis*-MCTFSA-S_8	C-H···S	1	-54.0	C_4mpyr-*trans*-MCTFSA-S_8	O···S	1	-117.2
	C-H···S	2	-48.7		C-H···S	2	-34.8

	C-H···S	3	-28.7		O···S	3	-142.9
	O···S	4	-91.1		C-H···S	4	-57.6
	C-H···O	5	-99.4		C-H···S	5	-18.0
	C-H···O	6	-130.1		C-H···S	6	-35.2
	C-H···F	7	-52.6		C-H···S	7	-45.1
	C-H···N	8	-131.8		C-H···S	8	-43.1
	C-H···N	9	-115.1		C-H···S	9	-29.4
	C-H···N	10	-92.8		C-H···S	10	-48.3
	C-H···O	11	-98.1		C-H···H	11	-28.6
	C-H···O	12	-92.7		C-H···O	12	-90.5
	C-H···O	13	-118.6		C-H···N	13	-81.2
	F···S	14	-12.6		C-H···O	14	-74.8
	F···S	15	-48.2		C-H···O	15	-102.1
	C-H···S	16	-30.9		C-H···N	16	-183.5
	O···S	17	-48.2		C-H···O	17	-129.6
	F···S	18	-51.7				
	F···S	19	-53.9				
C_4mpyr-S_8-*cis*-MCTFSA	C-H···S	1	-52.8	C_4mpyr-S_8-*trans*-MCTFSA	C-H···S	1	-44.9
	C-H···S	2	-65.7		C-H···S	2	-59.8
	C-H···S	3	-71.8		C-H···S	3	-49.1
	C-H···S	4	-38.7		C-H···S	4	-70.9
	C-H···S	5	-52.3		C-H···S	5	-64.6
	C-H···S	6	-56.4		C-H···S	6	-70.1
	C-H···S	7	-88.2		C-H···S	7	-93.5
	C-H···S	8	-67.5		C-H···S	8	-53.1
	O···S	9	-134.4		F···S	9	-37.1
	O···S	10	-121.1		O···S	10	-117.4
	N···S	11	-61.8		O···S	11	-122.8
	N···S	12	-63.7		O···S	12	-124.1
	N···S	13	-53.6		O···S	13	-141.7
	N···S	14	-61.5		F···S	14	-61.3
	F···S	15	-38.6		O···S	15	-113.5
	O···S	16	-122.8		O···S	16	-115.2
	O···S	17	-137.1		O···S	17	-90.3
S_8-C_4mpyr-*cis*-MCTFSA	C-H···S	1	-18.6	S_8-C_4mpyr-*trans*-MCTFSA	N···S	1	-85.6
	C-H···S	2	-66.2		O···S	2	-132.9
	C-H···S	3	-50.7		C-H···O	3	-148.5
	C-H···S	4	-29.4		C-H···O	4	-43.1
	C-H···S	5	-60.4		C-H···S	5	-41.8
	C-H···S	6	-45.5		C-H···S	6	-62.2
	C-H···S	7	-35.0		C-H···S	7	-59.8
	C-H···S	8	-40.7		C-H···S	8	-49.7

	C-H···F	9	-49.0		C-H···S	9	-25.4
	C-H···O	10	-51.8		C-H···S	10	-27.2
	C-H···O	11	-115.4		C-H···N	11	-153.5
	C-H···N	12	-158.0		C-H···O	12	-99.9
	C-H···N	13	-194.8		C-H···N	13	-97.3
	C-H···O	14	-181.7		C-H···O	14	-131.3
					C-H···O	15	-65.6
S_8-co-C_4mpyr-*cis*-MCTFSA	C-H···S	1	-22.6	S_8-co-C_4mpyr-*trans*-MCTFSA	C-H···S	1	-29.1
	C-H···S	2	-64.3		O···S	2	-136.5
	C-H···S	3	-41.5		C-H···S	3	-48.1
	C-H···S	4	-37.5		C-H···S	4	-56.4
	C-H···S	5	-65.8		C-H···S	5	-27.2
	O···S	6	-118.5		C-H···S	6	-49.0
	N···S	7	-122.5		C-H···S	7	-29.5
	F···S	8	-46.4		C-H···S	8	-63.6
	C-H···O	9	-95.3		C-H···S	9	-44.0
	C-H···N	10	-158.4		C-H···S	10	-38.8
	C-H···O	11	-68.0		C-H···H	11	-36.6
	C-H···N	12	-89.6		C-H···N	12	-106.8
	C-H···O	13	-155.3		C-H···O	13	-64.3
	C-H···N	14	-69.5		C-H···O	14	-157.7
	C-H···O	15	-85.5		C-H···N	15	-181.4
					C-H···O	16	-132.0

Note S1. Parameter definitions for formulas in CDA analysis.

$$d_i = \sum_{m \in A}^{\text{occ}} \sum_{n \in B}^{\text{vir}} \eta_i C_{m,i} C_{n,i} S_{m,n} \qquad \text{(S1)}$$

$$b = \sum_{m \in A}^{\text{vir}} \sum_{n \in B}^{\text{occ}} \eta_i C_{m,i} C_{n,i} S_{m,n} \qquad \text{(S2)}$$

$$r_i = \sum_{m \in A}^{\text{occ}} \sum_{n \in B}^{\text{occ}} \eta_i C_{m,i} C_{n,i} S_{m,n} \qquad \text{(S3)}$$

The symbol '*i*' denotes the label for the intricate orbital, while '*m*' and '*n*' correspond to the tracks of fragments A and B, respectively. The terms '*occ*' and '*vir*' signify the collective sums for occupied and non-occupied orbitals, respectively. The parameter 'η' represents the occupancy number of the intricate orbital. The

matrix elements of '*C*' pertaining to 'm' and '*i*' denote the coefficients denoting the contribution of fragment track 'm' to the intricate track '*i*'. '*S*' denotes the matrix of overlap between the fragment tracks. For the '*r*' term, when the sum of all 'r_i' values is negative, epitomizes electron transfer from the overlapping region, thereby revealing the exclusionary effect of electrons. Conversely, if the sum is positive, it signifies the overlap of electrons in the interacting region, exhibiting bonding characteristics of the orbitals.

$$t_i = \sum_{m\in A}\sum_{n\in B} \eta_i \frac{|\eta_m^{\mathrm{FO}} - \eta_n^{\mathrm{FO}}|}{\eta_{\mathrm{ref}}} C_{m,i} C_{n,i} S_{m,n} \tag{S4}$$

$$r_i = \sum_{m\in A}\sum_{n\in B} 2\frac{\min(\eta_m^{\mathrm{FO}}, \eta_n^{\mathrm{FO}})}{\eta_{\mathrm{ref}}} \eta_i C_{m,i} C_{n,i} S_{m,n} \tag{S5}$$

For the open-shell system, the complex orbital reference occupancy η_{ref} is 1 and CDA processes α and β spintrons alone, in the process of calculating *t*, if $\eta_m^{FO} > \eta_n^{FO}$, *t* is equivalent to *d* in the initial definition; otherwise, *t* is equivalent to *b*. Both *b* and *d* have contributions from charge transfer and polarization. The "min" in the *r* expression represents the function used to extract the minimum from two values.
